# Roflumilast inhibits tumor growth and migration in STK11/LKB1 deficient pancreatic cancer

**DOI:** 10.1038/s41420-024-01890-y

**Published:** 2024-03-09

**Authors:** Shuman Zhang, Duo Yun, Hao Yang, Markus Eckstein, Gihan Daw Elbait, Yaxing Zhou, Yanxi Lu, Hai Yang, Jinping Zhang, Isabella Dörflein, Nathalie Britzen-Laurent, Susanne Pfeffer, Marc P. Stemmler, Andreas Dahl, Debabrata Mukhopadhyay, David Chang, Hang He, Siyuan Zeng, Bin Lan, Benjamin Frey, Chuanpit Hampel, Eva Lentsch, Paradesi Naidu Gollavilli, Christian Büttner, Arif B. Ekici, Andrew Biankin, Regine Schneider-Stock, Paolo Ceppi, Robert Grützmann, Christian Pilarsky

**Affiliations:** 1 Department of Surgery, Universitätsklinikum Erlangen, Friedrich-Alexander Universität Erlangen-Nürnberg (FAU), Erlangen, Germany; 2 Experimental Tumor pathology, Institute of Pathology, Universitätsklinikum Erlangen, Friedrich-Alexander Universität Erlangen-Nürnberg (FAU), Erlangen, Germany; 3 Institute of Pathology, Universitätsklinikum Erlangen, Friedrich-Alexander-Universität Erlangen-Nürnberg (FAU), Erlangen, Germany; 4 Department of Biology, Khalifa University of Science and Technology, Abu Dhabi, United Arab Emirates; 5 Department of Experimental Medicine 1, Nikolaus-Fiebiger Center for Molecular Medicine, Friedrich-Alexander-Universität Erlangen-Nürnberg (FAU), Erlangen, Germany; 6 DRESDEN-concept Genome Center a DFG NGS Competence Center; TU Dresden, 01307 Dresden, Germany; 7 Departments of Biochemistry and Molecular Biology, Mayo Clinic College of Medicine and Science, Jacksonville, USA; 8 Wolfson Wohl Cancer Research Centre, Institute of Cancer Sciences, University of Glasgow, Glasgow, Scotland UK; 9 West of Scotland Pancreatic Unit, Glasgow Royal Infirmary, Glasgow, UK; 10 Department of Pancreatic Surgery, Huashan Hospital, Shanghai Medical College, Fudan University, Shanghai, 200040 China; 11 Department of Breast and Thyroid Surgery, Hunan Provincial People’s Hospital (The First Affiliated Hospital of Hunan Normal University), Changsha, China; 12 Department of Interventional Radiology and Vascular Surgery, Hunan Provincial People’s Hospital (The First Affiliated Hospital of Hunan Normal University), Changsha, 410002 China; 13 Translational Radiobiology, Department of Radiation Oncology, Universitätsklinikum Erlangen, Friedrich-Alexander Universität Erlangen-Nürnberg (FAU), Erlangen, Germany; 14 Department of Biochemistry and Molecular Biology (BMB), University of Southern Denmark, Odense, Denmark; 15 Institute of Human Genetics, Friedrich-Alexander-Universität Erlangen-Nürnberg (FAU), Erlangen, Germany

**Keywords:** Pancreatic cancer, Targeted therapies

## Abstract

Pancreatic cancer is a malignant tumor of the digestive system. It is highly aggressive, easily metastasizes, and extremely difficult to treat. This study aimed to analyze the genes that might regulate pancreatic cancer migration to provide an essential basis for the prognostic assessment of pancreatic cancer and individualized treatment. A CRISPR knockout library directed against 915 murine genes was transfected into TB 32047 cell line to screen which gene loss promoted cell migration. Next-generation sequencing and PinAPL.py- analysis was performed to identify candidate genes. We then assessed the effect of serine/threonine kinase 11 (STK11) knockout on pancreatic cancer by wound-healing assay, chick agnosia (CAM) assay, and orthotopic mouse pancreatic cancer model. We performed RNA sequence and Western blotting for mechanistic studies to identify and verify the pathways. After accelerated Transwell migration screening, STK11 was identified as one of the top candidate genes. Further experiments showed that targeted knockout of STK11 promoted the cell migration and increased liver metastasis in mice. Mechanistic analyses revealed that STK11 knockout influences blood vessel morphogenesis and is closely associated with the enhanced expression of phosphodiesterases (PDEs), especially PDE4D, PDE4B, and PDE10A. PDE4 inhibitor Roflumilast inhibited STK11-KO cell migration and tumor size, further demonstrating that PDEs are essential for STK11-deficient cell migration. Our findings support the adoption of therapeutic strategies, including Roflumilast, for patients with STK11-mutated pancreatic cancer in order to improve treatment efficacy and ultimately prolong survival.

## Introduction

According to estimates from the Global Cancer Statistics [1], pancreatic malignancies claimed 466,003 lives in 2020, making it the sixth most common cause of cancer-related death among both sexes. The incidence of this disease has increased rapidly owing to its inconspicuous early symptoms, lack of diagnostic means, poor specificity of tumor markers, and frequent occurrence of early lymph node and distant metastases. Pancreatic cancer is highly metastatic even in the early stages, and local or metastatic recurrence can eventually lead to death. Indeed, approximately 50% of patients with pancreatic cancer already have distant metastases at the time of diagnosis, and the subsequent median survival is less than one year [2]. The most common types of pancreatic cancer therapy include surgery, chemotherapy, radiation therapy, targeted therapy, and supportive/palliative care. Surgery is still the only effective way to cure pancreatic cancer; however, the treatment eligibility rates are low.

Serine/threonine kinase 11 (STK11), also known as liver kinase B1 (LKB1), is associated with pancreatic cancer. STK11 was first shown to be mutated in Peutz–Jeghers syndrome and was later discovered to be an essential tumor suppressor [3]. An increasing amount of evidence suggests that inactivated STK11 somatic mutations contribute to the pathogenesis of numerous cancers, such as gastrointestinal cancer [4], non-small cell lung cancer (NSCLC) [5–7], pancreatic cancer [8], cervical cancer [9, 10], and melanoma [11], which are influenced by inactivated STK11 somatic mutations. To date, the mechanisms underlying STK11 loss-dependent migration are limited. However, some studies have highlighted the possible involvement of the STK11 pathway in metastasis as well as potential drugs and inhibitors. For example, STK11 increases the interaction of Snail with the E3 ligase FBXL14, thereby increasing ubiquitin-mediated Snail degradation in response to metformin treatment [8]. However, investigations of the mechanisms underlying STK11 loss-dependent migration are limited.

The primary treatment for metastatic pancreatic cancer is a combination of cytotoxic chemotherapies. The clinical significance of most genetic variants remains unclear, which limits the application of targeted therapy in the clinical diagnosis and treatment of pancreatic cancer. Targeted drugs commonly used for the treatment of pancreatic cancer include epidermal growth factor receptor (EGFR) inhibitors in patients with KRAS WT, poly (adenosine diphosphate-ribose) polymerase (PARP) inhibitors in patients with BRCA mutations and neurotrophic tyrosine receptor kinase (NTRK) inhibitors [12, 13]. The advent of targeted drugs has provided new treatment options for many patients with pancreatic cancer, significantly increasing their overall survival. STK11, a classic tumor suppressor, is mutated in pancreatic cancer and many other types of cancers. Somatic STK11 mutations have been observed in approximately 4% of sporadic PDAC [14–17]. However, there are still no effective targeted drugs for patients with STK11 mutations. Here, we identified PDE4, as potential drug targets in STK11-mutated pancreatic cancer.

PDE4, an enzyme that breaks down cyclic AMP in cells, influences various cellular processes such as inflammation, immune response, and cell proliferation [18]. These processes are critical in the development and progression of cancer, including pancreatic cancer. Roflumilast, as a PDE4 inhibitor, primarily known for its role in treating chronic obstructive pulmonary disease (COPD), could be a valuable therapeutic agent in cancer treatment, especially in specific types like B-cell malignancies and lung cancer [19, 20]. In summary, the application of Roflumilast on STK11-mutant pancreatic cancer cell lines, as explored in this study, lays a foundational platform for future clinical research. This could potentially offer substantial benefits to patients with such specific cancer profiles. The insights gained from these investigations are crucial for developing novel therapeutic strategies for treating STK11-mutated pancreatic cancers, where innovative and effective treatments are urgently needed.

## Results

### Functional selection of cell migration regulators in PDAC

**Fig. 1:**
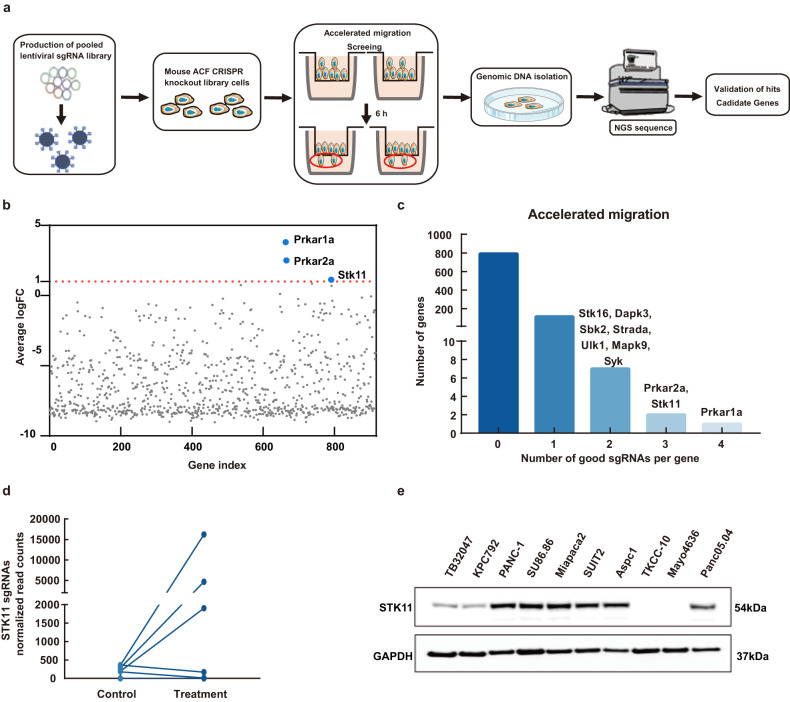
Results of mouse ACF CRISPR knockout library screening conducted to identify which genes can affect migration in PDAC. **a** Overview of the CRISPR/Cas9 screening procedure is provided in the Methods section. **b** Plot showing the enrichment of the top candidate genes in the accelerated migration group (blue dots) identified by PinAPL.py - compared to other genes in the library (gray dots) after selection. **c** The number of genes with zero, one, two, three, or four significantly enriched sgRNAs (*p* < 0.05 for three separate screening) targeting that gene in the accelerated migration group. Genes are categorized by the number of enriched sgRNAs, as indicated in the colored bubbles adjacent to each bar. **d** *STK11* have multiple enriched sgRNAs in the accelerated migration cells (Control Group: library cells before screening; Treatment Group: library cells after screening). **e** Basal expression of STK11 in 10 different PDAC cell lines was detected by western blot. GAPDH was used as a loading control.

To investigate cell migration regulators in PDAC, we prepared 450,000 library cells in serum-free medium, distributed over three Transwell FluoroBlok inserts. After 6 hours, we isolated a subset (1% of the total) from the lower compartments, which showed accelerated migration (Fig. 1a). This method allowed focused analysis of cells with enhanced migratory behavior in PDAC. In the supplemental table of full sgRNA screening results and Fig. 1b, protein kinase cAMP-dependent type I and type II regulatory subunit alpha (Prkar1a and Prkar2a) and Stk11 were identified as the top positive genes following Transwell selection (Avg logFC > 1, analyzed using PinAPL-). After screening, only 125 genes had one or more good sgRNAs (Fig. 1c). Three of the five *Stk11*-targeting sgRNAs were found to be highly enriched after screening (Fig. 1d). The basal expression of STK11 was detected using western blotting (Fig. 1f). STK11 was almost completely lost in TKCC-10 and Mayo4636 cell lines, whereas it was highly expressed in the PANC-1 cells.

### *STK11* knockout promotes cell migration in vitro

**Fig. 2:**
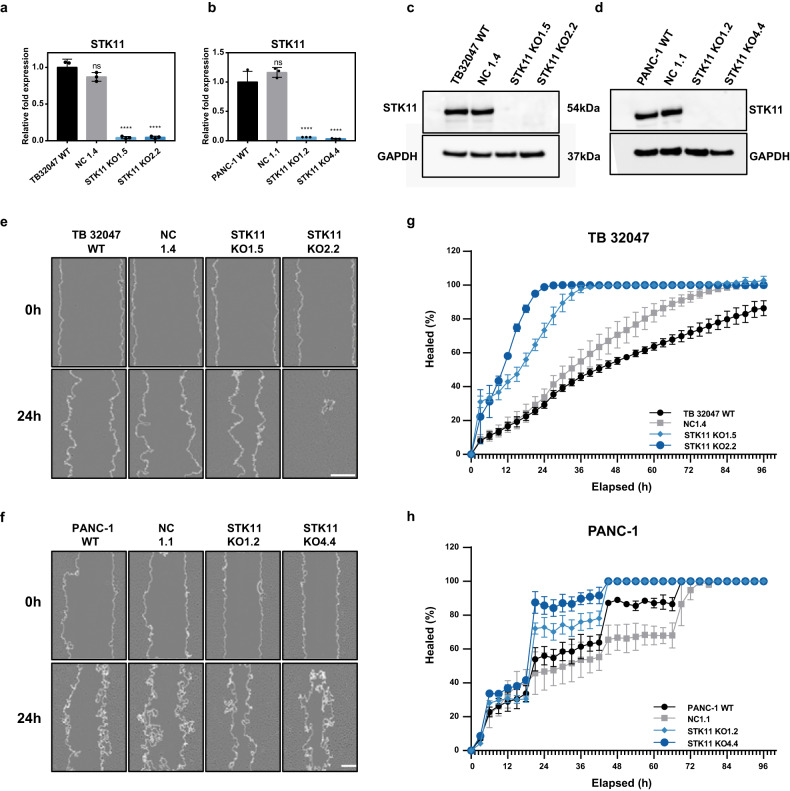
Validation of the gene targets determined by functional screening. **a**, **b** The *STK11* mRNA level was examined in TB 32047 and PANC-1 cells. Data are presented as means of three independent experiments; ****p < 0.0001 by one-way ANOVA. **c**, **d** STK11^KO^ TB 32047 and PANC-1 cells were generated using CRISPR/Cas9 technology. Western blot determined STK11 expression in TB 32047 and PANC-1 samples. KO1.5 was obtained from sgRNA1, and the single-clone number was 5. **e**, **f** Representative pictures of the wound-healing assay of the TB 32047 and PANC-1 cells. Scale bar: 200 µm. **g**, **h** 96-well IncuCyte^®^ scratch wound assay created a cell-free zone (wound) in a confluent cell monolayer of the TB 32047 and PANC-1 cells. Relative wound area was measured every 3 h and visualized in time–course plots. Average ± SEM (*n* = 3, each averaged from three technical replicates across three independent experiments) is shown.

Cellular migration was significantly increased in association with low STK11 expression in TB 32047 and PANC-1 cells (Supplementary Fig. 1). To further determine the role of STK11 in PDAC migration, STK11 knockout (KO) was generated in five PDAC cell lines (Fig. 2a–d; Supplementary Fig. 5o, p, q). All knockouts were verified by Sanger sequence to determine the type of mutation (Supplementary Table 5). IncuCyte^®^ scratch wound assay showed that STK11^KO^ significantly promoted PDAC cell motility (Fig. 2e, f). For the TB 32047 cell line, TB KO1.5, and TB KO2.2 healed 73.39% and 98.84% of the wound, respectively, after 24 h, whereas WT and NC1.4 cells healed less than 35% of the wound area (Fig. 2g). In the PANC-1 cell line, PANC KO1.2 and PANC KO4.4 healed 72.81% and 85.76% of the wound, respectively, after 24 h, whereas control cells healed less than 60% of the wound area (Fig. 2h).

### *STK11*-deleted clones show pronounced tumor aggressiveness in vivo

**Fig. 3:**
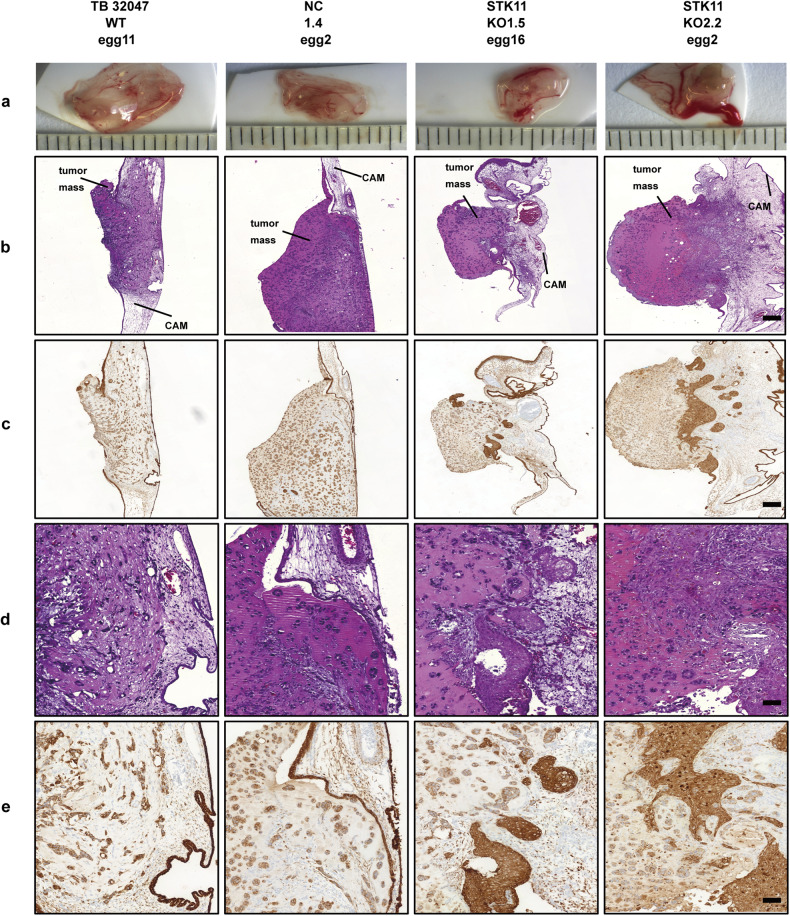
Growth and aggressiveness of STK11^KO^ cells in the CAM xenograft assay. **a** Ex ovo images of microtumors harvested 5 days post engraftment on CAM of fertilized chicken eggs. **b** Overview of H&E-stained microtumor sections. Scale: 200 μm. **c** Overview of Pan-cytokeratin immunostaining microtumor sections. Scale: 200 μm. **d** Exemplary images of H&E staining in microtumors of TB 32047 cells. Scale: 50 μm. **e** Exemplary images of Pan-cytokeratin immunostaining in microtumors of TB 32047 cells. Scale: 50 μm.

A chicken CAM xenograft assay was performed to confirm the more aggressive and invasive phenotype of TB 32047 cells (Fig. 3). Representative overview images of the different growth patterns of tumors are shown in Fig. 3a. Only TB KO1.5, showed a significantly larger tumor than the control group (Supplementary Fig. 2). However, tumors formed by TB KO1.5 and TB KO2.2 also showed aggressive growth, which could be observed by simple macroscopic and microscopic investigation of CAM microtumors (Fig. 3b–e). A transparent or intact CAM layer was observed in the control group when tumor sections stained with H&E and Pan-cytokeratin were evaluated. Compared to controls, TB KO1.5 and TB KO2.2 cells significantly damaged the CAM layer, with highly infiltrative growth at the invasive tumor front and considerable interaction/mixing with the CAM tissue throughout.

### *STK11* inhibited PDAC metastasis in vivo

**Fig. 4:**
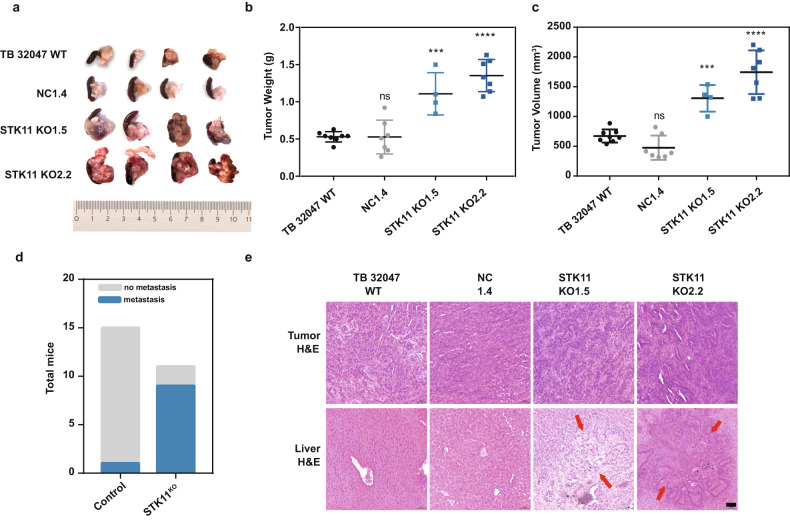
Tumor metastasis was promoted in an STK11^KO^-cell-injected orthotopic mouse model of PDAC. **a** Representative images of pancreatic tumors from the indicated group. **b**, **c** Resulting tumors were collected on day 21, measured, and weighed in comparison to the control group. Data are represented as the mean ± SD of at least four mice per group. ***p < 0.001; ****p < 0.0001 by one-way ANOVA. **d** Fisher’s exact t-test shows a significant difference in metastasis formation with STK11^KO^ cells (*p* < 0.001). **e** H&E staining was used to confirm the formation of metastases. Representative livers with metastases are shown. The arrows point to cancer cells in a metastatic lesion that surrounds normal liver tissue. Scale: 50 μm.

An orthotopic pancreatic cancer model was used to investigate the effects of STK11 on the formation of pancreatic tumors and liver metastases in C57/BL6 mice [21]. Loss of *STK11* significantly increased the tumor volume and weight (Fig. 4a–c). Metastases were macroscopically observed in 1 of 15 mice injected in the control group (TB 32047 WT and NC1.4) and in 9 of 11 mice injected with *STK11*^KO^ cells (TB KO1.5 and TB KO2.2) (Fig. 4d, *p* = 0.0007). The presence of metastatic tumors was confirmed by H&E staining of sectioned liver tissues obtained from the mice (Fig. 4e).

### Loss of STK11 preferentially targets blood vessel morphogenesis, while also altering the expression of PDEs in TB 32407 and PANC-1 cells

**Fig. 5:**
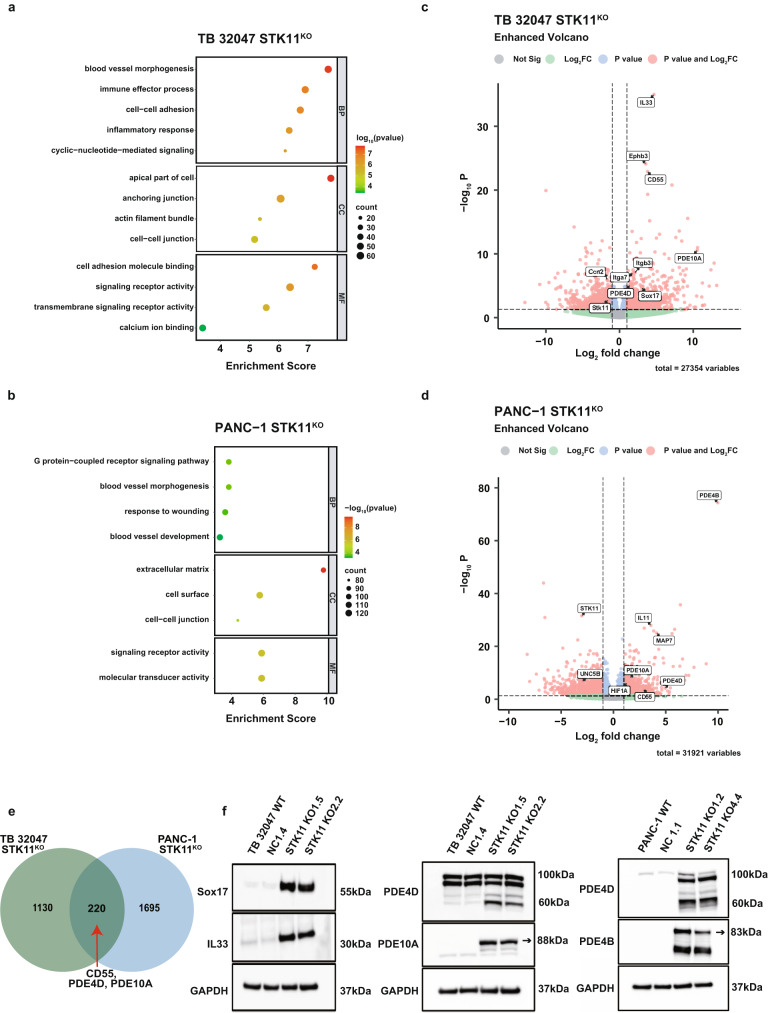
The RNA sequencing and analysis of the GO pathway revealed that the significant gene expression change was induced by loss of *STK11* in the TB 32047 and PANC-1 samples. **a**, **b** GO functional classification of the DEGs in TB 32047 and PANC-1 cells (STK11^KO^ vs. Control). The distributions are summarized in three main categories: BP, MF, and CC. The *x* axis indicates the number of genes in each category, and the y axis indicates different GO terms. **c**, **d** Volcano plot representation of the RNA-seq results showing the number of genes with significantly altered expression after STK11 knockout in TB 32047 and PANC-1 cells. In the volcano graph, non-significant genes are shown in gray, significant genes (|log_2_FC|>1) are highlighted in green, *p* < 0.05 genes are highlighted in blue, and genes with significant p-values and log_2_FC values are highlighted in red. **a**–**d** were plotted by https://www.bioinformatics.com.cn (last accessed on 31 Oct 202), an online platform for data analysis and visualization. **e** Venn diagram showing the overlap between sets of differentially expressed transcripts (|log_2_FC|>1, *p* < 0.05) in TB 32047 and PANC-1 samples. **f** Western blot analysis of Sox17, IL33, PDE4D, and PDE10A in STK11^KO^ TB 32047 and PANC-1 samples. GAPDH was used as the loading control. The observation of multiple bands in this analysis is due to the existence of various isoforms for the enzymes PDE4B, PDE4D, and PDE10A.

RNA sequencing was utilized to compare the STK11^KO^ group, encompassing three distinct knockout variants, with the control group that comprises the wild type and two different negative control cells. Principal component analysis (PCA) based on the expression of the differentially expressed genes (DEGs) confirmed that most of the variance in the data of TB 32047 cells (37%, PC1) and PANC-1 cells (43%, PC1) was associated with changes in expression between STK11^KO^ and control (WT and NC) samples (Supplementary Fig. 3a, b). All differentially expressed genes (adj *p* < 0.05) were visualized to assess sample consistency (Supplementary Fig. 3c–h). GO analysis revealed a significant enrichment of genes involved in blood vessel morphogenesis, angiogenesis, and inflammatory response in STK11^KO^ TB 32047 cells (Fig. 5a), blood vessel morphogenesis, response to wounding, and blood vessel development in STK11^KO^ PANC-1 cells (Fig. 5b).

The most upregulated genes in murine STK11^KO^ cells were SRY box transcription factor 17 (Sox17), interleukin 33 (*Il33*), and decay-accelerating factor (*Cd55*) (Fig. 5c, f and Supplementary Fig. 4a–d). Eph receptor B3 (Ephb3), cellular communication network factor 2 (Ccn2), integrin alpha 7 (Itga7), and integrin subunit beta 3 (Itgb3) are also essential genes involved in blood vessel morphogenesis and angiogenesis signaling and play a critical role in the metastasis of PDAC. Ephb3, Itga7, and Itgb3 mRNA levels increased significantly in STK11^KO^ TB 32047 cells (Supplementary Fig. 4e, f, h). STK11 expression was positively correlated with Ccn2 (Supplementary Fig. 4g). The most upregulated genes in human STK11^KO^ cells were interleukin 11 (*IL11*), microtubule-associated protein 7 (*MAP7*), and *CD55* (Fig. 5d and Supplementary Fig. 4k–n). Murine and human STK11^KO^ cells shared a significant proportion of 220 transcripts, including 42 upregulated and 78 downregulated transcripts including CD55, PDE4D, and PDE10A (Fig. 5e). Gene ontology enrichment score analysis showed that the loss of *STK11* promoted PDAC migration and metastasis through blood vessel morphogenesis and angiogenesis pathways.

Integrated analysis of the transcriptomes of TB 32047 and PANC-1 cell lines revealed STK11-driven regulation of PDEs. STK11 knockout was selectively effective in increasing the expression of PDE4D (Fig. 5f and Supplementary Fig. 4i, o). In STK11 knockout cells, the mRNA level of *PDE4D* changed more than four times, but the mRNA level of *PDE10A* changed more than a thousand times (Supplementary Fig. 4i, j). The basal expression of PDE10A in TB 32047 cells was below the detection limit and STK11 deletion may activate PDE10A transcription via an unknown mechanism. However, PDE4B expression was not detected in WT or STK11^KO^ TB 32047 cells. In PANC-1 cells, the expression of PDE4B in STK11^KO^ cells at the mRNA and protein levels increased significantly, whereas PDE10A expression did not change significantly (Supplementary Fig. 4p, q).

Next, the above-mentioned targets were validated in additional PDAC cell lines (Supplementary Fig. 5). The expression levels of *Sox17, IL33, CD55*, and *PDE4D* were also significantly elevated after STK11 knockout in KPC 792 cell line (Supplementary Fig. 5a–e, o). PDE4B, but not MAP7 or CD55, was highly expressed in STK11-deleted SUIT2 cells (Supplementary Fig. 5f–i, p). PDE4D, PDE4B, PDE10A, and CD55 were highly expressed in STK11-deleted SU86.86 cells (Supplementary Fig. 5j–n, q). These results indicate that PDEs are the most promising targets among several STK11 mutant PDAC cell lines.

### *STK11*^*KO*^ cell migration regulation by PDE inhibitors in PDAC

**Fig. 6:**
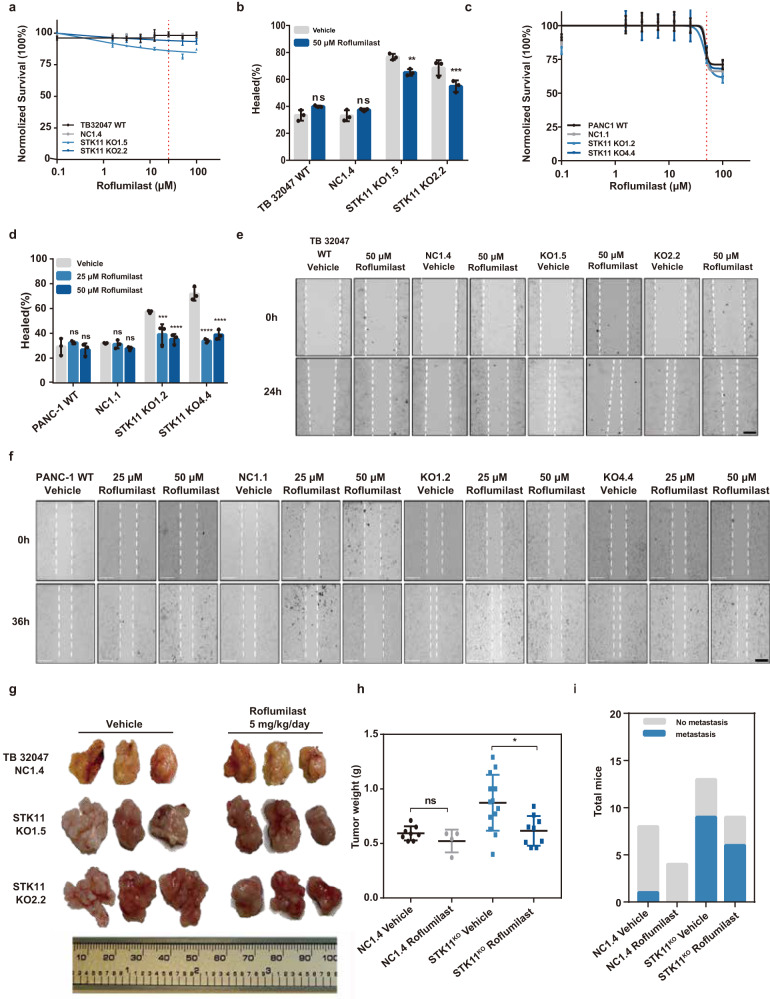
Roflumilast attenuated STK11-mutated migration in vitro and tumor progression in vivo. **a**, **c** TB 32047 and PANC-1 cells were exposed to increasing doses of Roflumilast. The percentage of cell viability relative to that of cells is shown. Data represent the mean ± SD of three replicates. **b** Quantitative analysis of TB 32047 cells that migrated with or without Roflumilast after 24 h. Data are presented as means of three independent experiments. **p < 0.01; ***p < 0.001 by one-way ANOVA. Images taken at 0 h and 24 h are shown in **e**. **d** Quantitative analysis of PANC-1 cells that migrated with or without Roflumilast after 36 h. Data are presented as means of three independent experiments. ***p < 0.001; ****p < 0.0001 by one-way ANOVA. Images taken at 0 h and 36 h are shown in **f**. **g** Representative images of pancreatic tumors from the indicated group. **h** The resulting tumors were collected on day 23 (day 8–23 with Roflumilast), weighed, and compared to the vehicle group. Data are presented as the mean ± SD of at least four mice per group. ns, *p* = 0.1767; *p < 0.05, by unpaired t-test. **i** Fisher’s exact t-test shows the difference in the formation of metastases with Roflumilast treatment mice. ns, *p* = 1 (NC1.4 Vehicle vs. NC1.4 Roflumilast); ns, *p* = 1 (STK11^KO^ Vehicle vs. STK11^KO^ Roflumilast).

The viability of WT and STK11^KO^ cells was not affected by the PDE4 inhibitors (Fig. 6a, c and Supplementary Fig. 6c). However, cell migration decreased significantly in STK11^KO^ cells but not in WT TB 32047 cells after treatment with Roflumilast (50 μM) (Fig. 6b, e). In PANC-1 cells, after treatment with Rolipram and Roflumilast, cell migration was significantly decreased in STK11^KO^ cell lines, whereas not in PANC-1 WT cells (Fig. 6d, f and Supplementary Fig. 6d). Because of the high expression of PDE10A in STK11^KO^ TB 32047 and SU86.86 cell lines, Mardepodect (a PDE10A inhibitor) was used (Supplementary Fig. 6a, b, i, j). In the TB 32047 and SU86.86 cell lines, Mardepodect not only reduced the migratory ability of STK11^KO^ cells, and showed more toxic to STK11^KO^ cells than WT and NC. The PDE4 inhibitor also exerted an inhibitory effect on the migration of STK11^KO^ SUIT2 and SU86.86 cells (Supplementary Fig. 6e–h, j–l). This suggested that PDEs play an essential role in the migration of STK11^KO^ PDAC cells.

Based on these observations, we examined the effects of Roflumilast in C57BL/6 mice. Relative pancreatic weight and representative images of H&E staining of pancreatic tissues showed that Roflumilast hampered PDAC progression in the STK11^KO^ group (Fig. 6g, h and Supplementary Fig. 7a, c), although liver metastasis was not significantly reduced (Fig. 6i and Supplementary Fig. 7b).

## Discussion

Large-scale CRISPR/Cas9 screening efforts to inhibit gene expression have revolutionized genetic screening, enabling previously impossible discoveries [22]. Herein, we present a protein-kinase-wide screening method that reveals known and novel mechanisms of metastasis in PDAC. A lentivirus-based sgRNA library targeting mouse protein kinase was used to analyze protein-kinase-wide loss-of-function by stable gene knockout. Although screening for cell migration regulators by Transwell compartments has previously been reported [23], in this study, we used CRISPR/Cas9 technology instead of RNAi-based functional selection. Several cell migration-regulating genes were identified in the present study have been reported previously, thus validating the selection approach. For example, Prkar1a knockout in the pancreas leads to neuroendocrine tumorigenesis. Δ-Prkar1a mice showed local invasion, neoplastic emboli in the lymphatic vessels, and occasional metastases to the locoregional lymph nodes in histological analyses [24]. Several studies have highlighted a possible involvement of the STK11 pathway in metastasis. In lung cancer, STK11 knockdown has been reported to increase cell motility and invasiveness and influence many epithelial–mesenchymal transition (EMT) pathway marker proteins, such as ZEB1 and E-cadherin [25]. Vimentin, a marker of the EMT pathway, was analyzed in STK11^KO^ cells (Supplementary Fig. 8) and its expression was found to be significantly higher in STK11-deficient PANC-1 and SUIT2 cells than in the WT and NC groups. Moreover, STK11 deficient cells have been reported to exhibit impaired DNA damage [26]. Consistent with this information, the absence of STK11 enhanced cell sensitivity to IR and decreased colony formation and cell survival compared with wild-type STK11 cells (Supplementary Fig. 9). Therefore, STK11 mutation might be a potential biomarker for the selection of patients for radiation therapy.

In lung cancer, STK11 loss causes a metastasis-like subpopulation of cancer cells in primary tumors and metastases to activate the early endodermal transcription factor, Sox17. In *STK11*-deficient cells, Sox17 expression is essential and sufficient to trigger a second wave of epigenetic alterations that improve metastatic potential [27], which is consistent with the elevation of Sox17 expression in STK11^KO^ PDAC mouse cell lines (Supplementary Figs. 4b and 5b, o). However, this correlation has only been observed in mouse PDAC cell lines, with inconsistent results in human PDAC cell lines. The same applies to the effect of STK11 deletion on IL33 (Supplementary Figs. 4c and 5c, o). The deletion of *STK11* significantly increased *IL33* mRNA and protein levels in mouse PDAC cell lines. However, in the PANC-1 cell line, the mRNA level of *IL11* not *IL33* increased in STK11^KO^ (Supplementary Fig. 4n). The difficulty in targeted STK11 therapy is well illustrated by the specificity of the downstream targets of STK11 in different cell lines. The different genetic backgrounds, gene expression profiles, and mutational profiles of different cell lines add to the complexity of this study.

PDE4D, PDE4B, and PDE10A are the PDEs. PDE enzymes with 11 isoforms catalyze the hydrolysis of cyclic nucleotides cyclic adenosine monophosphate (cAMP) and cyclic guanosine monophosphate (cGMP). PDE4 was the largest and one of the first discovered PDE families. It contains four genes (PDE4A, PDE4B, PDE4C, and PDE4D) and is a cAMP-specific phosphodiesterase [28]. Among several STK11-specific signatures, PDE4B, PDE4D, and PDE10A were upregulated, depending on whether the *STK11* gene was a mutant. A significant increase in PDE10A caused by STK11 knockout was observed in TB 32047 and SU86.86 cell lines, a significant increase in PDE4B caused by STK11 knockout was observed in three human PDAC cell lines, and a significant increase in PDE4D caused by STK11 knockout was observed in two mouse and two human PDAC cell lines. Owing to the variability in different cell lines, STK11 knockout regulates different PDE isoforms in different PDAC cell lines, which may be related to the basal expression of PDEs in different cell lines.

Understanding the complicated mechanisms that drive the spread of cancer will provide crucial information for therapeutic interventions. Recently, an association between PDE4 inhibitors and cancer therapy was reported. PDE4 inhibitors can overcome tumor resistance and decrease human glioblastoma cell survival [29] and lung cancer. PDE4-specific inhibitors inhibit proliferation and growth of STK11-mutated cells [30]. Roflumilast was the first PDE4 inhibitor approved by the FDA for the prevention of chronic obstructive pulmonary disease (COPD) exacerbations [31] and effectively inhibits tumor proliferation and growth in ovarian cancer [32]. Roflumilast may be related to the prevention of enhanced cAMP-mediated inflammatory processes and angiogenesis indicators in tumor tissues [19]. In this study, because of the high expression of PDE4B and PDE4D in *STK11*-mutated PDAC cells, two different PDE4 inhibitors, Rolipram and Roflumilast, were used to treat the cells. After treatment, cell migration decreased significantly in the three *STK11* mutant human PDAC cell lines, and no significant decrease in cell migration was observed compared with most vehicle controls. Mardepodect, a specific PDE10A inhibitor, significantly decreased cell migration in *STK11* mutant TB 32047 and SU86.86 cell lines. In mice with STK11 mutant tumors, Roflumilast treatment reduced the number of liver metastases; however, a significant reduction was not achieved compared to that in the non-treated group. However, there was a significant reduction in the tumor weight in the Roflumilast-treated group (Fig. 6g, h). Considering the better metastasis suppression effect of Roflumilast on STK11-mutated human-derived PDAC cell lines than on mouse-derived PDAC cell lines in vitro (Fig. 6b, d and Supplementary Fig. 6g, j), maybe better results can obtain in NSG mice (NOD scid gamma mouse). Recent research has discovered that targeting PDE4D can inhibit the development of pancreatic cancer tumors in NOD mouse models, further confirming our hypothesis [33].

Although more mechanistic studies are required to understand the relationship between STK11 and PDEs in the progression of PDAC, the results reported herein also indicate a potential path for therapeutic intervention with Roflumilast for STK11 mutant pancreatic cancer patients. The pharmacological profile of Roflumilast makes it a potential targeted drug with promising clinical applications.

## Materials and methods

### Cell culture

PANC-1 (CRL-1469^TM^, RRID: CVCL_0480) cell line was purchased from ATCC® (American Type Culture Collection) and grown in Roswell Park Memorial Institute Medium 1640 supplemented with 10% (v/v) fetal bovine serum (FBS). SUIT2 cell line was purchased from the Japanese Collection of Research Bioresource Cell Bank (JCRB1094, RRID: CVCL_3172) and grown in a minimum essential medium supplemented with 10% (v/v) FBS. SU86.86 (CRL-1837^TM^, RRID: CVCL_3881), Miapaca2 (RM-CRL-1420^TM^, RRID: CVCL_0428), Aspc1 (CRL-1682^TM^, RRID: CVCL_0152), and Panc 05.04 (CRL-2557^TM^, RRID: CVCL_1637) cell lines were obtained from ATCC®. The TKCC-10 [34] and Mayo4636 [35] cell lines were gifts from David Chang and Debabrata Mukhopadhyay, respectively. TB 32047 [36], obtained courtesy of Prof. David Tuveson, Cold Spring Harbor Laboratory, was grown in Dulbecco’s modified Eagle’s medium with 10% (v/v) FBS. KPC 792 [37], was obtained from Marc P. Stemmler. HEK293TN cells (CRL-3216^TM^, RRID: CVCL_UL49), also obtained from ATCC®, were grown in DMEM supplemented with 10% of inactivated FBS. Cells were maintained at 37 °C in a 5% CO_2_. Additional information regarding the cell culture media is provided in Supplementary Table 1. All the media were free of penicillin/streptomycin. DNA fingerprinting using highly polymorphic short tandem repeat (STR) analysis was used to authenticate the cells, and mycoplasma testing was performed regularly to ensure that the cells were free of mycoplasma contamination.

### Generation of the CRISPR/Cas9 lentiviral library and screening

The mouse ACF CRISPR knockout library included a pooled mouse Brie kinome library, which was a gift from John Doench and David Root (Addgene plasmid #75316), and additional sgRNAs designed for genes involved in pancreatic ductal adenocarcinoma (PDAC). The library contains 3466 unique sgRNAs targeting 915 genes. ACF CRISPR knockout library virus particles were generated in T75 flasks. HEK293TN cells were co-transfected with the 4.3 µg sgRNA library plasmid, 2.8 µg pMDLg/pRRE (Addgene plasmid #12251), 1.4 µg pRSV-REV (Addgene plasmid #12253), and 1.4 µg pMD2.G (Addgene plasmid #12259) using Lipofectamine 3000 transfection reagent, and viral supernatants were collected after 24 h. TB 32047 Cas9 cells were transduced with the virus at an MOI of 0.3. After a few days of steady growth, 450,000 library cells resuspended in serum-free medium were divided and placed on three Transwell FluoroBlok culture inserts (Corning, #351152, 8.0 μm), and the lower Transwell chambers were filled with medium containing 10% FBS. After 6 h of incubation, cells that migrated to the lower faces of the inserts were collected by trypsinization. To identify integrated sgRNAs in the migrated cells, 10 million cells were collected for genomic DNA isolation.

### RNA sequencing and Gene Ontology enrichment analysis

Total RNA was extracted from the TB 32047 and PANC-1 cells. RNA quality check and then libraries were pooled and sequenced on an Illumina Hiseq 2500 platform. The reads were aligned to the GRCm38.6 mouse reference genome or the GRCh38.6 human reference genome using STAR aligner (RRID: SCR_004463, version 2.7.8. a). Unique mappings were counted using featureCounts (version 2.0.1) when overlapping exons were from the Ensembl gene model (*Mus musculus*, version 102). Counting of reads per gene also uses strand information about the library molecule alignments to separately count overlapping genes with different strands. Normalized read counts were used for downstream analysis. Statistical analysis to identify differentially expressed genes between the compared groups was performed using the DESeq2 package (RRID: SCR_000154, version 1.30) in the R statistical environment (version 4.0.3) using default parameters. Heat maps were used to visualize the results. Gene ontology (GO) describes gene products with three independent categories: biological processes, cellular components, and molecular functions. GO was determined using R package clusterProfiler (RRID: SCR_016884, version 3.16.1). Differentially expressed protein-coding genes (|log_2_(Fold Change)| >1) were included, and *p* < 0.05 was considered significant for the enrichment of biological processes.

### CAM assay

Fertilized and pathogen-free eggs (VALO BioMedia) were used in the CAM assay. The eggs were opened on the flattened pole on day 8 of embryonic development and the eggshell membrane was removed. On day 9, the TB 32047 cells were resuspended in a Matrigel–medium (1:1) mixture. Pipetted drops of the Matrigel/cell solution were placed in sterile culture dishes. One Matrigel/cell pellet was carefully placed in the CAM of each egg. The eggs were then incubated for another five days. The samples were further incubated at RT in 4% formalin solution more than 24 h and processed to create formalin-fixed, paraffin-embedded (FFPE) blocks. FFPE sections were stained with hematoxylin and eosin (H&E) and pan-cytokeratin. Five randomly selected high-power fields of each sample were used to analyze the characteristics of the morphological cells. The sample volume was calculated using the following equation: Volume = length×width×height×0.52 [38].

### Genomic DNA isolation and PCR amplification

Genomic DNA was extracted using NucleoSpin® Blood XL (Machery Nagel, #740950.50), according to the manufacturer’s protocol. Next, 10 μg of genomic DNA isolated from the migrated and control cells (mouse ACF CRISPR knockout library cells) was subjected to PCR. sgRNA segments were amplified as described previously [39]. The PCR reactions (100 μL) included 10 μg of DNA, 50 μL of Q5® Hot Start High-Fidelity 2X Master Mix (NEB, #M0494L), 3 μL of a 10 μM solution of P5 (forward primer: ACACTCTTTCCCTACACGACGCTCTTCCGATCTNNNNNTCTTGTGGAAAGGACGAAACACCG), and 3 μL of a 10 μM solution of P7 (reverse primer: GTGACTGGAGTTCAGACGTGTGCTCTTCCGATCTTCTACTATTCTTTCCCCTGCACTGT). The diluted PCR product was prepared on an Illumina Hiseq 2500 platform at the Technische Universität Dresden (TU Dresden) deep sequencing facility. Raw FASTQ files were analyzed using PinAPL-Py software [40].

### Construction of stable knockout cell lines by CRISPR/Cas9

STK11 knockout cell lines were generated using the SpCas9 (BB)-2A-Puro vector (Addgene plasmid #48139), which includes the Cas9 protein. First, two good sgRNAs targeting STK11 were selected from screening data for synthetic oligos. sgRNA oligos in SpCas9 (BB)-2A-Puro were used to generate the pSpCas9(BB)-2A-Puro-STK11. pSpCas9 (BB)-2A-Puro-STK11-specific plasmids were transfected into TB 32047, KPC 792, PANC-1, SUIT2, or SU86.86 cells using Lipofectamine 3,000 reagent according to the manufacturer’s protocol. After 24 h of transfection, cells were selected using 10 µg/ml puromycin for 3 days. Oligonucleotides targeting STK11 are listed in Supplementary Table 2. The number of transfected cells was counted and 80 cells were diluted in 12 ml of medium in each 96-well plate. The correct single-clone cells were selected under a microscope after 5–10 day. The selected clones were verified using RT-qPCR, western blotting, and Sanger sequencing.

### Wound-healing assay

An appropriate volume of working cell solution was prepared at a density of 3–5 × 10^5^ cells/ml and seeded at a volume of 100 μl/well in 96-well plates, 500 μl/well in 24-well plates, and 1 ml/well in 12-well plates. When the cells reached 98% confluence, the medium was exchanged with free serum medium (TB 32047 or SU86.86 cell line), 1–2% serum medium (PANC-1, KPC 792, or SUIT2 cell line), or PDE inhibitors for 6–36 h for starvation or inhibition, as described previously [41]. A wound was then created using a vacuum pump, wound-maker, or pipette tip. Any remaining medium was removed from the edges of the wells and the cells were washed with PBS. PBS was then replaced with free FBS medium (TB 32047 or SU86.86 cell line), 1–2% serum medium (PANC-1, KPC 792, or SUIT2 cell line), or medium with inhibitor for 24—36 h. Using EVOS microscopy, images of each complete wound were captured centered in the field of view.

### RNA extraction and RT-qPCR analysis

Total RNA was extracted from the cell lines using the NucleoSpin® RNA Plus kit (MACHEREY-NAGEL, #740984.250). cDNA was synthesized using a high-capacity cDNA reverse transcription kit (Applied BiosystemsTM, #4368814). GAPDH or β-actin was used as an internal control to quantify the mRNA levels of other genes. The sequences of gene-specific primers are listed in Supplementary Table 3. Relative mRNA levels were determined by RT-qPCR on a Light Cycler 480 II using the SYBR Green method (Applied BiosystemsTM, #4367659). Three biological replicates were used for each experiment. The relative mRNA expression levels were calculated using the 2 − ∆∆Ct method.

### Establishment of the PDAC 3D organoid model

A total of 5000 cells were resuspended in 50 μl Matrigel, seeded in 6-well plates as a dome, and solidified at 37 °C for 30 min. A maximum of six domes was plated in each well. A feeding medium was added, and the cells were incubated at 37 °C for 7–10 days. After sphere formation, the supernatant was discarded. The domes were collected using a cell recovery medium (Corning, # 342053) and placed on ice for 30 min. Enzyme digestion was then performed with a mixture of organoid digestion medium (Supplementary Table 1; 1 ml per six domes). The domes were then incubated for 5 min at 37 °C and centrifuged at 250 ×g at 4 °C. Next, 2 ml of washing medium was rinsed over the cell pellets to digest as many spheroids as possible. The cells were counted using an automated cell counter and centrifuged at 250 ×g for 5 min at 4 °C. The supernatant was removed and 40,000 cells were resuspended in 20 μl phenol-red-free Matrigel (Corning, #356231).

### Sanger sequencing

Sanger sequencing can be used to detect the targeted genomic modifications. Genomic DNA was extracted using NucleoSpin® tissue (Machery Nagel, #740952.50) according to the manufacturer’s protocol for cultured cells. Two oligos were designed to target the modified regions using Primer Premier 5. The primers designed for the knockout check are listed in Supplementary Table 4. Amplicons were subcloned into a plasmid using the NEB® PCR Cloning Kit (NEB, #E1203S) for transformation and several individual colonies were selected and sequenced to determine the clonal genotype (Supplementary Table 5).

### X-irradiation

cells were divided into 100, 200, 300, 800, 1500, 2500, and 5000 per well. After 24 h, cells were separately exposed to 0, 1, 2, 4, 6, 8, or 10 Gy X-rays using a Siemens Primus Accelerator machine (6 Mv; Siemens AG, Munich, Germany) and cultured for 7–12 days. The colonies were fixed with methanol and stained with 0.5% crystal violet. More than 50 cells were counted as a colony. The efficiency of clone formation was calculated and a survival curve was generated by comparison with the untreated group.

### Immunofluorescence imaging

Cells were grown in 4-well chamber slides, fixed with 4% paraformaldehyde, and incubated overnight with primary antibodies or Isotype Normal Rabbit IgG (R&D System, AB-105-C). Proteins were visualized by incubation with Alexa FlourTM 488 goat anti-rabbit IgG (Invitrogen, #A11034; Invitrogen). Nuclei were visualized by incubation with DAPI (1:5000). The coverslips or slides were covered with a mounting medium (Dako, #S302380). Fluorescence was monitored with a Leica TCS SP8 confocal laser microscope (Manheim, Germany) and processed using Leica Application Suite X v2.0.1.14392. The antibodies used are listed in Supplementary Table 6.

### Western blot

Cells were lysed using radioimmunoprecipitation assay buffer (RIPA) and phosphatase inhibitor cocktail (1:100). Protein concentration was determined photometrically using a Pierce® BCA Protein Assay Kit (Thermo Fisher, #23225). Gel electrophoresis was performed using Bolt^TM^ 4–12% Bis-Tris Plus gels (Thermo Fisher, #NW04122BOX), and the proteins were transferred to a nitrocellulose membrane. The membrane was incubated in a blocking solution for 1 h at room temperature. The primary antibody was diluted with 5% milk or bovine serum albumin (BSA), and the membrane was incubated in the primary antibody solution overnight at 4 °C with gentle rocking. The membrane was further incubated with 5% milk-diluted secondary antibodies for 1 h at room temperature, with gentle rocking (Supplementary Table 6). Signals were detected using an Amersham^TM^ Imager 600 with Signal Fire^TM^ (Elite) ECL reagent (Cell Signaling Technology, #6883 or #12757). All western blotting assays were performed at least thrice to validate the reliability of the results.

### Establishment of an orthotopic murine pancreatic cancer model and Roflumilast treatment

All animal studies met the relevant ethical considerations and were monitored according to the German legislation on animal protection and the Guide for the Care and Use of Laboratory Animals [42]. C57BL/6JRj (Black 6) mice, without gender selection and aged 7 weeks, were acclimated to the animal facility for one week. Animals were randomly divided into groups (*n* = 5 per group). The pancreas was extracted and held in the tail to extend it, and then 4×10^4^ spheres resuspended in 20 μl Matrigel were injected directly into the body of the pancreas [43], superior to the visible large blood vessels. After three weeks, the pancreas and livers were excised for further pathological examination.

In all, 4 × 10^4^ spheres mixed with 20 μl Matrigel were injected directly into the body of the pancreas for treatment with Roflumilast (Hycultec, #HY-15455). After one week, mice were treated intraperitoneally with Roflumilast (5 mg/kg/day) or vehicle as indicated. After 16 days of treatment, the mice were killed and the pancreas and livers were collected for further evaluation. Tumor volume was measured with calipers and calculated using the following formula: volume = length × width × height × 0.52 [38].

### Statistical analysis

Statistical tests were performed using GraphPad software (RRID:SCR_002798, v.7) to compare groups under different conditions with replicates. In all tests, statistical significance was established as *p < 0.05, **p < 0.01, ***p < 0.001, and ****p < 0.0001. T-test and one-way and two-way ANOVAs were used for cell culture experiments. Fisher’s exact test was used for mouse experiments.

### Supplementary information

Suplementary information

Supplemental Material_Western Blot

Supplemental table of full sgRNA screening results

Supplemental table list of sgRNAs in screeing

## Data Availability

Raw sequencing files were deposited at the SRA under accession number PRJNA960707. All other data are available in the article and Supplementary Information. Source data were provided in this study.

## References

[1] Sung H, Ferlay J, Siegel RL, Laversanne M, Soerjomataram I, Jemal A (2021). Global Cancer Statistics 2020: GLOBOCAN Estimates of Incidence and Mortality Worldwide for 36 Cancers in 185 Countries. CA: A Cancer J Clin.

[2] Liu M, Wang M, Li S (2021). Prognostic factors of survival in pancreatic cancer metastasis to liver at different ages of diagnosis: a SEER Population-Based Cohort Study. Front Big Data.

[3] Zhang Y, Meng Q, Sun Q, Xu Z-X, Zhou H, Wang Y (2020). LKB1 deficiency-induced metabolic reprogramming in tumorigenesis and non-neoplastic diseases. Mol Metab.

[4] Cotton JL, Dang K, Hu L, Sun Y, Singh A, Rajurkar MS (2022). PTEN and LKB1 are differentially required in Gli1-expressing mesenchymal cells to suppress gastrointestinal polyposis. Cell Rep.

[5] Choi SH, Do SK, Lee SY, Choi JE, Kang H-G, Hong MJ (2022). Genetic variants in LKB1/AMPK/mTOR pathway are associated with clinical outcomes of chemotherapy in non-small cell lung cancer. Thorac Cancer.

[6] Long L-L, Ma S-C, Guo Z-Q, Zhang Y-P, Fan Z, Liu L-J (2023). PARP inhibition induces synthetic lethality and adaptive immunity in LKB1-mutant lung cancer. Cancer Res.

[7] Rosellini P, Amintas S, Caumont C, Veillon R, Galland-Girodet S, Cuguillière A (2022). Clinical impact of STK11 mutation in advanced-stage non-small cell lung cancer. Eur J Cancer.

[8] Song L, Guo J, Chang R, Peng X, Li J, Xu X (2018). LKB1 obliterates Snail stability and inhibits pancreatic cancer metastasis in response to metformin treatment. Cancer Sci.

[9] Rho SB, Byun HJ, Kim B-R, Lee CH (2021). Knockdown of LKB1 sensitizes endometrial cancer cells via AMPK activation. biomolecules & therapeutics. Biomol Ther.

[10] Cui X, Wang X, Zhou X, Jia J, Chen H, Zhao W (2020). miR-106a regulates cell proliferation and autophagy by targeting LKB1 in HPV-16-associated cervical cancer. Mol Cancer Res.

[11] Dzung A, Saltari A, Tiso N, Lyck R, Dummer R, Levesque MP (2022). STK11 prevents invasion through signal transducer and activator of transcription 3/5 and FAK repression in cutaneous melanoma. J Investig Dermatol.

[12] Gupta M, Sherrow C, Krone ME, Blais EM, Pishvaian MJ, Petricoin EF (2021). Targeting the NTRK fusion gene in pancreatic acinar cell carcinoma: a case report and review of the literature. J Natl Compr Cancer Netw.

[13] Chi J, Chung SY, Parakrama R, Fayyaz F, Jose J, Saif MW (2021). The role of PARP inhibitors in BRCA mutated pancreatic cancer. Ther Adv Gastroenterol.

[14] Kasuga A, Okamoto T, Udagawa S, Mori C, Mie T, Furukawa T (2022). Molecular features and clinical management of hereditary pancreatic cancer syndromes and familial pancreatic cancer. Int J Mol Sci.

[15] Amato E, Molin MD, Mafficini A, Yu J, Malleo G, Rusev B (2014). Targeted next-generation sequencing of cancer genes dissects the molecular profiles of intraductal papillary neoplasms of the pancreas. J Pathol.

[16] Sato N, Rosty C, Jansen M, Fukushima N, Ueki T, Yeo CJ (2001). STK11LKB1 Peutz-Jeghers gene inactivation in intraductal papillary-mucinous neoplasms of the pancreas. Am J Pathol.

[17] Su GloriaH, Hruban RalphH, Bansal RaviK, Bova GSteven, Tang DavidJ, Shekher ManuC (1999). Germline and somatic mutations of the STK11LKB1 Peutz-Jeghers gene in pancreatic and biliary cancers. Am J Pathol.

[18] Li H, Zuo J, Tang W (2018). Phosphodiesterase-4 inhibitors for the treatment of inflammatory diseases. Front Pharmacol.

[19] Yeo CD, Kim YA, Lee HY, Kim JW, Kim SJ, Lee SH (2017). Roflumilast treatment inhibits lung carcinogenesis in benzo(a)pyrene-induced murine lung cancer model. Eur J Pharmacol.

[20] Kelly K, Mejia A, Suhasini AN, Lin AP, Kuhn J, Karnad AB (2017). Safety and pharmacodynamics of the PDE4 inhibitor roflumilast in advanced B-cell malignancies. Clin. Cancer Res.

[21] Vudatha V, Herremans KM, Freudenberger DC, Liu C, Trevino JG (2023). In vivo models of pancreatic ductal adenocarcinoma. Adv. Cancer Res.

[22] Henkel L, Rauscher B, Schmitt B, Winter J, Boutros M (2020). Genome-scale CRISPR screening at high sensitivity with an empirically designed sgRNA library. BMC Biol.

[23] Seo M, Lee S, Kim J-H, Lee W-H, Hu G, Elledge SJ (2014). RNAi-based functional selection identifies novel cell migration determinants dependent on PI3K and AKT pathways. Nat Commun.

[24] Saloustros E, Salpea P, Starost M, Liu S, Faucz FR, London E (2017). Prkar1a gene knockout in the pancreas leads to neuroendocrine tumorigenesis. Endocr Relat Cancer.

[25] Roy BC, Kohno T, Iwakawa R, Moriguchi T, Kiyono T, Morishita K (2010). Involvement of LKB1 in epithelial-mesenchymal transition (EMT) of human lung cancer cells. Lung Cancer.

[26] Wang Y-S, Chen J, Cui F, Wang H, Wang S, Hang W (2016). LKB1 is a DNA damage response protein that regulates cellular sensitivity to PARP inhibitors. Oncotarget.

[27] Pierce SE, Granja JM, Corces MR, Brady JJ, Tsai MK, Pierce AB (2021). LKB1 inactivation modulates chromatin accessibility to drive metastatic progression. Nat Cell Biol.

[28] Boyd A, Aragon IV, Abou Saleh L, Southers D, Richter W (2021). The cAMP-phosphodiesterase 4 (PDE4) controls β-adrenoceptor- and CFTR-dependent saliva secretion in mice. Biochem J.

[29] Peng T, Gong J, Jin Y, Zhou Y, Tong R, Wei X (2018). Inhibitors of phosphodiesterase as cancer therapeutics. Eur J Med Chem.

[30] He N, Kim N, Song M, Park C, Kim S, Park EY (2014). Integrated analysis of transcriptomes of cancer cell lines and patient samples reveals STK11/LKB1–driven regulation of cAMP phosphodiesterase-4D. Mol Cancer Ther.

[31] Baye J (2012). Roflumilast (Daliresp). Pharm Ther.

[32] Gong S, Chen Y, Meng F, Zhang Y, Wu H, Wu F (2017). Roflumilast restores cAMP/PKA/CREB signaling axis for FtMt-mediated tumor inhibition of ovarian cancer. Oncotarget.

[33] Jeong MH, Urquhart G, Lewis C, Chi Z, Jewell JL (2023). Inhibition of phosphodiesterase 4D suppresses mTORC1 signaling and pancreatic cancer growth. JCI Insight.

[34] Brunton H, Caligiuri G, Cunningham R, Upstill-Goddard R, Bailey U-M, Garner IM (2020). HNF4A and GATA6 loss reveals therapeutically actionable subtypes in pancreatic cancer. Cell Rep.

[35] Schwappacher R, Dieterich W, Reljic D, Pilarsky C, Mukhopadhyay D, Chang DK (2021). Muscle-derived cytokines reduce growth, viability and migratory activity of pancreatic cancer cells. Cancers.

[36] Lu S, Zhang Z, Du P, Chard LS, Yan W, El Khouri M (2020). A virus-infected, reprogrammed somatic cell–derived tumor cell (VIReST) vaccination regime can prevent initiation and progression of pancreatic cancer. Clin Cancer Res.

[37] Krebs AM, Mitschke J, Lasierra Losada M, Schmalhofer O, Boerries M, Busch H (2017). The EMT-activator Zeb1 is a key factor for cell plasticity and promotes metastasis in pancreatic cancer. Nat Cell Biol.

[38] Feldman JP, Goldwasser R, Mark S, Schwartz J (2008). A mathematical model for tumor volume evaluation using two dimensions. J Appl Quant Methods.

[39] Ran FA, Hsu PD, Wright J, Agarwala V, Scott DA, Zhang F (2013). Genome engineering using the CRISPR-Cas9 system. Nat Protoc.

[40] Spahn PN, Bath T, Weiss RJ, Kim J, Esko JD, Lewis NE (2017). PinAPL-Py: a comprehensive web-application for the analysis of CRISPR/Cas9 screens. Sci Rep.

[41] De Ieso ML, Pei JV (2018). An accurate and cost-effective alternative method for measuring cell migration with the circular wound closure assay. Biosci Rep.

[42] Council NR Guide for the Care and Use of Laboratory Animals. National Academies Press (US); 1996. 25121211

[43] Erstad DJ, Sojoodi M, Taylor MS, Ghoshal S, Razavi AA, Graham-O’Regan KA (2018). Orthotopic and heterotopic murine models of pancreatic cancer and their different responses to FOLFIRINOX chemotherapy. Dis Models Mech.

