# Supplementary material for: Roflumilast inhibits tumor growth and migration in STK11/LKB1 deficient pancreatic cancer
Source: Cell Death Discov. 2024 Mar 9;10:124. doi: 10.1038/s41420-024-01890-y (PMC10924943; doi:10.1038/s41420-024-01890-y)
Supplement: Supplementary file 1 — Suplementary information [file 41420_2024_1890_MOESM1_ESM.docx]

**SUPPLEMENTARY INFORMATION**

**Roflumilast inhibits tumor growth and migration in STK11/LKB1 deficient pancreatic cancer**

Shuman Zhang^1^, Duo Yun^1^, Hao Yang^2^, Markus Eckstein^3^, Gihan Daw Elbait^4^, Yaxing Zhou^1^, Yanxi Lu^1^, Hai Yang^1^, Jinping Zhang^1^, Isabella Dörflein^1^, Nathalie Britzen-Laurent^1^, Susanne Pfeffer^1^, Marc P Stemmler^5^, Andreas Dahl^6^, Debabrata Mukhopadhyay^7^, David Chang^8,9^, Hang He^10^, Siyuan Zeng^11^, Bin Lan^12^, Benjamin Frey^13^, Chuanpit Hampel^2^, Eva Lentsch^1^, Paradesi Naidu Gollavilli^14^, Christian Büttner^15^, Arif B. Ekici^15^, Andrew Biankin^8,9^, Regine Schneider-Stock^2^, Paolo Ceppi^14^, Robert Grützmann^1^, Christian Pilarsky^1*^


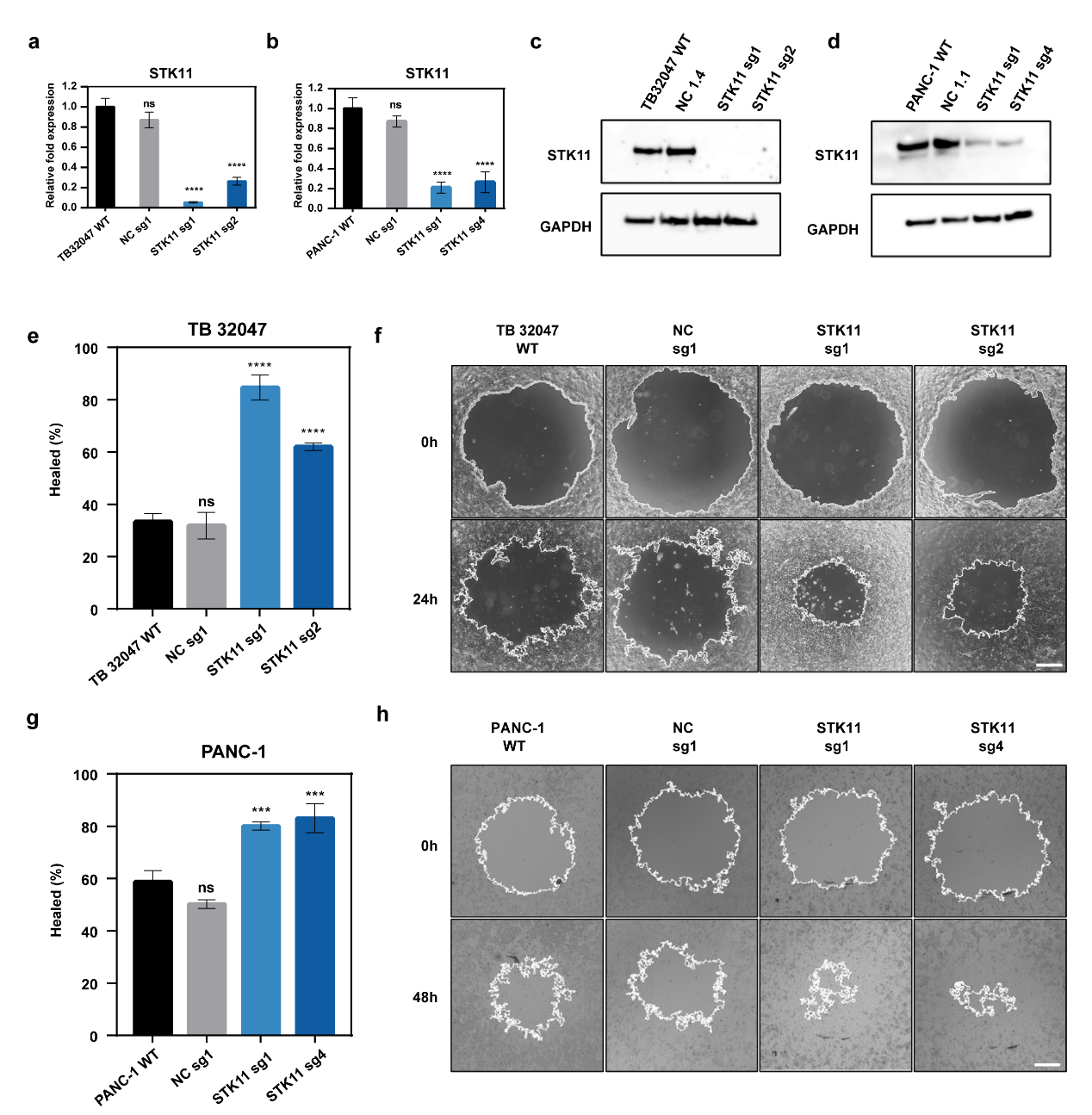


**Supplementary Fig. 1: Validation of the gene targets found by functional screening. a, b** *STK11* mRNA level was examined in TB 32047 and PANC-1 cells. Data are presented as means of three independent experiments. ****, p< 0.0001 by one-way ANOVA. **c, d** STK11^KD^ TB 32047 and PANC-1 cells were generated using CRISPR/Cas9 technology. Western blot determined STK11 expression in WT and TB 32047 and PANC-1 knockdown cells. Knockdown pool cells got from two different sgRNAs. **e, g** Scratch wound assay created a cell-free zone (wound) in a confluent cell monolayer. The relative wound area was measured after 24 or 48 h. Average ± SEM (n = 3) is shown. **f, h** Representative pictures of the wound healing assay of TB 32047 and PANC-1 cell line. Scale bar: 200 µm.

**
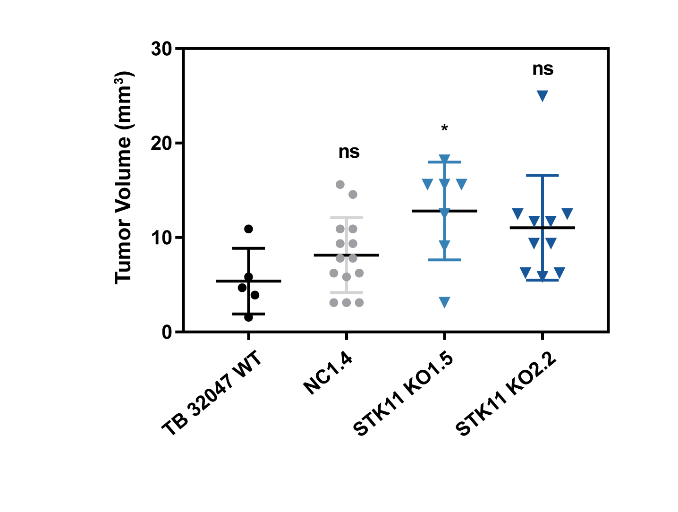
**

**Supplementary Fig. 2:** Microtumor volume after 5 days of incubation (TB 32047 WT: n = 5, NC1.4: n = 14, STK11 KO1.5: n = 7, STK11 KO 2.2: n = 11). Data are presented as means of three independent experiments. *, p< 0.05 by one-way ANOVA.

**
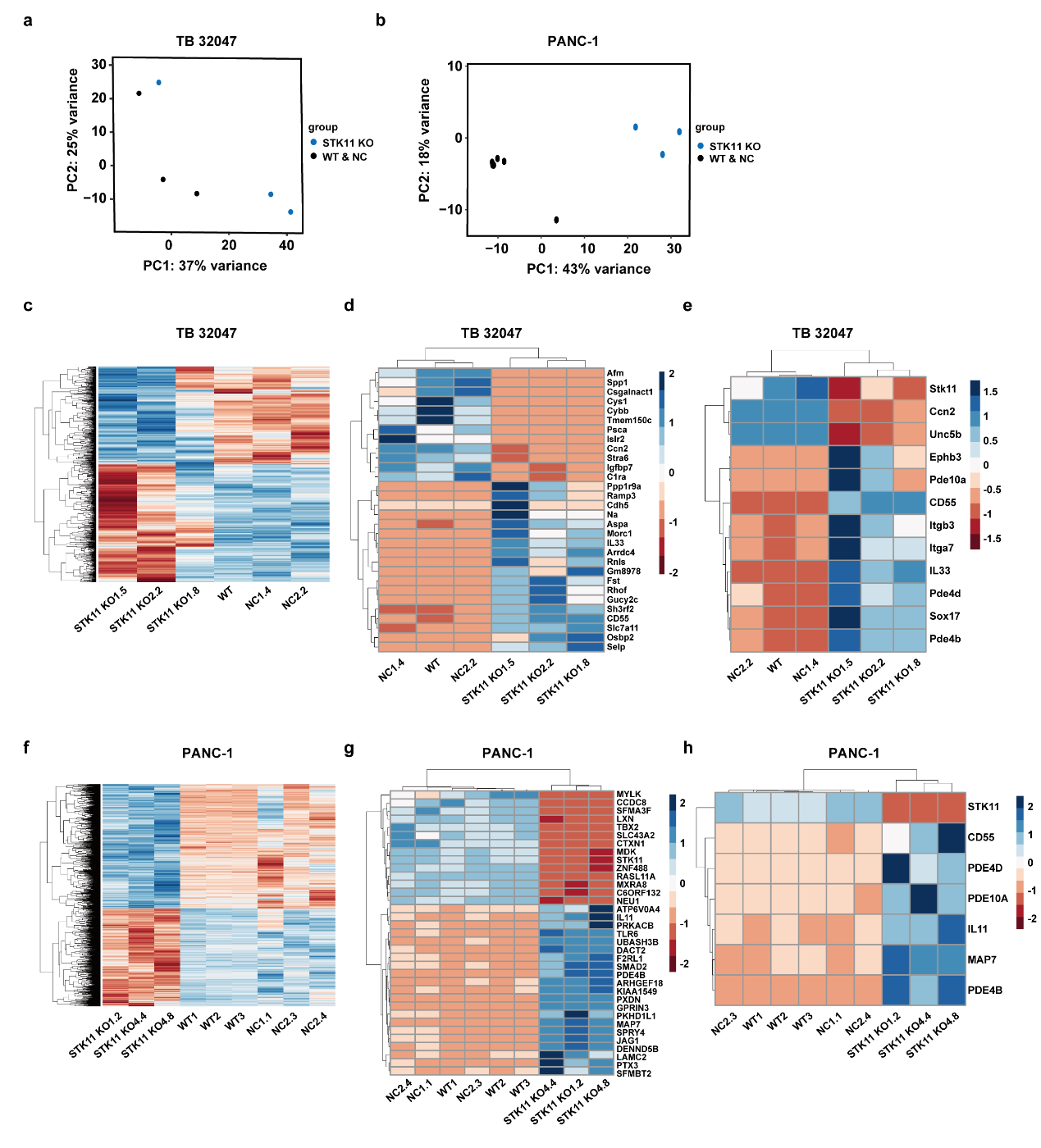
**

**Supplementary Fig. 3: Differential expression analysis of edited TB 32047 and PANC-1 genes.** Principal component analysis (PCA) based on the normalized logarithm-transformed read counts of DEGs. PCA verified that most of the variance (**a** 37%, PC1 of TB 32047; **b** 43% PC1 of PANC-1) was associated with the altered expression between STK11 knock-out samples and the control samples. **c, f** Heatmap and hierarchical clustering of the samples and the DEGs of TB 32047 and PANC-1 samples. Rows have been centered and scaled to compute z-scores. **d, g** Heatmap plot of the TOP 30 DEGs (using p adj values) of TB 32047 and PANC-1 samples. The blue denotes the expression of upregulated DEGs; the red denotes the expression of downregulated DEGs. **e** Heatmap of differentially expressed genes (*Stk11*, *Ccn2*, *Unc5b*, *Wnt4*, *Ephb3*, *Pde10a*, *CD55*, *Itgb3*, *Itga7*, *IL33*, *Pde4d*, *Sox17*, *Pde4b*) in TB 32047 samples. **h,** Heatmap of differentially expressed genes (*STK11*, *CD55*, *PDE4D*, *PDE10A*, *IL11*, *MAP7*, *PDE4B*) in PANC-1 samples. blue = high, red = low, normalized to genes.

**
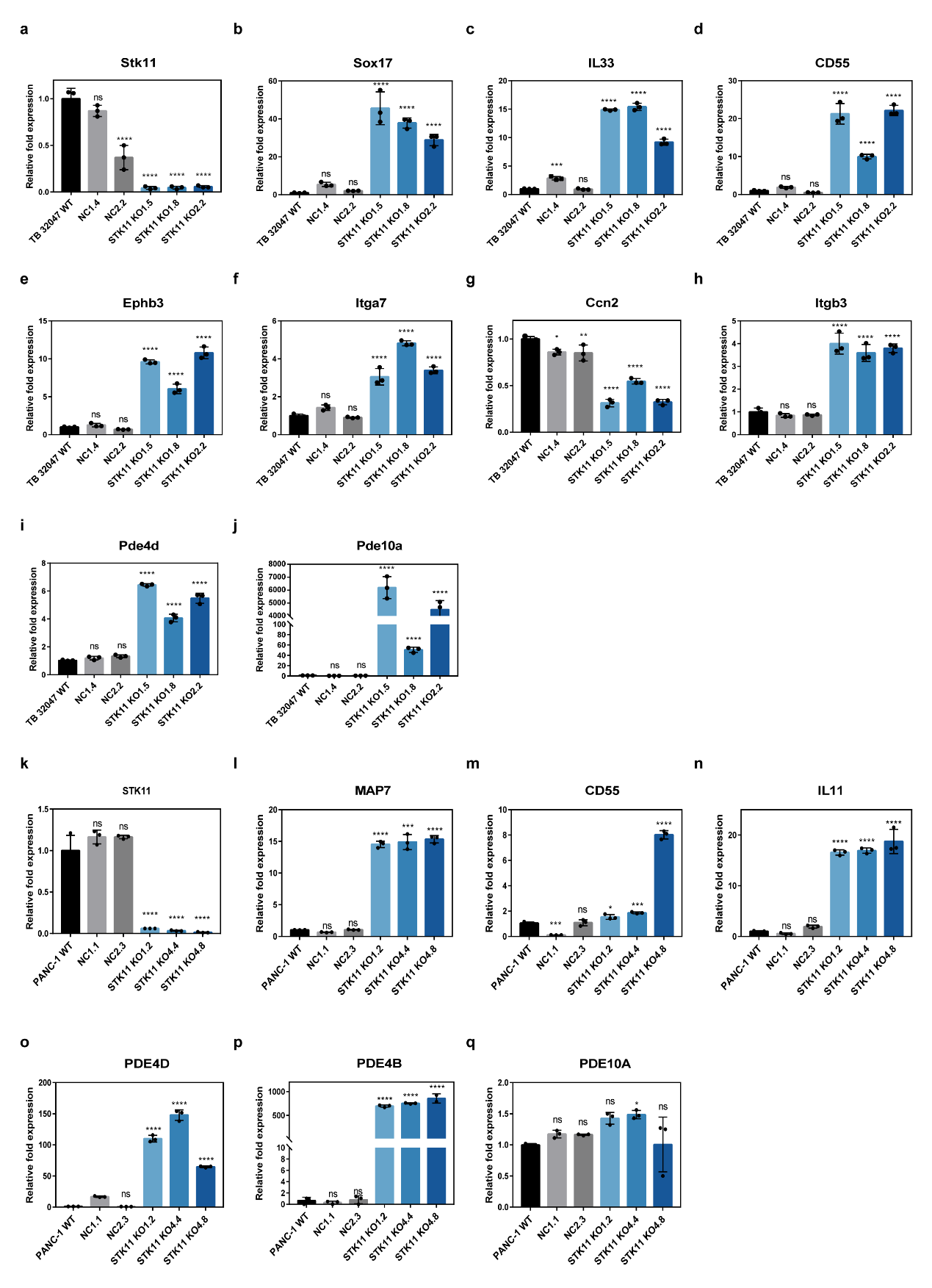
**

**Supplementary Fig. 4: RT-qPCR verified that the significant change gene is induced by loss of STK11 in TB 32047 and PANC-1 samples. a-j** Gene mRNA levels (*Stk11*, *Sox17*, *IL33*, *CD55*, *Ephb3*, *Itga7*, *Ccn2*, *Itgb3*, *Pde4d*, *Pde10a*) were examined in TB 32047 samples. **k-q** Gene mRNA levels (*STK11*, *MAP7*, *CD55*, *IL11*, *PDE4D*, *PDE4B*, *PDE10A*) were examined in PANC-1 samples. Data are presented as means of three independent experiments. *, p< 0.05; **, p< 0.01; ***, p< 0.001; ****, p< 0.0001 by one-way ANOVA.

**
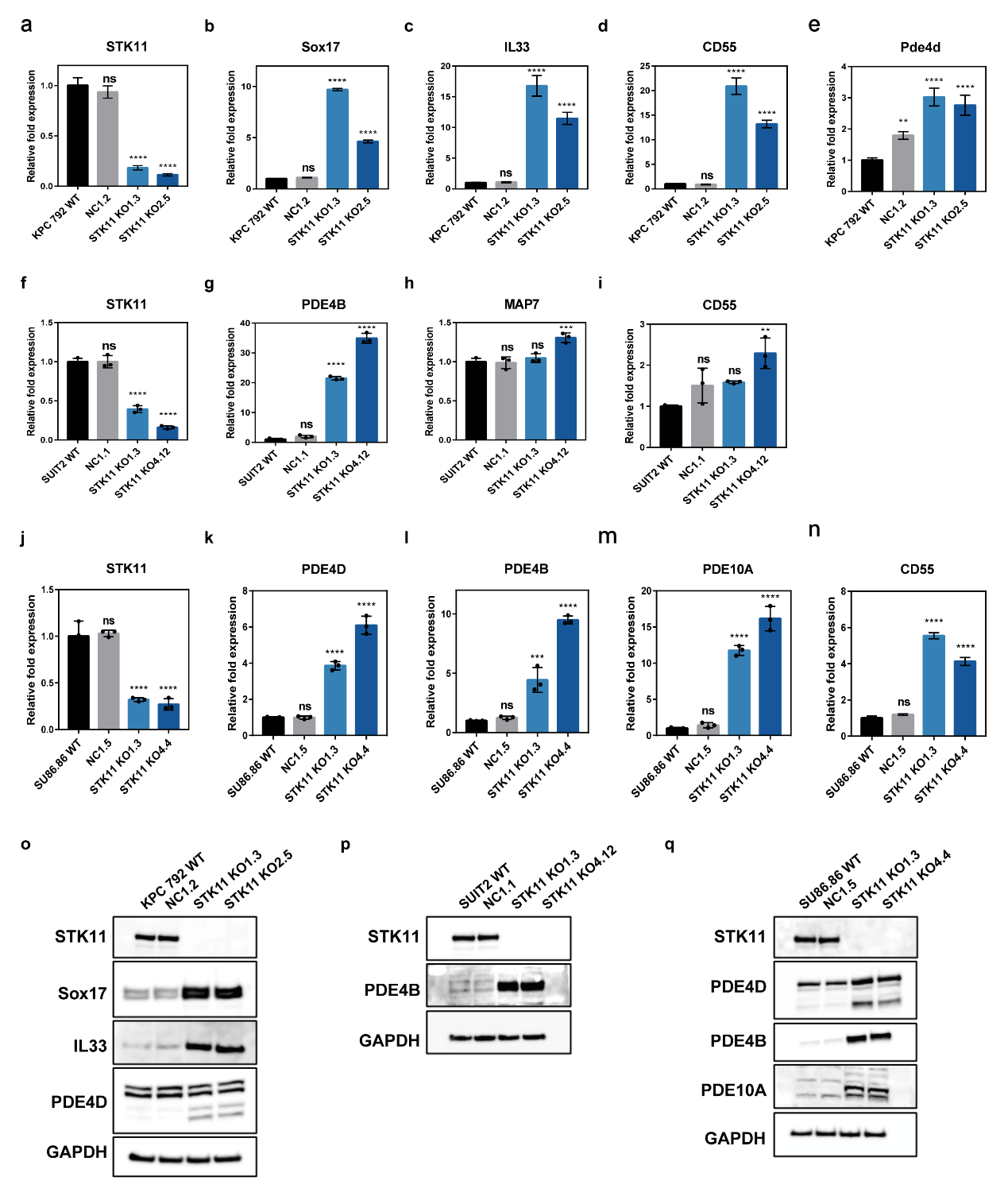
**

**Supplementary Fig. 5: RT-qPCR and western blot verified that the significant change gene is induced by loss of STK11 in KPC 792, SUIT2 and SU86.86 samples. a-e** Gene mRNA levels (*Stk11*, *Sox17*, *IL33*, *CD55*, *Pde4d*) were examined in KPC 792 samples. **f-i** Gene mRNA levels (*STK11*, *PDE4B*, *MAP7*, *CD55*) were examined in SUIT2 samples. **j-n** Gene mRNA levels (*STK11*, *PDE4D*, *PDE4B*, *PDE10A*, *CD55*) were examined in SU86.86 samples. For (**a-n**), data are presented as means of three independent experiments. *, p< 0.05; **, p< 0.01; ***, p< 0.001; ****, p< 0.0001 by one-way ANOVA. **o** Western blot analysis of STK11, Sox17, IL33 and PDE4D in STK11^KO^ of KPC 792 samples. **p** Western blot analysis of STK11 and PDE4B in STK11^KO^ of SUIT2 samples. **q** Western blot analysis of STK11, PDE4D, PDE4B and PDE10A in STK11^KO^ of SU86.86 samples. For (**o-q**), GAPDH was used as the loading control.

**
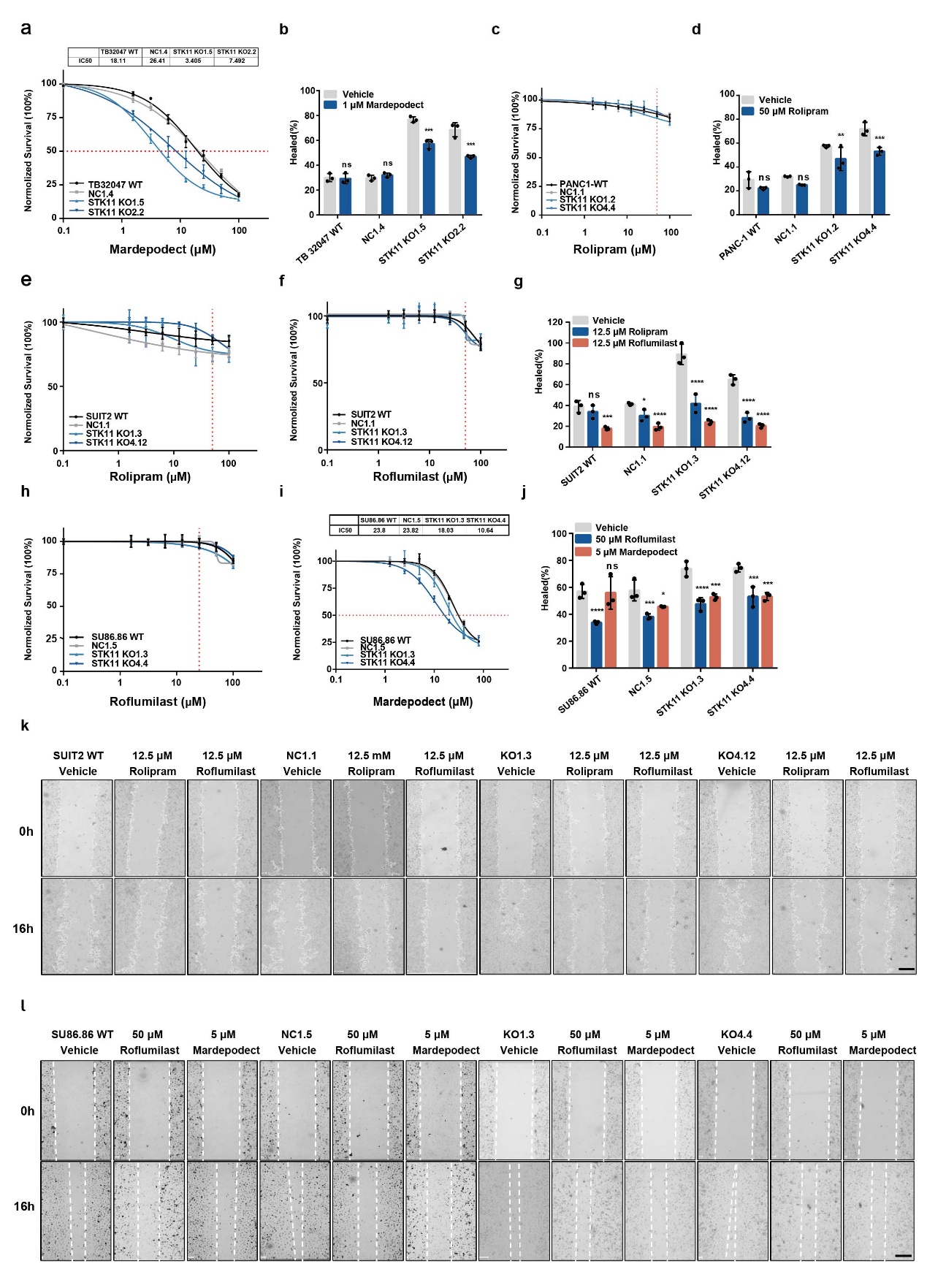
**

**Supplementary Fig. 6: PDE inhibitors attenuate STK11-mutated induced migration in PDAC cells. a** TB 32047 cells were exposed to increasing doses of Mardepodect. **b** Quantitative analysis of TB 32047 cells migrated with or without 1 µM Mardepodect after 24 h. Data are presented as means of three independent experiments. ***, p< 0.001 by one-way ANOVA. **c** PANC-1 cell lines was exposed to increasing doses of Rolipram. **d** Quantitative analysis of PANC-1 cells migrate with or without 50 µM Rolipram after 36 h. Data are presented as means of three independent experiments. **, p< 0.01; ***, p< 0.001 by one-way ANOVA. **e, f** SUIT2 cells were exposed to increasing doses of Rolipram and Roflumilast. **g** Quantitative analysis of SUIT2 cells migrated with or without 12.5 µM Rolipram or 12.5 µM Roflumilast after 16 h. Data are presented as means of three independent experiments. *, p< 0.05; ***, p< 0.001; ****, p< 0.0001 by one-way ANOVA. The images were taken at 0 h and 16 h are shown in **k**, respectively. **h, i** SU86.86 cells were exposed to increasing doses of Roflumilast and Mardepodect. For (**a**, **c**, **e**, **f**, **h**, **i**,) the percentage of cell viability relative to that of cells was shown. Data represent mean ± SD of three replicates. **j** Quantitative analysis of SU86.86 cells migrated with or without 50 µM Roflumilast or 5 µM Mardepodect after 16 h. Data are presented as means of three independent experiments. *, p< 0.05; ***, p< 0.001; ****, p< 0.0001 by one-way ANOVA. Images taken at 0 h and 16 h are shown in **l**.

**
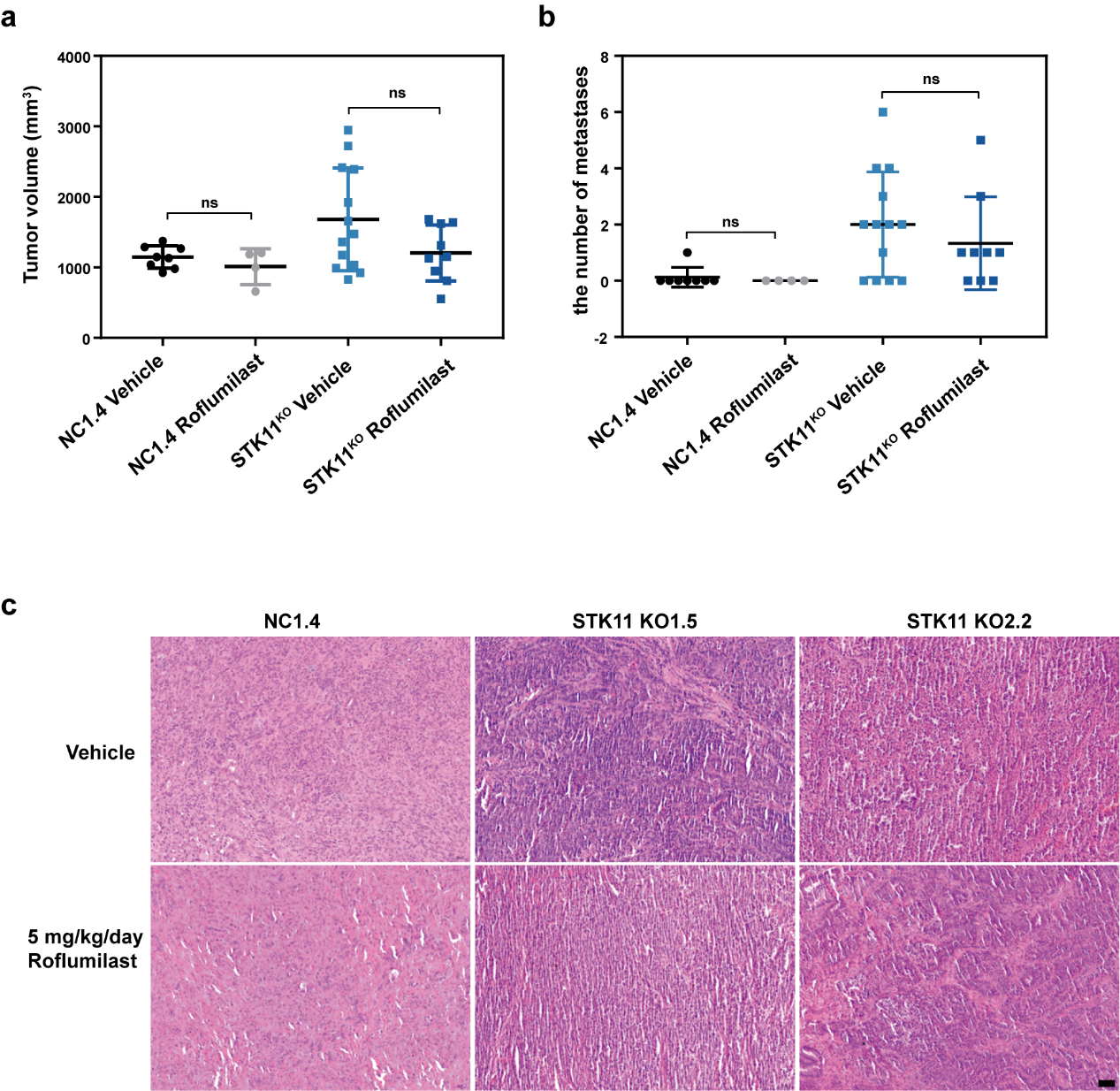
**

**Supplementary Fig. 7: Effect of Roflumilast on tumor progression and metastasis in the orthotopic pancreatic cancer mouse model.** **a** Pancreatic tumors were collected on day 23 (day 8-23 with Roflumilast), measured compared to the Vehicle group. Data represented the mean ± SD of at least 4 mice per group. ns, p = 0.2807 (NC1.4 Vehicle vs. NC1.4 Roflumilast); ns, p = 0.0913 (STK11^KO^ Vehicle vs. STK11^KO^ Roflumilast) by unpaired T-test. **b** The livers of mice were collected on day 23 (day 8-23 with Roflumilast), counting the metastasis number compared to the Vehicle group. Data represented the mean ± SD of at least 4 mice per group. ns, p = 0.5059 (NC1.4 Vehicle vs. NC1.4 Roflumilast); ns, p = 0.2001 (STK11^KO^ Vehicle vs. STK11^KO^ Roflumilast) by unpaired T-test. **c** H&E staining of the pancreatic tumors. Scale—50 μm.

**
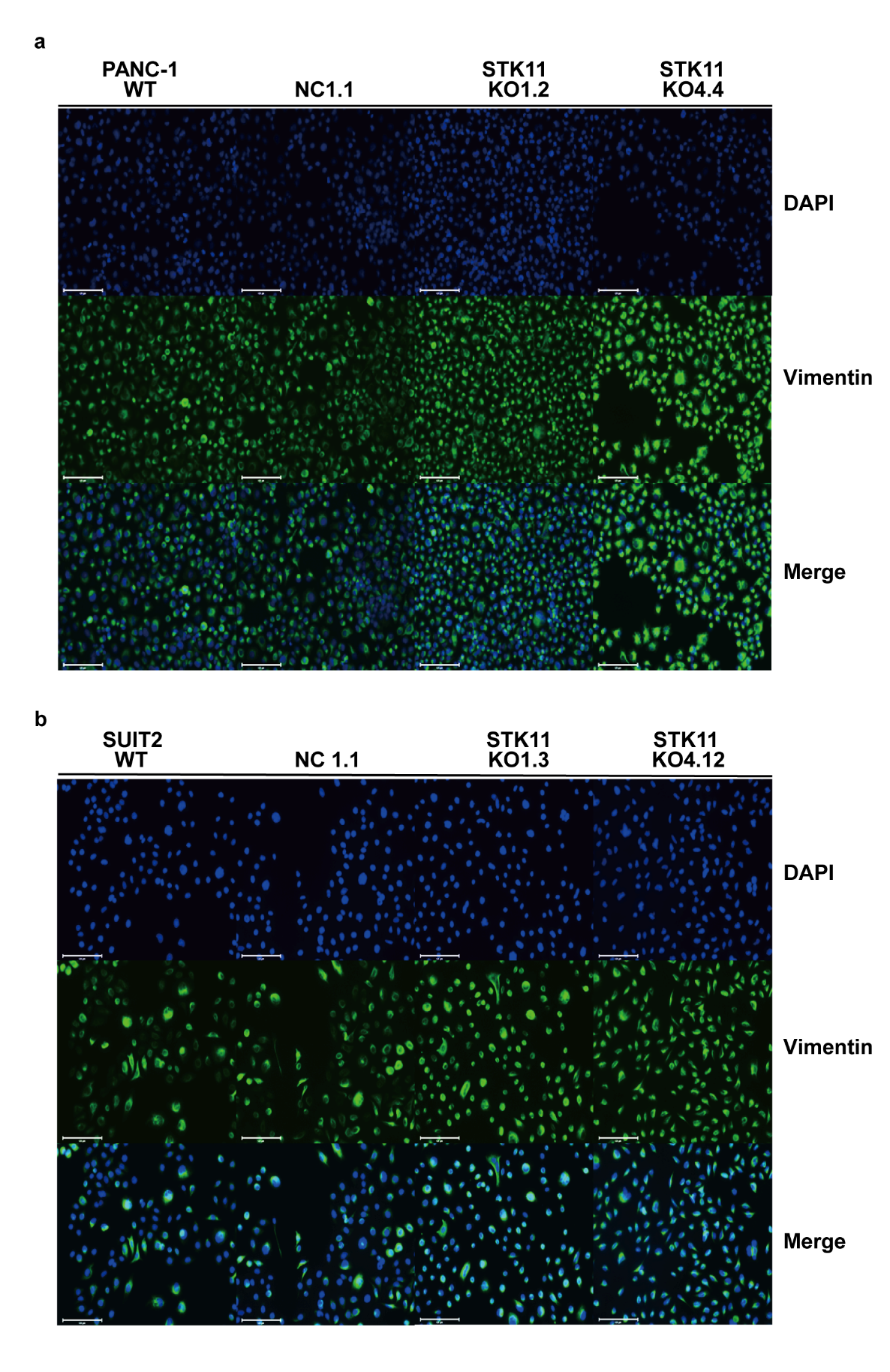
**

**Supplementary Fig. 8: STK11 regulates the expression of Vimentin in human PDAC cells. a, b** Representative images showed immunofluorescent staining for vimentin and nucleus (DAPI) of WT and STK11^KO^ PANC-1 and SUIT2 cells. Green denotes the anti-Vimentin (green) antibody, and blue indicates the nucleus of stained with DAPI. Scale bar: 125 µm. Images are representative of three biologically independent experiments.

**
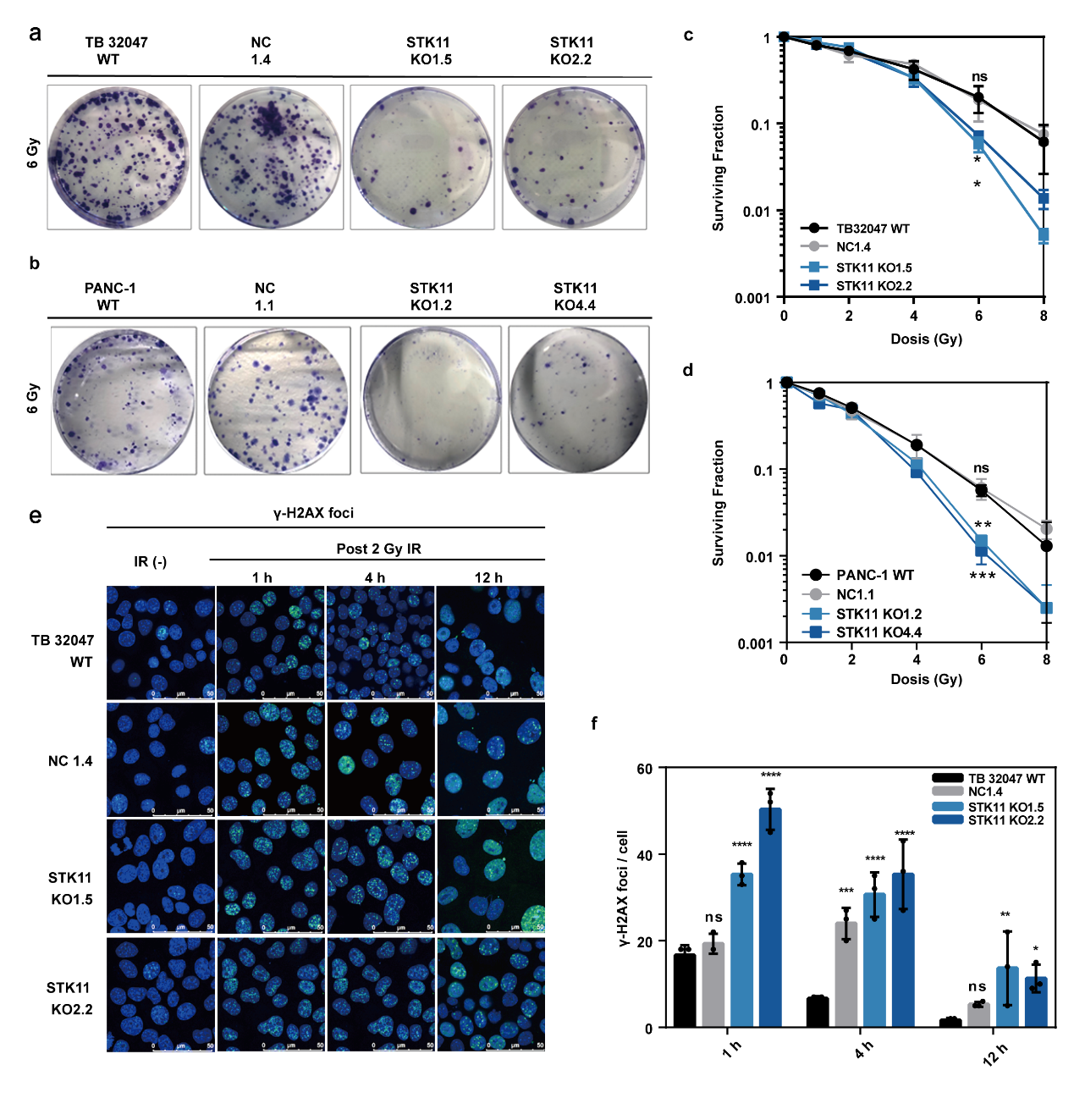
**

**Supplementary Fig. 9: STK11 knockout increases the efficacy of radiotherapy and DNA damage after radiotherapy. a,** **b** STK11^KO^ and control cells were treated with different doses of radiation followed by clonogenic assays. Representative images from three independent experiments (n = 3) are shown. **c, d** A clonogenic assay of TB 32047 and PANC-1 cells with STK11^KO^ and control cells was performed after irradiation with different doses of X-rays. The colony numbers were counted and normalized. Data are presented as means of three independent experiments (n = 3). **e** Immunofluorescence of the TB32047 control (WT and NC) and STK11^KO^ at different time points (1 h, 4 h, 12 h) with 2 Gy irradiation. Representative images of three independent experiments (n = 3) and statistical analysis are shown. (Blue-DAPI, Green-γH2A.X. Scale bar: white, 50 µm). **f** Comparative changes in the γH2AX foci in cells exposed to 2 Gy irradiation. *, p< 0.05; **, p< 0.01; ***, p< 0.001; ****, p< 0.0001 by one-way ANOVA.

**Supplementary Table 1: List of cell culture medium**

| **Cell lines** | **Medium components** |
| --- | --- |
| PANC-1 | RPMI-1640 Medium (Gibco™, #21875034) with 10% FBS (Gibco™, #A3160501) |
| Aspc1 |  |
| SU86.86 |  |
| Panc05.04 |  |
| SUIT2 | Minimum Essential Medium (Gibco™, #11095080) with 10% FBS |
| Miapaca2 | Dulbecco's Modified Eagle's Medium with 10% FBS (Gibco™, #A3160501) and 12.5 ml horse Serum (Gibco™, #16050122) |
| TB 32047 | Dulbecco's Modified Eagle's Medium (Gibco™, #11965092) with 10% FBS |
| KPC 792 |  |
| Mayo4636 | DMEM/F-12 (Thermo Fisher, # 11330032) with 10% FBS, 5 ml L-Glutamin (200 mM) and 7.5 ml HEPES (1M) |
| HEK293TN | Dulbecco's Modified Eagle's Medium (Gibco™, #11965092) with 10% heat-inactivated FBS |
| TKCC-10 | 215 ml Medium 199, 215 ml Ham's F-12 Nutrient Mix, 50 ml FBS, 7.5 ml HEPES (1M), 10 µl EGF (Stock: 1mg/ml), 20 µl Hydrocortisone (Stock: 1mg/ml), 5 ml apo-Transferrin Human (Stock: 2.5 mg/ml), 1 ml Insulin, human recombinant, zinc solution (Stock: 100 IU/ml), 3 ml Glucose solution (10%), 2.5 ml MEM Vitamin Solution (100×), 2.5 µl 3,3′,5-Triiodo-L-thyronine sodium salt (Stock: 0,1 µg/ml), 50 µl O-Phosphorylethanolamine (Stock: 20 mg/ml), 5 ml 2 mM Glutamine (100× stock) |
| Organoid  feeding medium | 36.415 ml Wash Medium, 50 ml 2× Wnt3a-Conditioned Medium, 10 ml 10× R-spondin1-Conditioned Medium, 2 ml 50× B27 Supplement, 1 ml 1 M Nicotinamide (Sigma-Aldrich, #N0636-100G), 250 µl 500 mM N-acetylcysteine (Sigma-Aldrich, #A9165-5G), 200 µl 50 mg/ml Primocin, 100 µl 100 µg/ml mNoggin (Peprotech, #250-38), 10 µl 500 µg/ml hEGF (Thermo Fisher, #PMG8043), 10 µl 1000 µg/ml hFGF (Peprotech, #100-26), 10 µl 100 µM hGastrin I (TOCRIS, #3006), 2 µl 25 mM A 83-01 (TOCRIS, #2939), 100 µl 10.5 mM Y-27632 |
| Organoid  washing medium | 500 ml Advanced DMEM/F-12 (Thermo Fisher, #12634010), 5 ml 1 M HEPES pH 7.2-7.5 (Thermo Fisher, #15630080), 5 ml 100× GlutaMAX Supplement (Thermo Fisher, #35050061), 1 ml 50 mg/ml Primocin (Invivogen, #ant-pm-2), 1.67 ml 30% Bovine Serum Albumin solution (Sigma-Aldrich, #A9576) |
| Organoid  digestion medium | 10 ml TrypLE™ Express Enzym (1×) (Gibco™, #12605010),10 µl 10.5 mM Y-27632 (Sigma-Aldrich, #Y0503-5mg), 10 µl 10 mg/ml DNAse I (Sigma-Aldrich, #D5025-150KU) |

**Supplementary Table 2: Primers designed for sgRNA**

| **Species** | **Gene symbol** | **sgRNA** | **Forward primer**  **(5’-3')** | **Reverse primer**  **(5’-3')** | **Note** |
| --- | --- | --- | --- | --- | --- |
| **mouse** | STK11 | ACTCCATCACCATATACGTA | CACCGACTCCATCACCATATACGTA | AAACTACGTATATGGTGATGGAGTC | sgRNA1 |
|  |  | AGTCCATTGGCAATCTCAGG | CACCGAGTCCATTGGCAATCTCAGG | AAACCCTGAGATTGCCAATGGACTC | sgRNA2 |
|  | Non-Target Control | AACCCCGGCTGTCATCGCCG | CACCGAACCCCGGCTGTCATCGCCG | AAACCGGCGATGACAGCCGGGGTTC | sgRNA1 |
|  |  | ACGCGAAGTGTCGCAGAGTG | CACCGACGCGAAGTGTCGCAGAGTG | AAACCACTCTGCGACACTTCGCGTC | sgRNA2 |
| **human** | STK11 | TGCACAAGGACATCAAGCCG | CACCGTGCACAAGGACATCAAGCCG | AAACCGGCTTGATGTCCTTGTGCAC | sgRNA1 |
|  |  | GTTGTTTGAGAACATCGGGA | CACCGGTTGTTTGAGAACATCGGGA | AAACTCCCGATGTTCTCAAACAACC | sgRNA4 |
|  | Non-Target Control | GAAATGCTATGCTTCGGTTC | CACCGGAAATGCTATGCTTCGGTTC | AAACGAACCGAAGCATAGCATTTCC | sgRNA1 |
|  |  | CGGGACGTCGCGAAAATGTA | CACCGCGGGACGTCGCGAAAATGTA | AAACTACATTTTCGCGACGTCCCGC | sgRNA2 |

**Supplementary Table 3: Primers designed for RT-PCR**

| **Species** | **Gene symbol** | **Forward primer (5'-3')** | **Reverse primer (5'-3')** |
| --- | --- | --- | --- |
| mouse | β-actin | GTGACGTTGACATCCGTAAAGA | GCCGGACTCATCGTACTC |
|  | GAPDH | AGGTCGGTGTGAACGGATTTG | TGTAGACCATGTAGTTGAGGTCA |
|  | Stk11 | CTGGACTCCGAGACCTTATGC | CAAGCTGGATCACATTCCGAT |
|  | Ephb3 | CATGGACACGAAATGGGTGAC | GCGGATAGGATTCATGGCTTCA |
|  | IL33 | ATTTCCCCGGCAAAGTTCAG | AACGGAGTCTCATGCAGTAGA |
|  | Sox17 | ACCTACACTTACGCTCCAGTC | GCCGTAGTACAGGTGCAGAG |
|  | CD55 | ACCTCCACTCCCAGGAAAAG | TAGAGGAGACACCGACTAGCC |
|  | Ccn2 | GACCCAACTATGATGCGAGCC | CCCATCCCACAGGTCTTAGAAC |
|  | Itga7 | CTGCTGTGGAAGCTGGGATTC | CTCCTCCTTGAACTGCTGTCG |
|  | Itgb3 | GATTGCCCTTCGACTACGGC | GTCCACGGGGTAATCCTCCA |
|  | Pde4d | CCAGAATCTGACCAAAAAGCAA | GAAGAACTCCTCCATTATCCGG |
|  | Pde10a | TGGCCAGTTACAAAATTGACAG | CCATCATAGGAATGGGCTGTAT |
| human | β-ACTIN | CACCATTGGCAATGAGCGGTTC | AGGTCTTTGCGGATGTCCACGT |
|  | GAPDH | CTTTGGTATCGTGGAAGGACTC | AGTAGAGGCAGGGATGATGT |
|  | STK11 | TCTACAACATCACCACGGGTC | TTCGTACTCAAGCATCCCTTTC |
|  | IL11 | GCGGACAGGGAAGGGTTAAAG | AGGCGGCAAACACAGTTCA |
|  | PDE4D | AGGTTCTTCAGAATATGGTGCA | GAAGAACTCCTCCATTATCCGG |
|  | PDE4B | GTGTTGCTCAGGAAACTTACAG | ATTTTCCACATCAAAGCACTCC |
|  | PDE10A | TATAGAACAACGGTTGGACACA | AAATACAGTGCAAATCCATCGG |
|  | SOX17 | CTCCGGTGTGAATCTCCCC | CACGTCAGGATAGTTGCAGTAAT |
|  | CD55 | CCAGGTTACAGAAGAGAACCTT | TTACAAAATTCGACTGCTGTGG |

**Supplementary Table 4**: Primers designed for the knockout check

| **Species** | **Gene symbol** | **sgRNA** | **Forward primer**  **(5'-3')** | **Reverse primer**  **(5'-3')** |
| --- | --- | --- | --- | --- |
| Mouse | *STK11* | ACTCCATCACCATATACGTA | CTACGTTGCATCTGGGCAGTCCTGG | TTCCTGACAATGGCTGCTCCTCCTG |
|  |  | AGTCCATTGGCAATCTCAGG | AGCTGATTGACGGCCTGGAATAC | TGCAAGCAAAACCCTGTGGAGTG |
| Human | *STK11* | GTTGTTTGAGAACATCGGGA | CCACCTTGACTGACCACGCCTTTC | GAGCTGAGGGACCTGGCAAACC |

**Supplementary Table 5: Sanger sequence for knockouts**

| **Cell line** | **Knockouts** | **Mutations** | **Cell line** | **Knockouts** | **Mutations** |
| --- | --- | --- | --- | --- | --- |
| **TB 32047** | STK11 KO1.5 | Mut1: delete 1 bp  Mut2: insert 1 bp | **Panc1** | STK11 KO1.2 | Mut1: delete 48 bp |
|  | STK11 KO1.8 | Mut1: delete 2 bp |  | STK11 KO4.4 | Mut1: insert 1 bp |
|  | STK11 KO2.2 | Mut1: delete 1 bp |  | STK11 KO4.8 | Mut1: insert 1 bp |
| **KPC 792** | STK11 KO1.3 | Mut1: insert 1 bp Mut2: insert 53 bp | **Suit2** | STK11 KO1.3 | Mut1: delete 2 bp |
|  | STK11 KO2.5 | Mut1: insert 1 bp |  | STK11 KO4.12 | Mut1: delete 1 bp |
| **SU86.86** | STK11 KO1.3 | Mut1: delete 1 bp | **SU86.86** | STK11 KO4.4 | Mut1: delete 2 bp |

**Supplementary Table 6: List of antibodies and inhibitors**

| **Company** | **Antibodies** | **Identifier** |
| --- | --- | --- |
| Cell signaling | GAPDH | Cat# 5174,  RRID: AB_10622025 |
|  | Anti-rabbit IgG, HRP-linked | Cat# 7074,  RRID: AB_2099233 |
|  | Anti-mouse IgG, HRP-linked | Cat# 7076,  RRID: AB_330924 |
|  | STK11 (D60C5) Rabbit mAb | Cat# 3047,  RRID: AB_2198327 |
|  | Vimentin (D21H3) XP® Rabbit mAb | Cat# 5741,  RRID: AB_10695459 |
|  | phospho-Histone H2A.X (Ser139) | Cat # 2577,  RRID: AB_2118010 |
| Santa Cruz | Anti-IL-33 Antibody (4E9) | Cat# sc-130625, RRID: AB_2233601 |
|  | Anti-STK11 Antibody (Ley 37D/G6) | Cat# sc-32245, RRID: AB_627890 |
| Abcam | Recombinant Anti-SOX17 antibody | Cat #ab224637, RRID: AB_2801385 |
|  | PDE4D | Cat #Ab171750, RRID: AB_2927554 |
|  | PDE4B | Cat #Ab170939, RRID: AB_2927553 |
|  | PDE10A | Cat #Ab227829, RRID: AB_2927552 |
| Invitrogen | Goat anti-Rabbit IgG (H+L) Highly Cross-Adsorbed Secondary Antibody, Alexa Fluor™ 488 | Cat #A11034, RRID: AB_2576217 |
| Selleck | Rolipram (ZK-62711) | Cat #S1430 |
|  | Roflumilast (B9302-107) | Cat #S2131 |
|  | Mardepodect (PF-2545920) | Cat #2687 |
| Hycultec | Roflumilast | Cat #HY-15455 |
|  | Mardepodect | Cat #HY-50098 |
