# Supplementary material for: Roflumilast inhibits tumor growth and migration in STK11/LKB1 deficient pancreatic cancer
Source: Cell Death Discov. 2024 Mar 9;10:124. doi: 10.1038/s41420-024-01890-y (PMC10924943; doi:10.1038/s41420-024-01890-y)
Supplement: Supplementary file 2 — Supplemental Material_Western Blot [file 41420_2024_1890_MOESM2_ESM.docx]

**Full length uncropped original western blots**

**Fig.1f（STK11;GAPDH）**


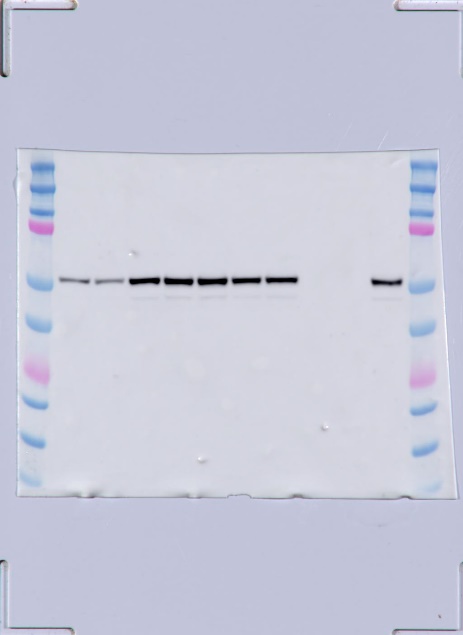

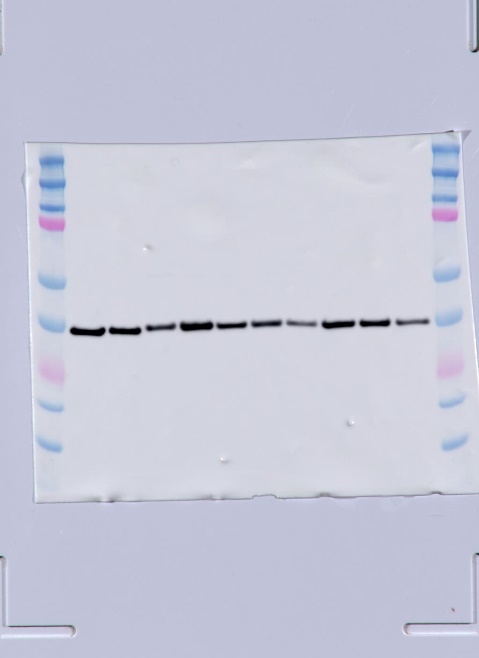


**Fig.2c（STK11;GAPDH）**


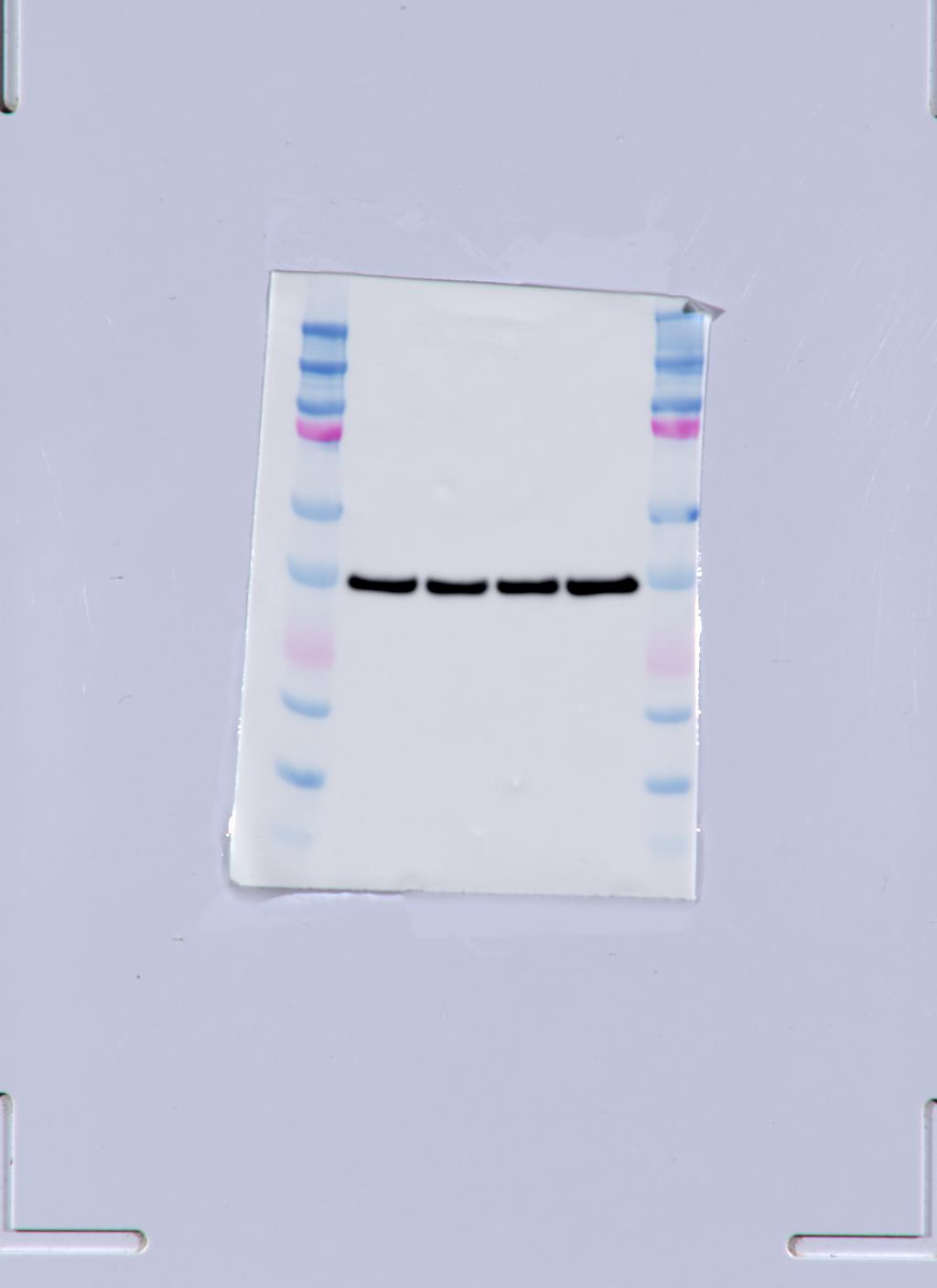

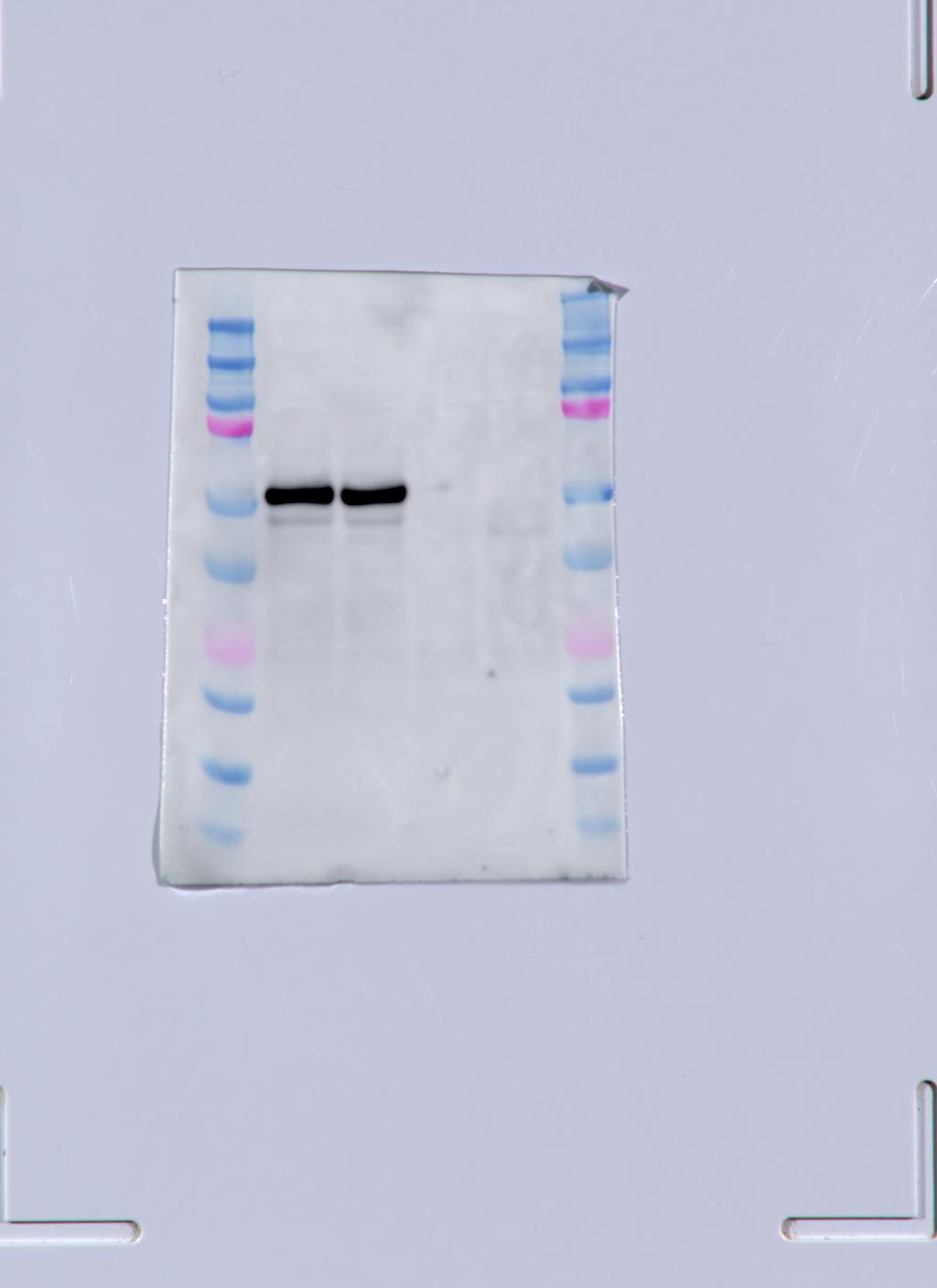


**Fig.2d(STK11;GAPDH)**


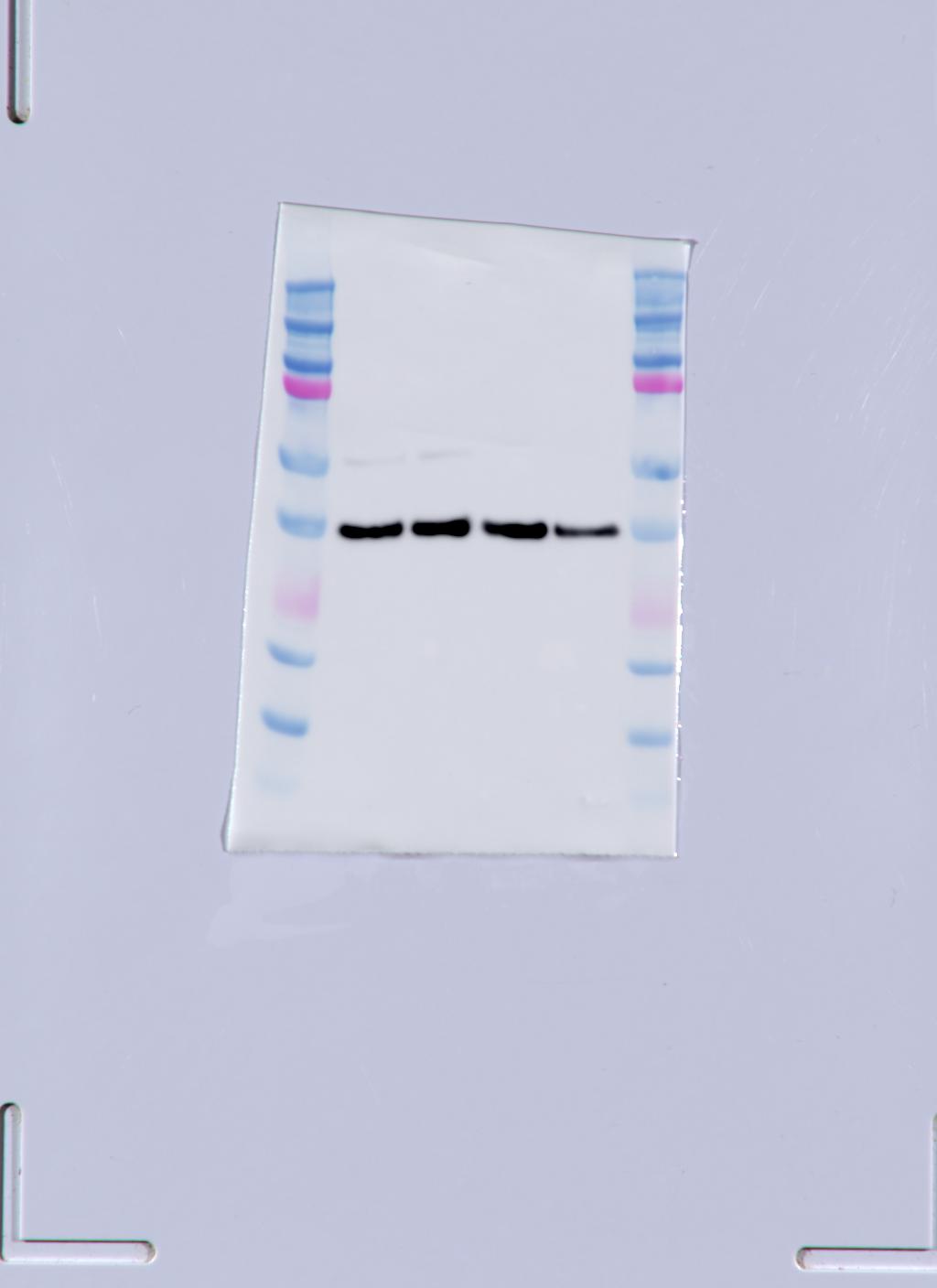

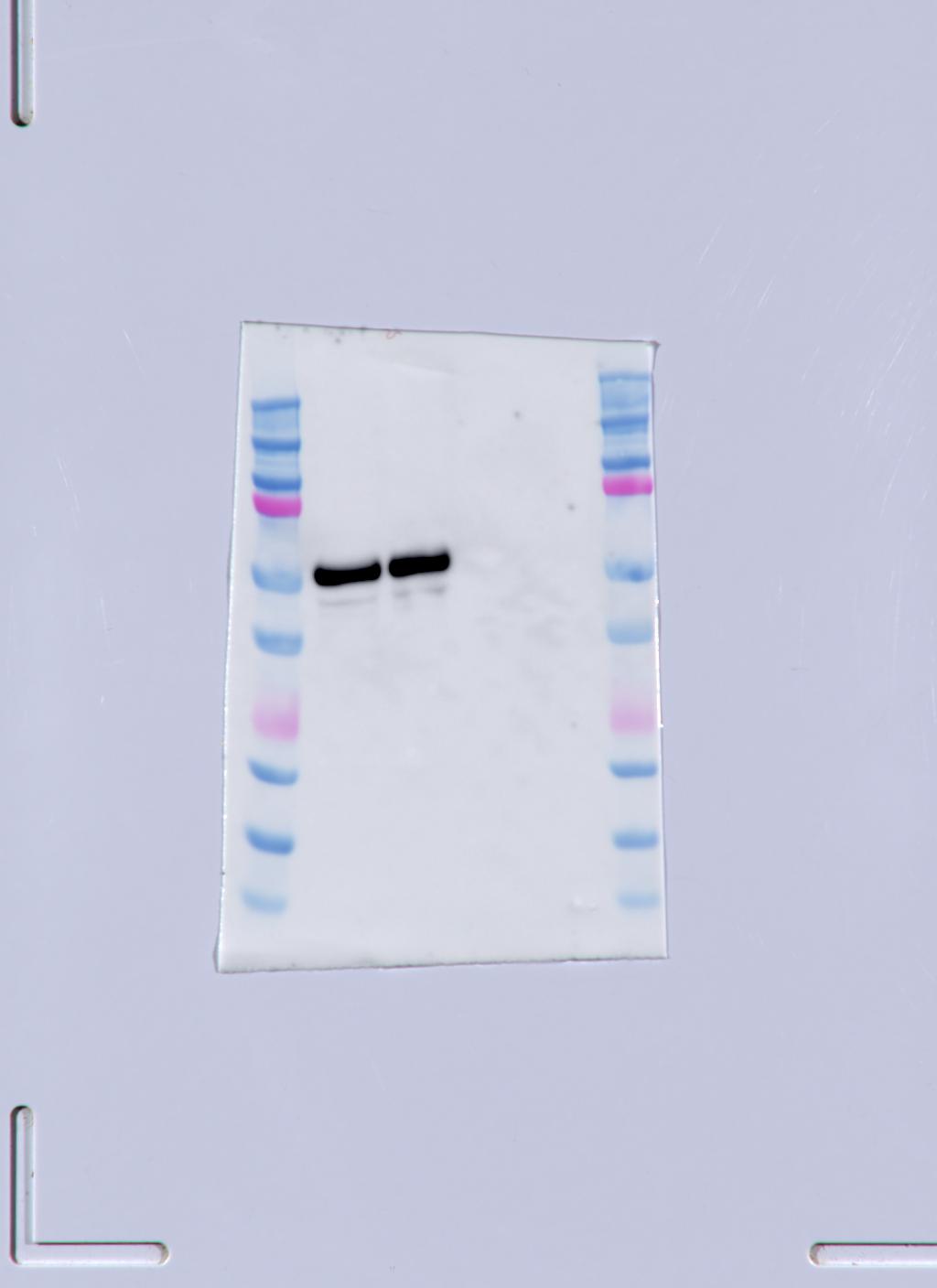


**Fig.5f（Sox17;IL33;GAPDH）**

**
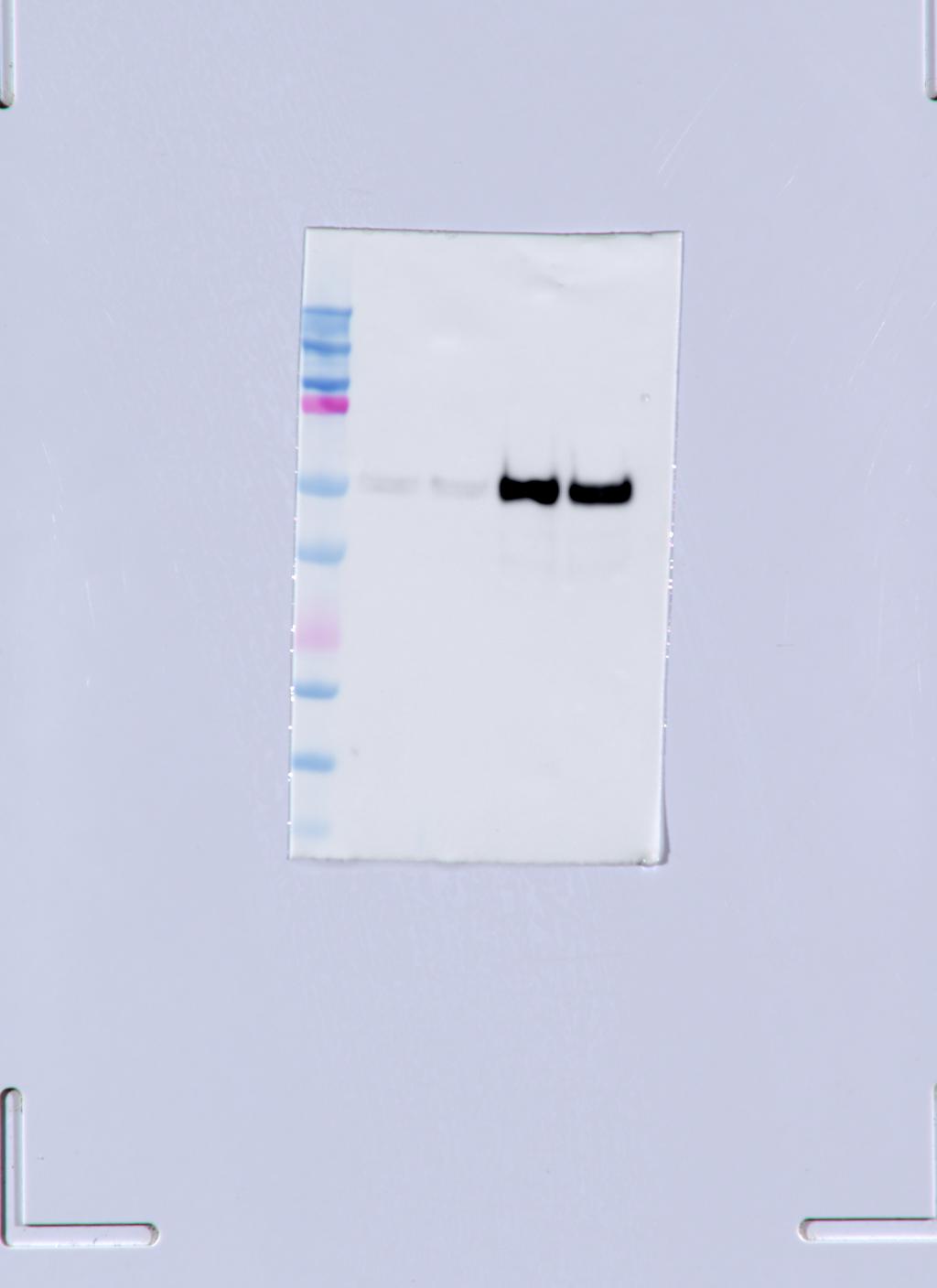

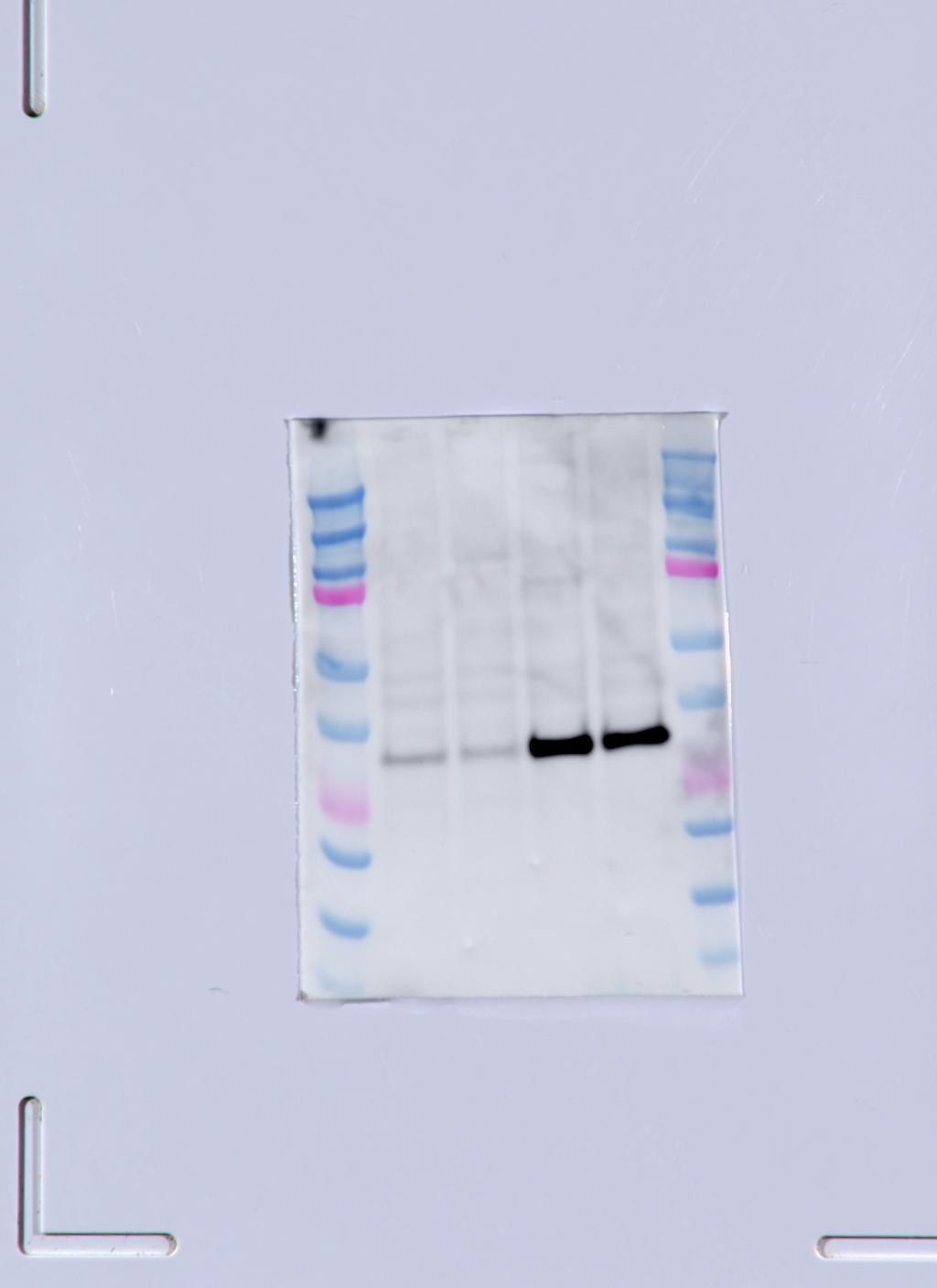

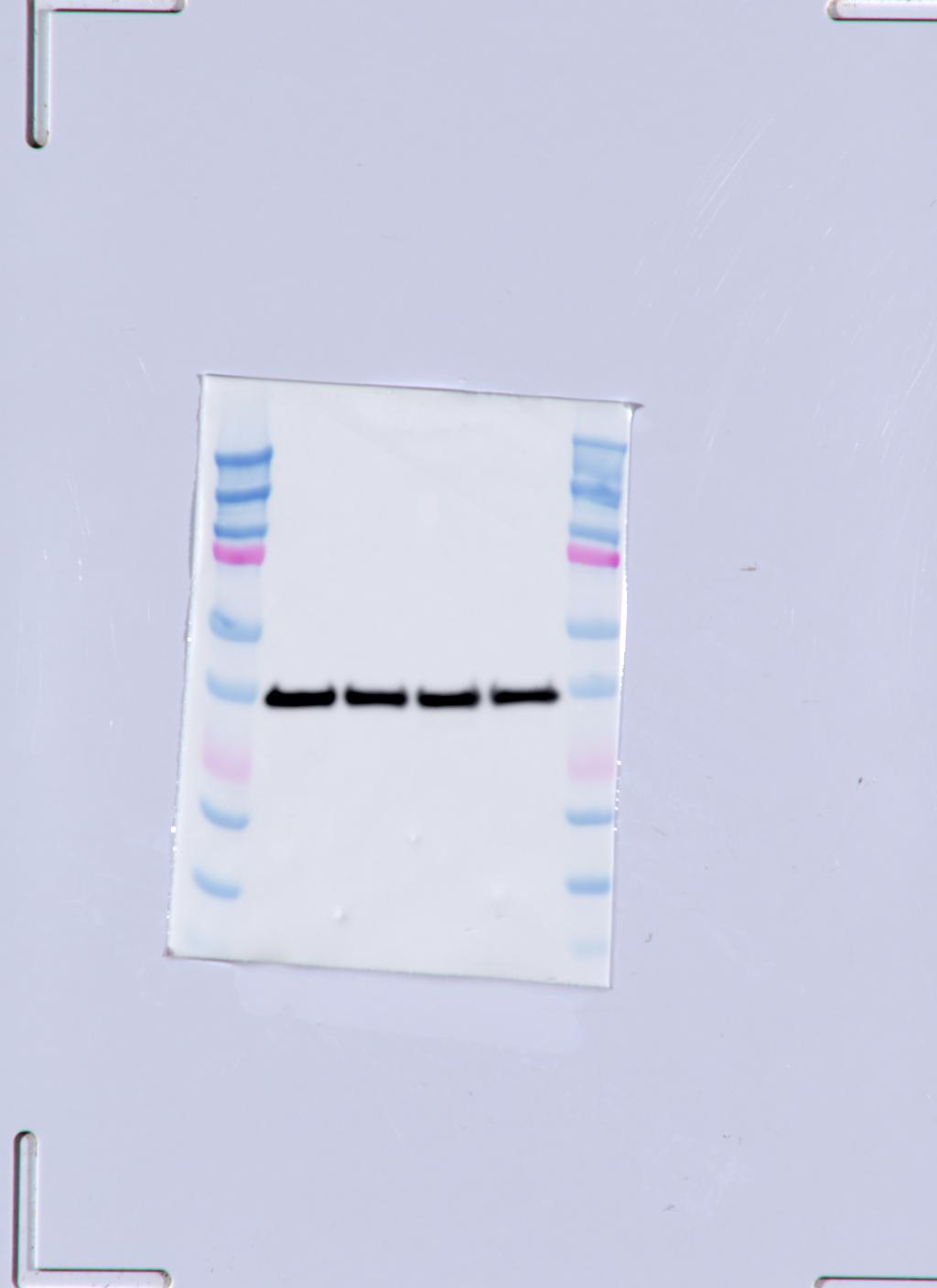
**

**（PDE4D;PDE10A;GAPDH）**

**
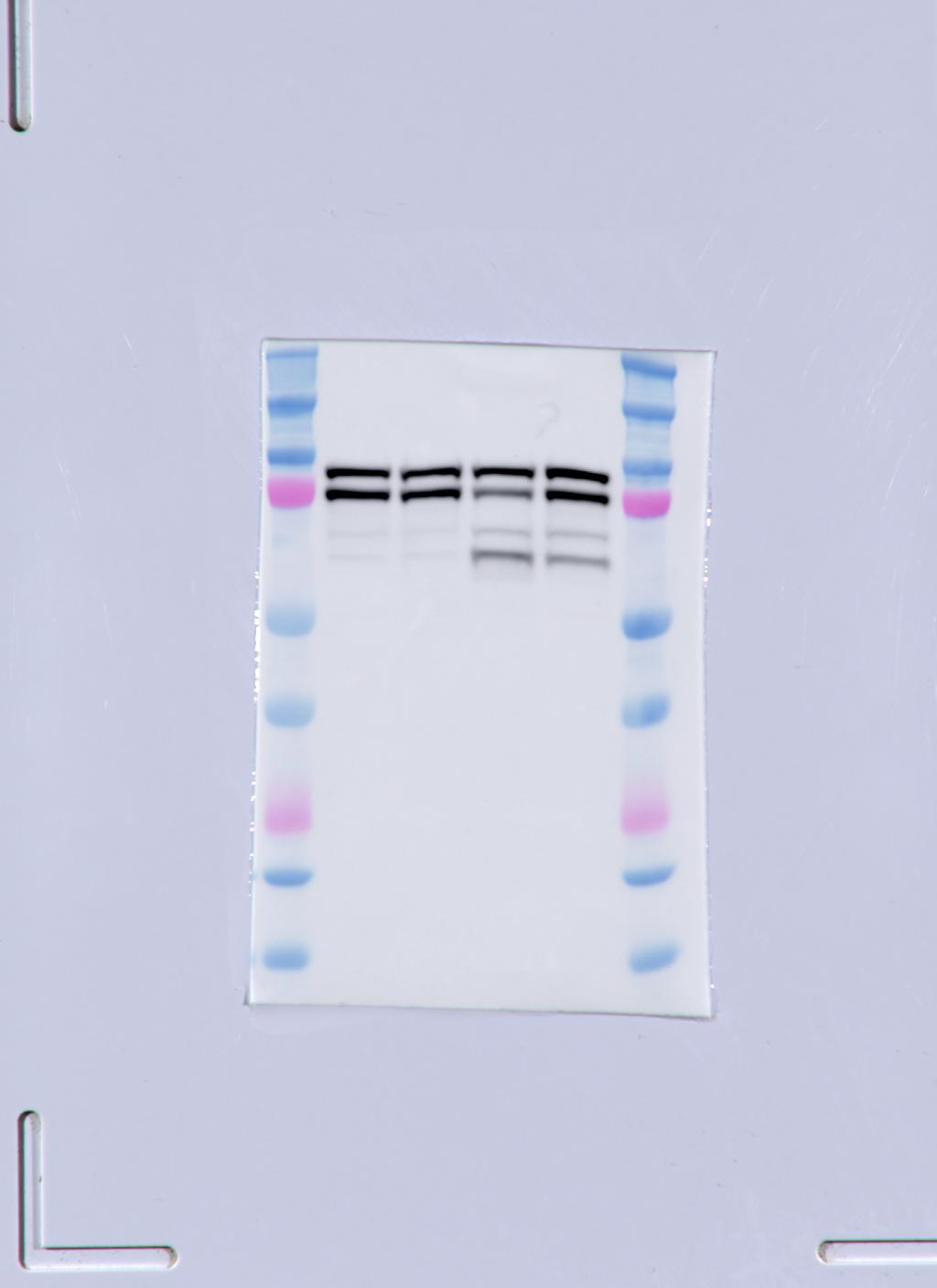

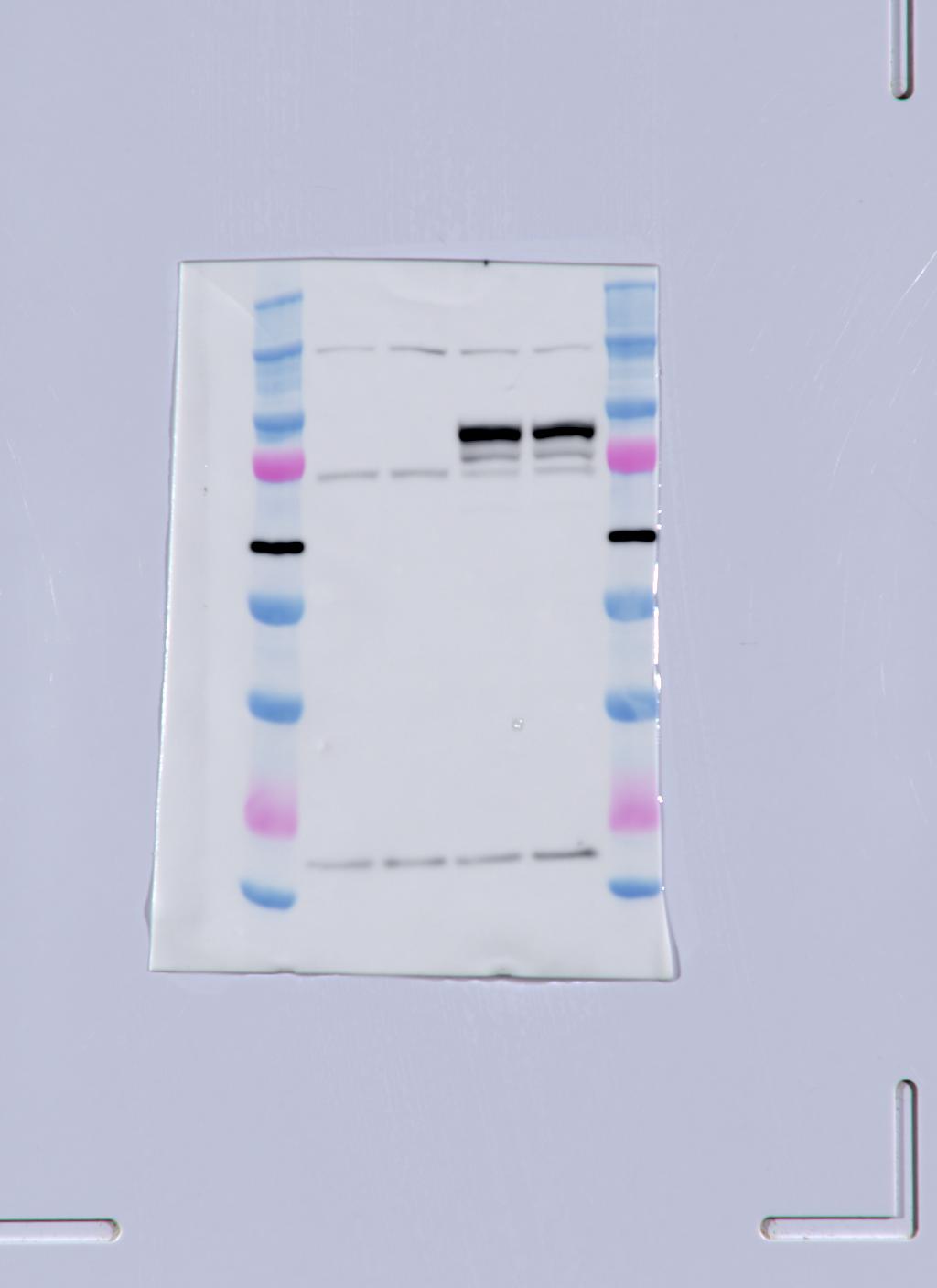

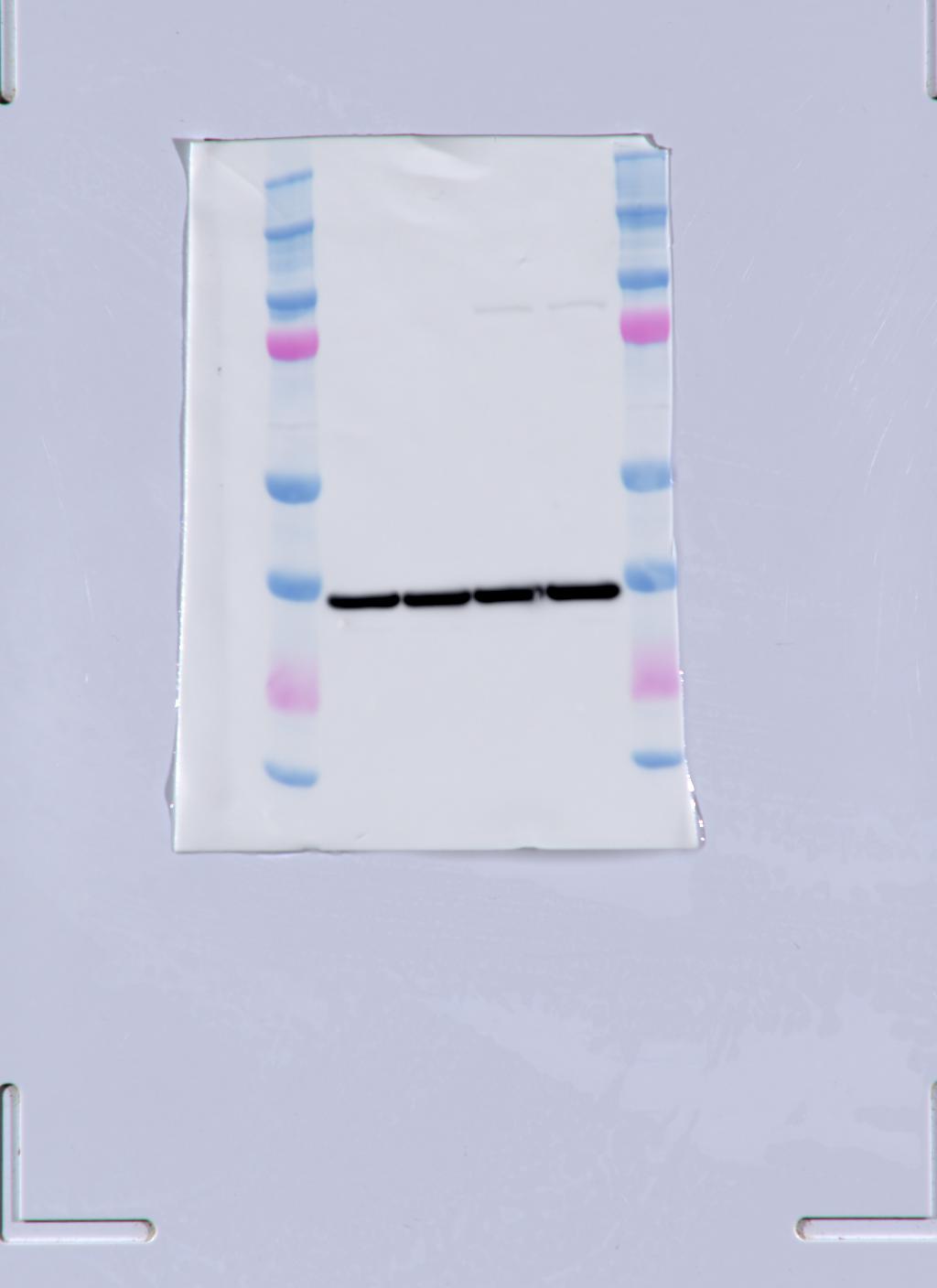
**

**（PDE4D;PDE4B;GAPDH）**

**
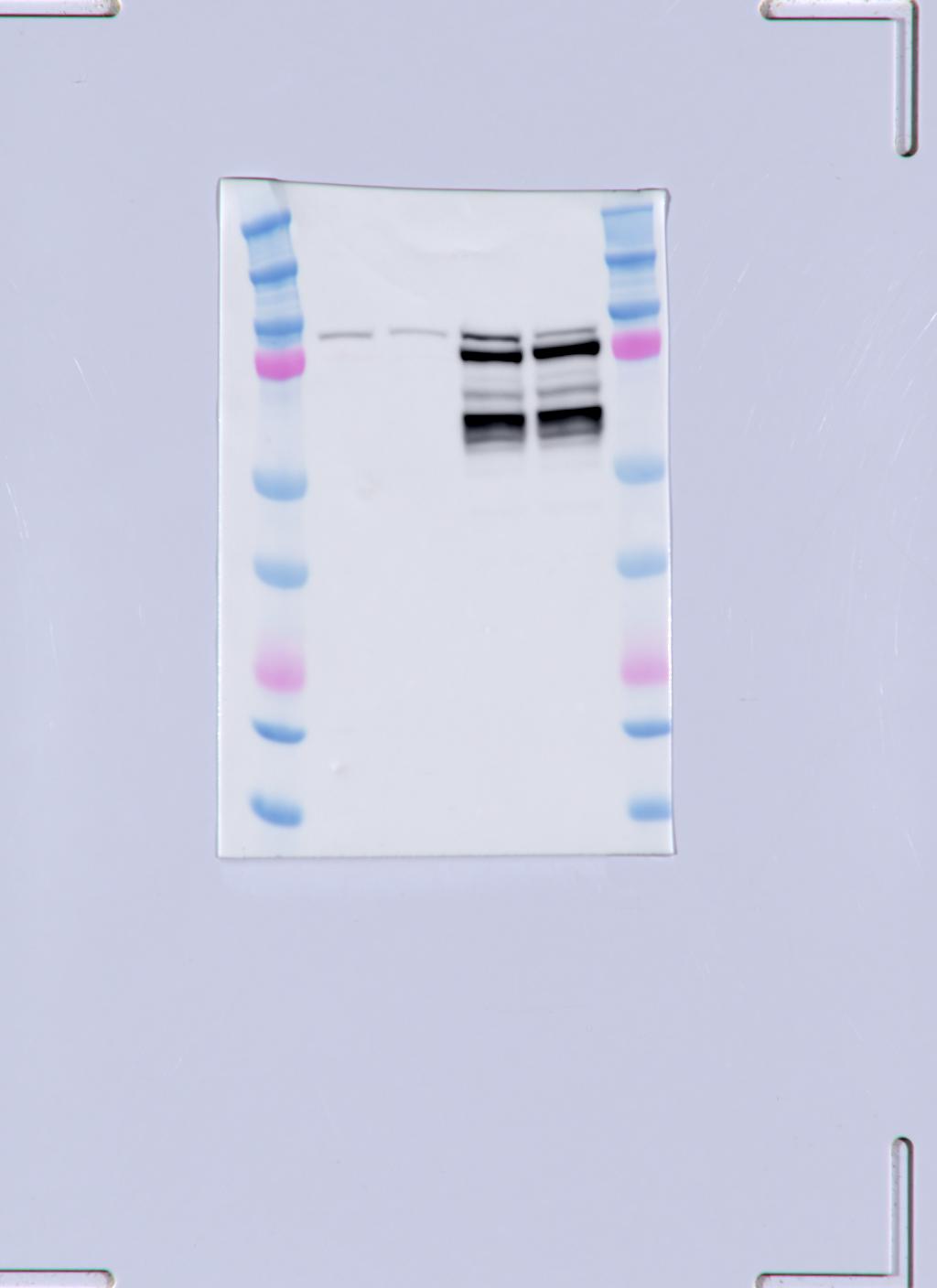

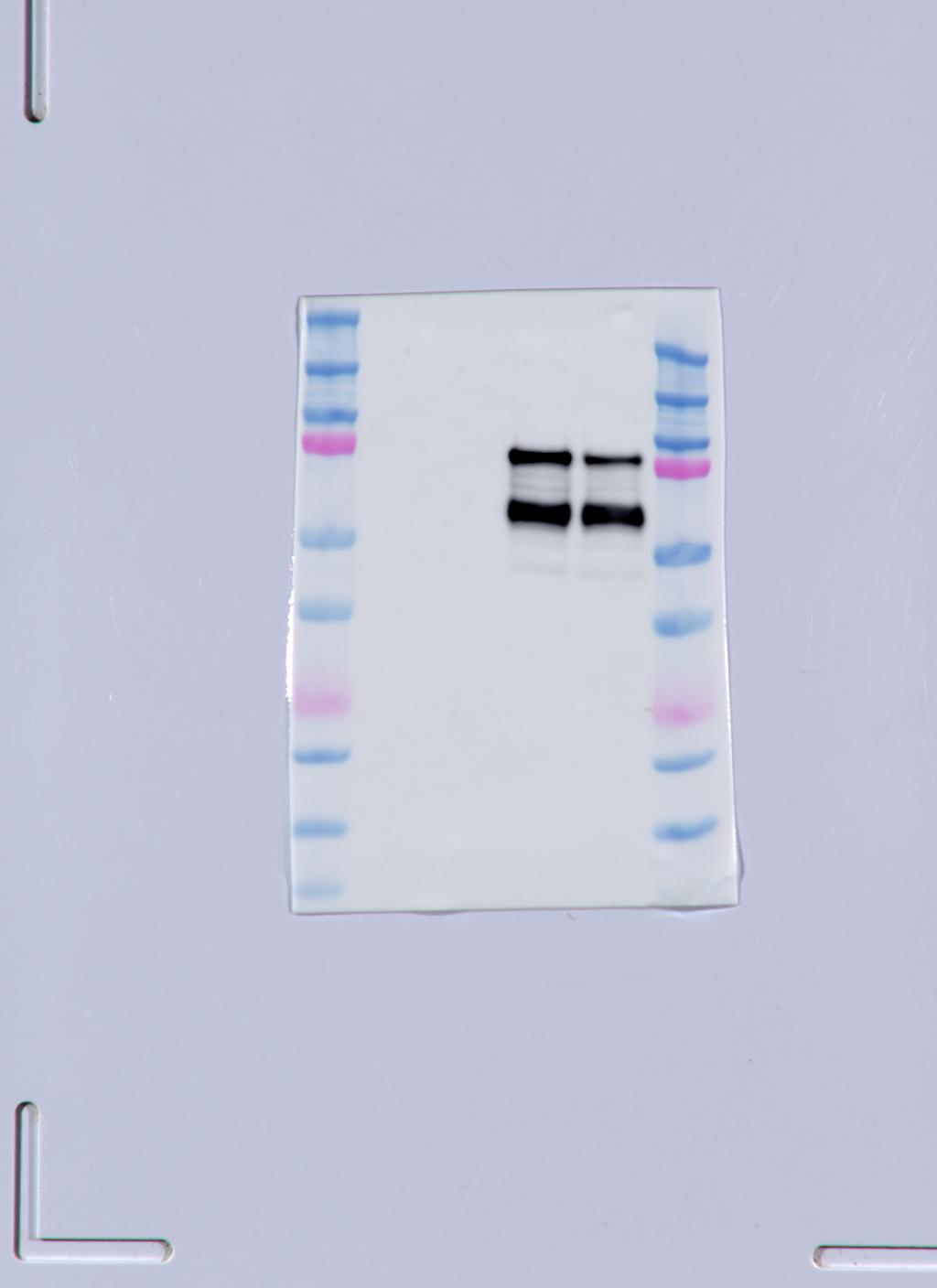

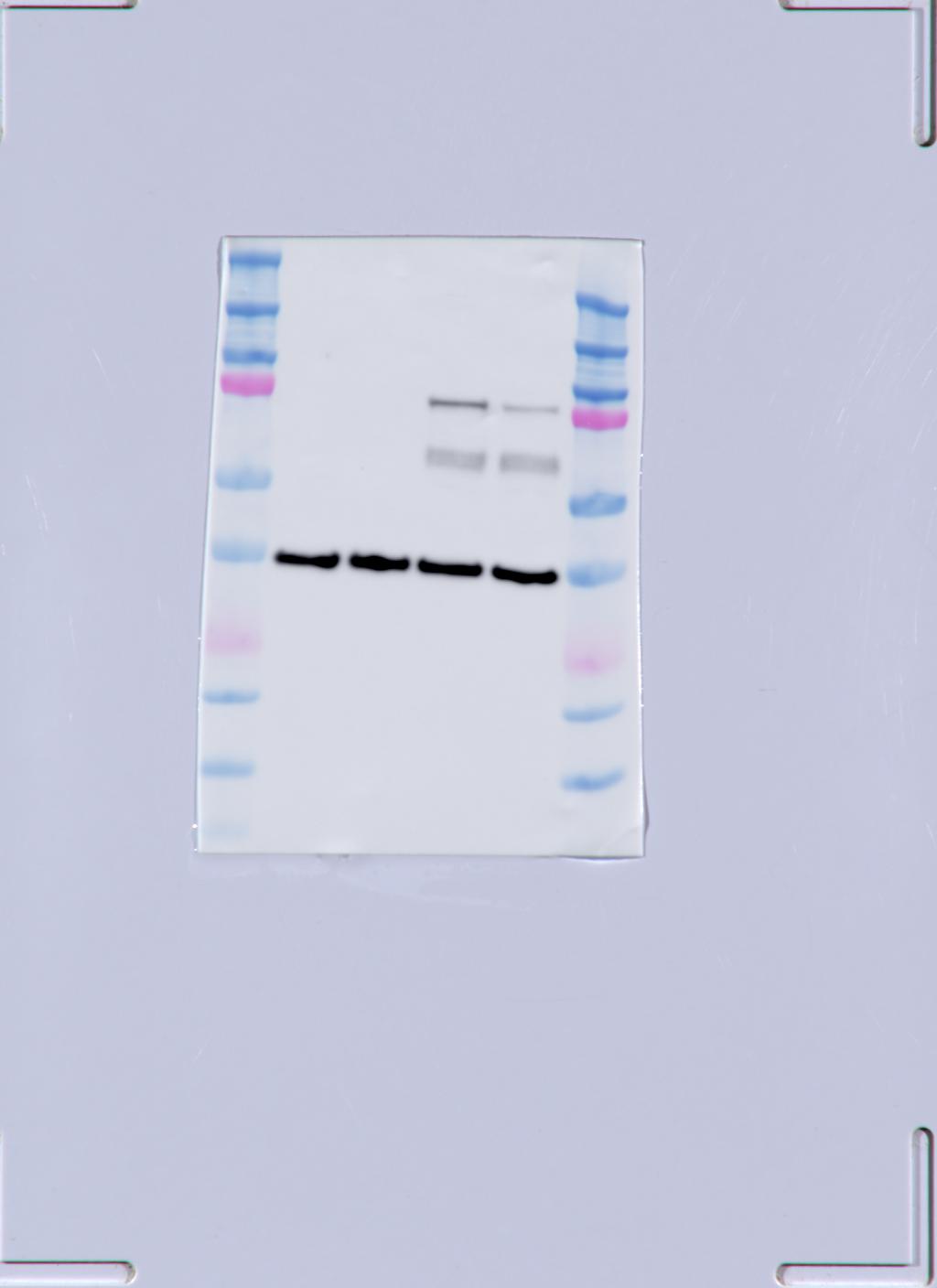
**

**Fig. S1c（STK11;GAPDH）**

**
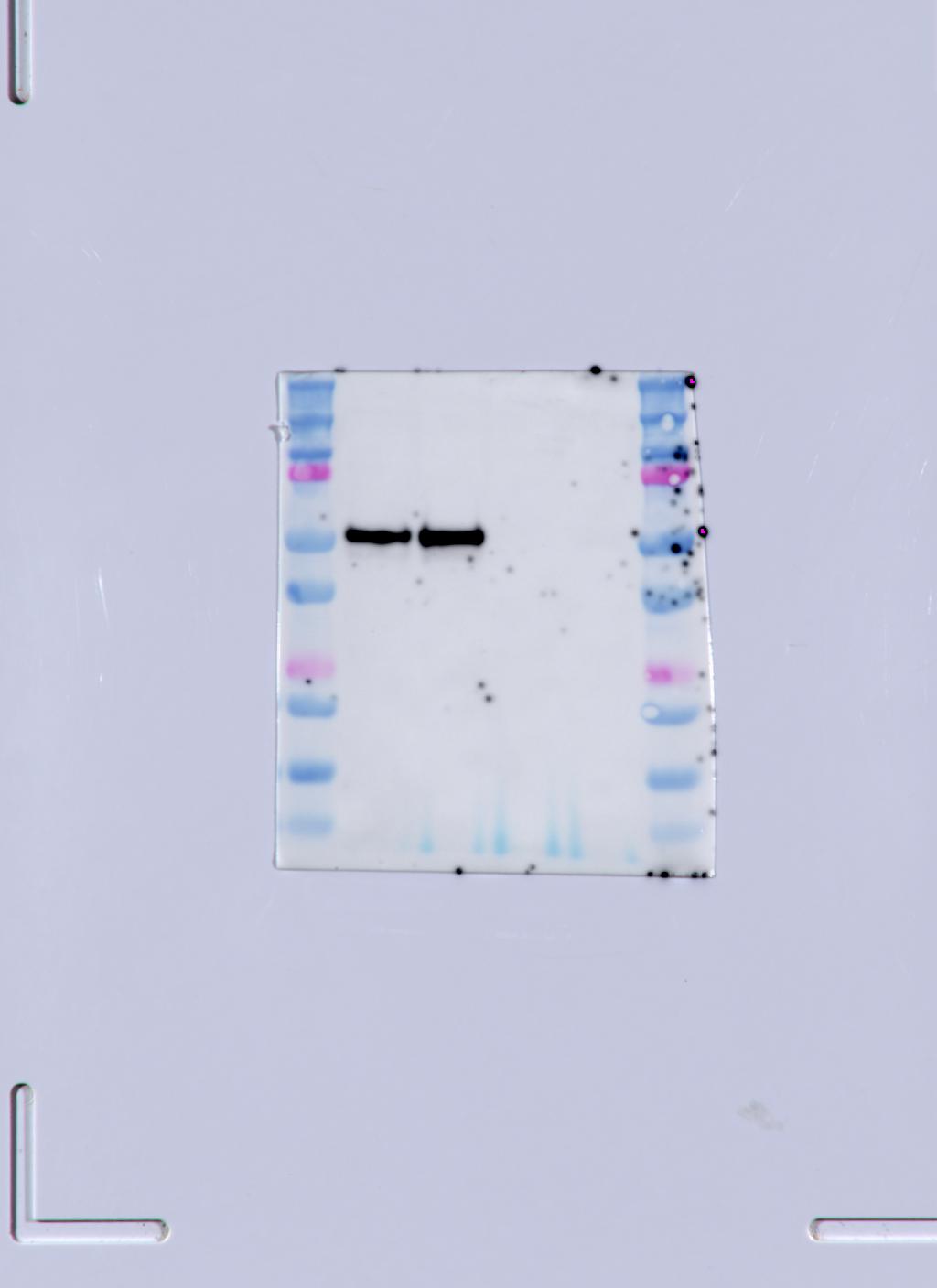

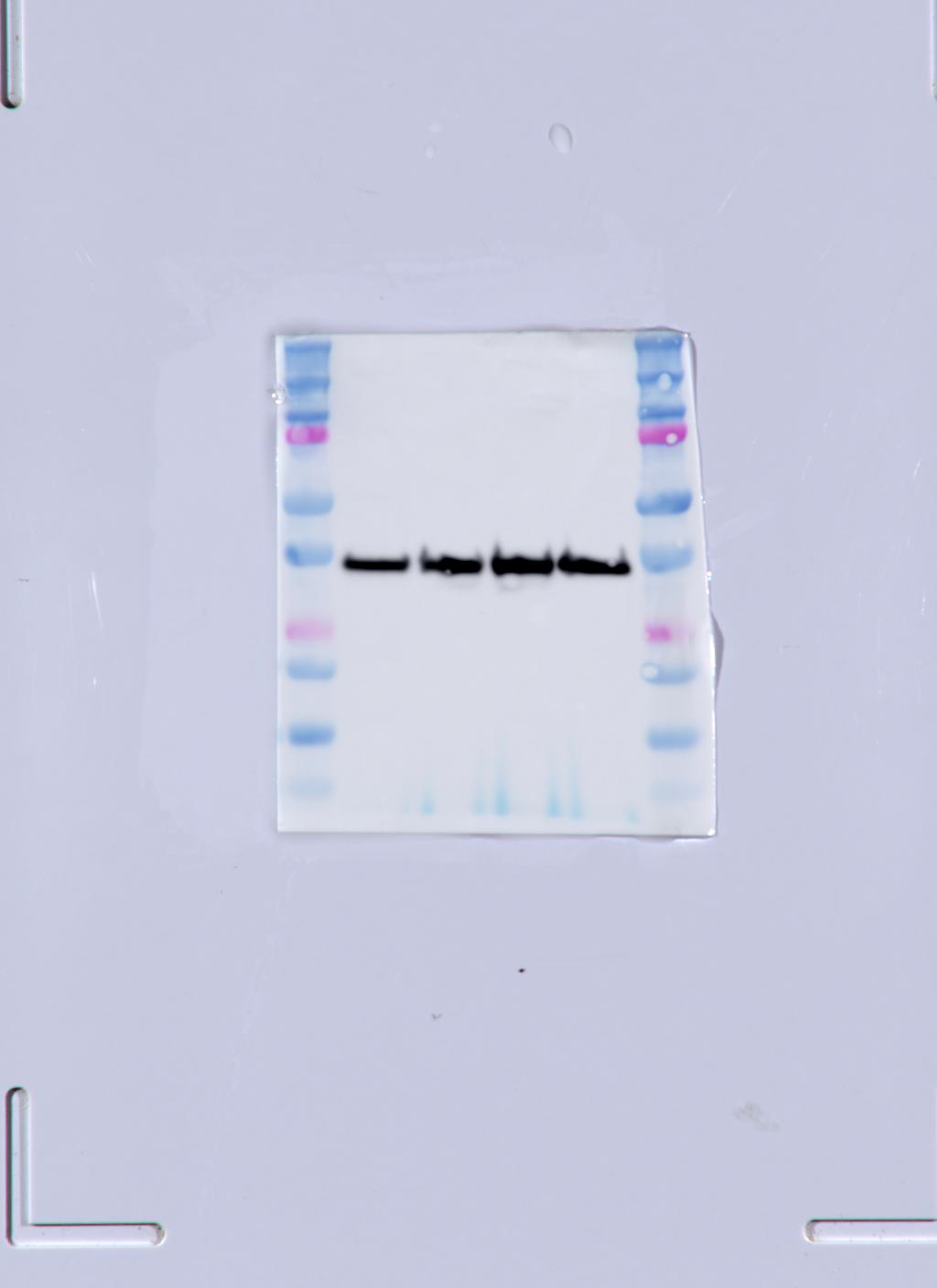
**

**Fig. S1d（STK11;GAPDH）**

**
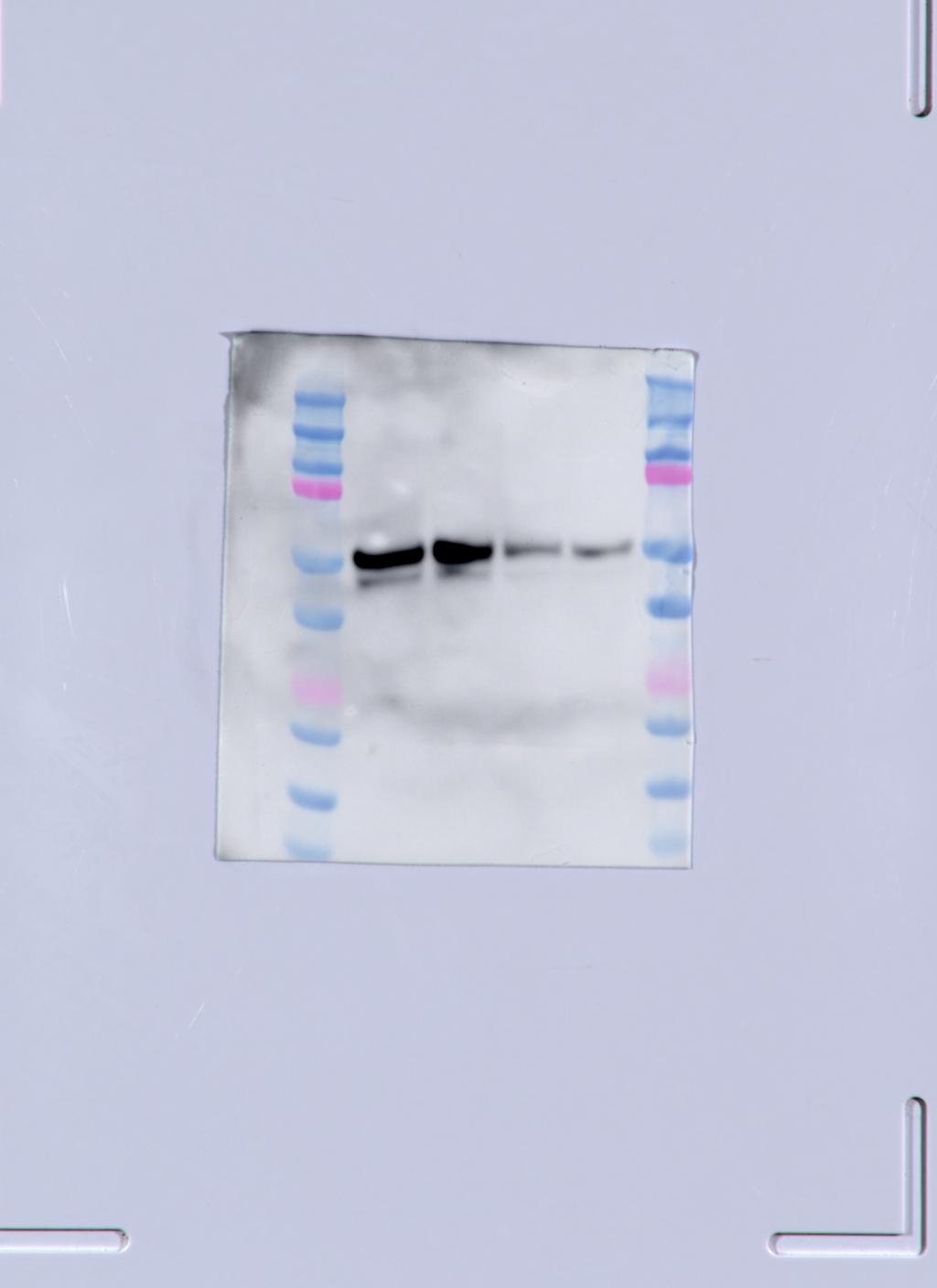

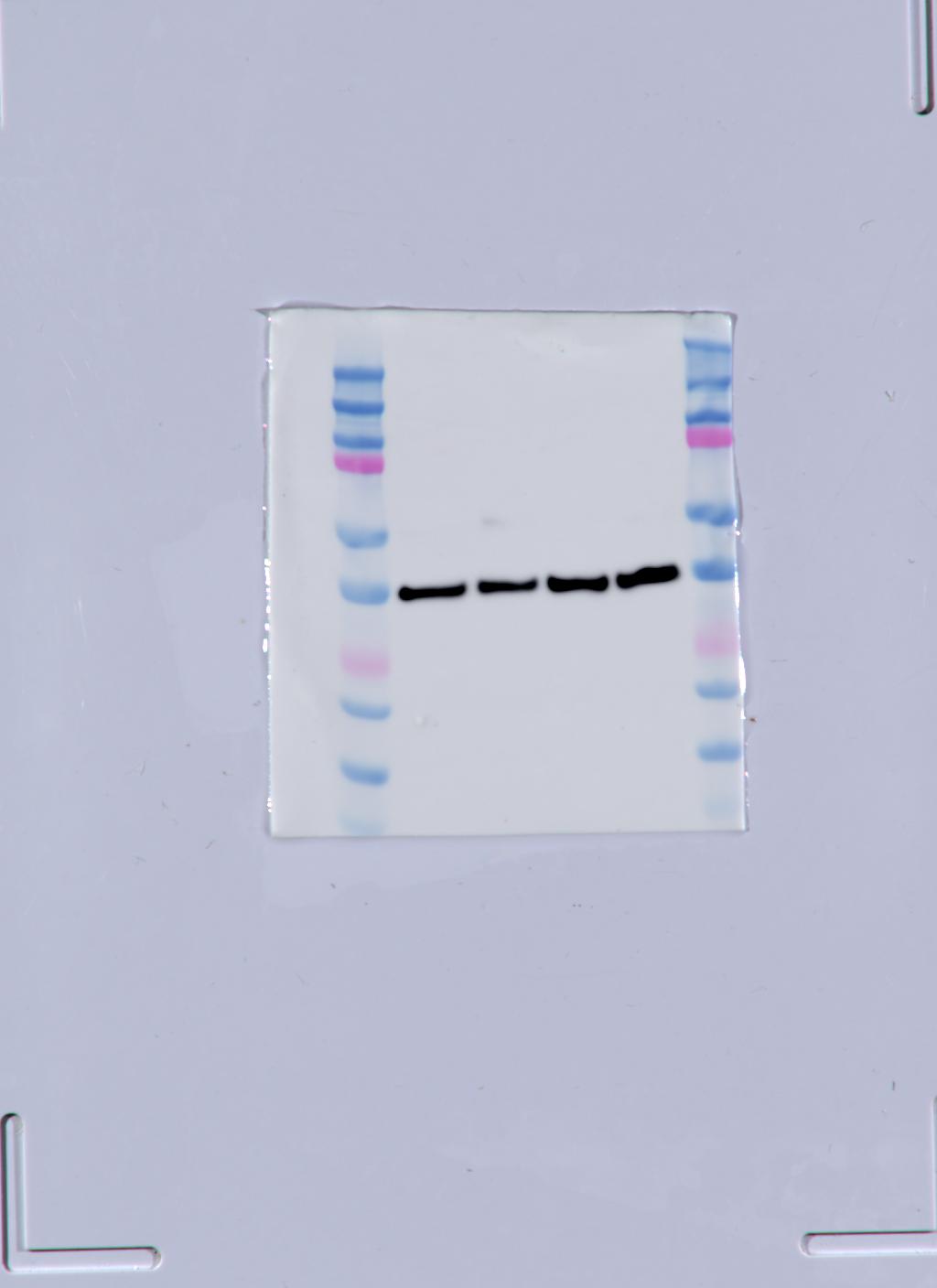
**

**Fig. S5o（STK11;Sox17;IL33）**

**
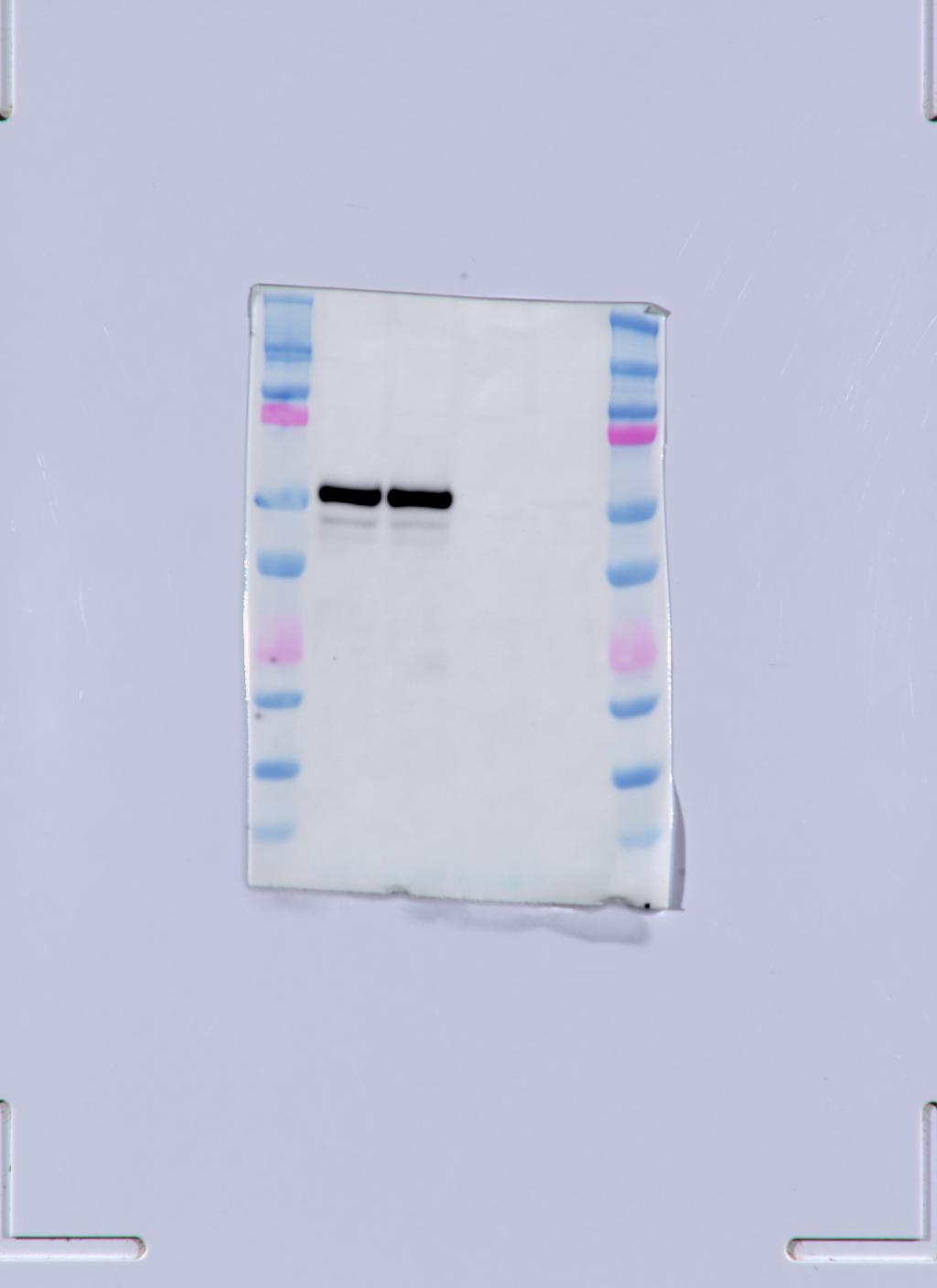

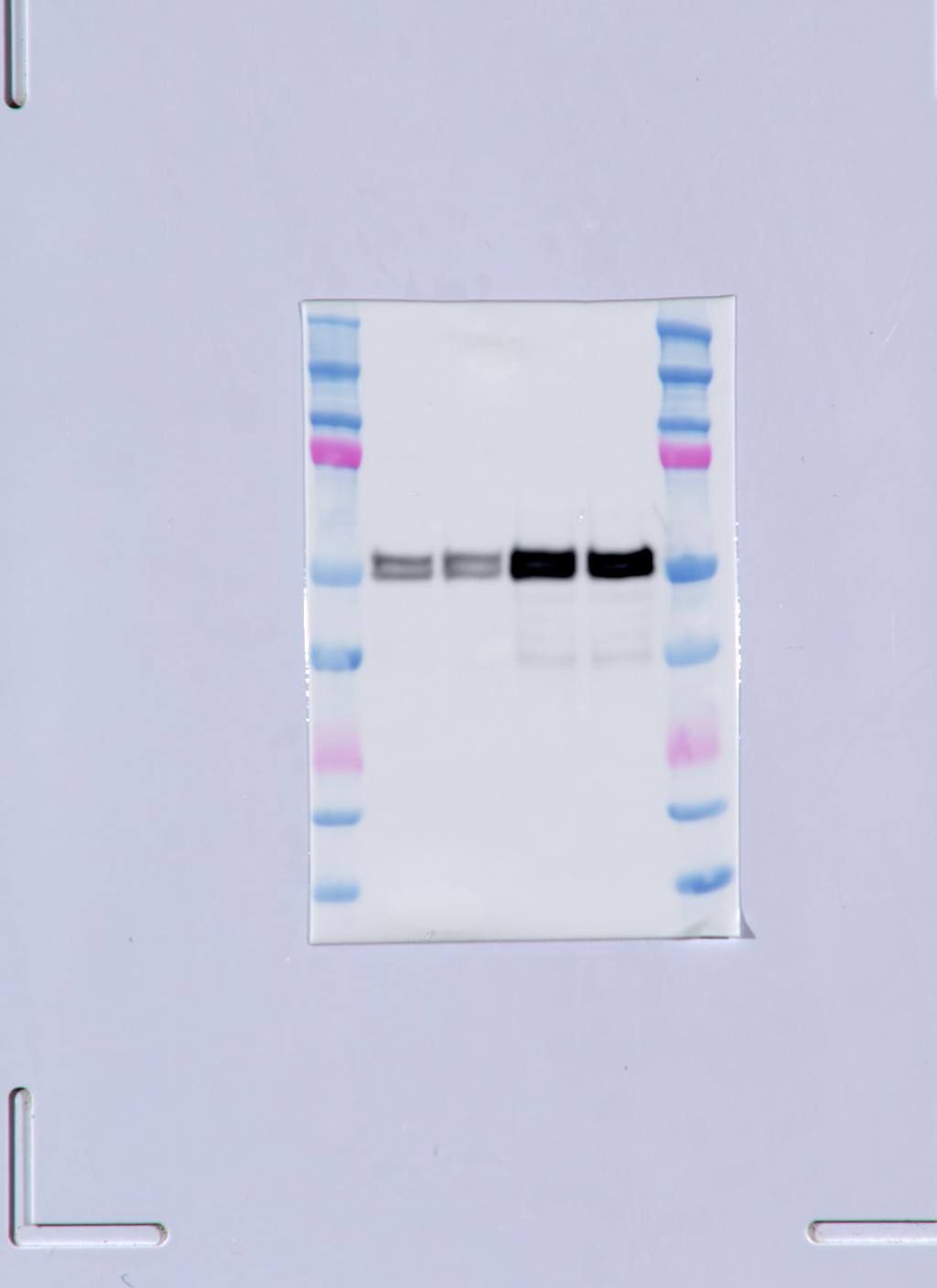

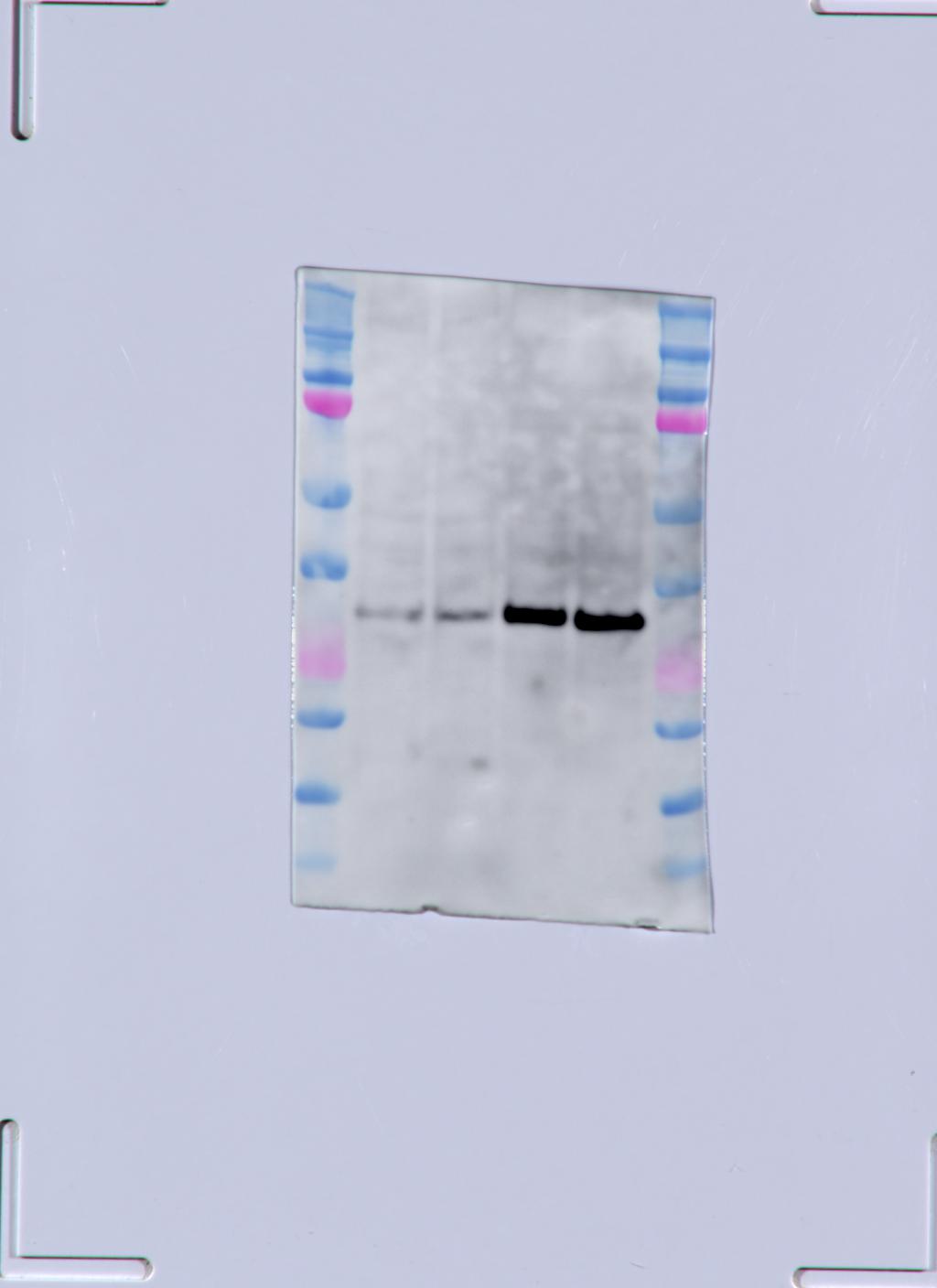
**

**（PDE4D;GAPDH）**

**
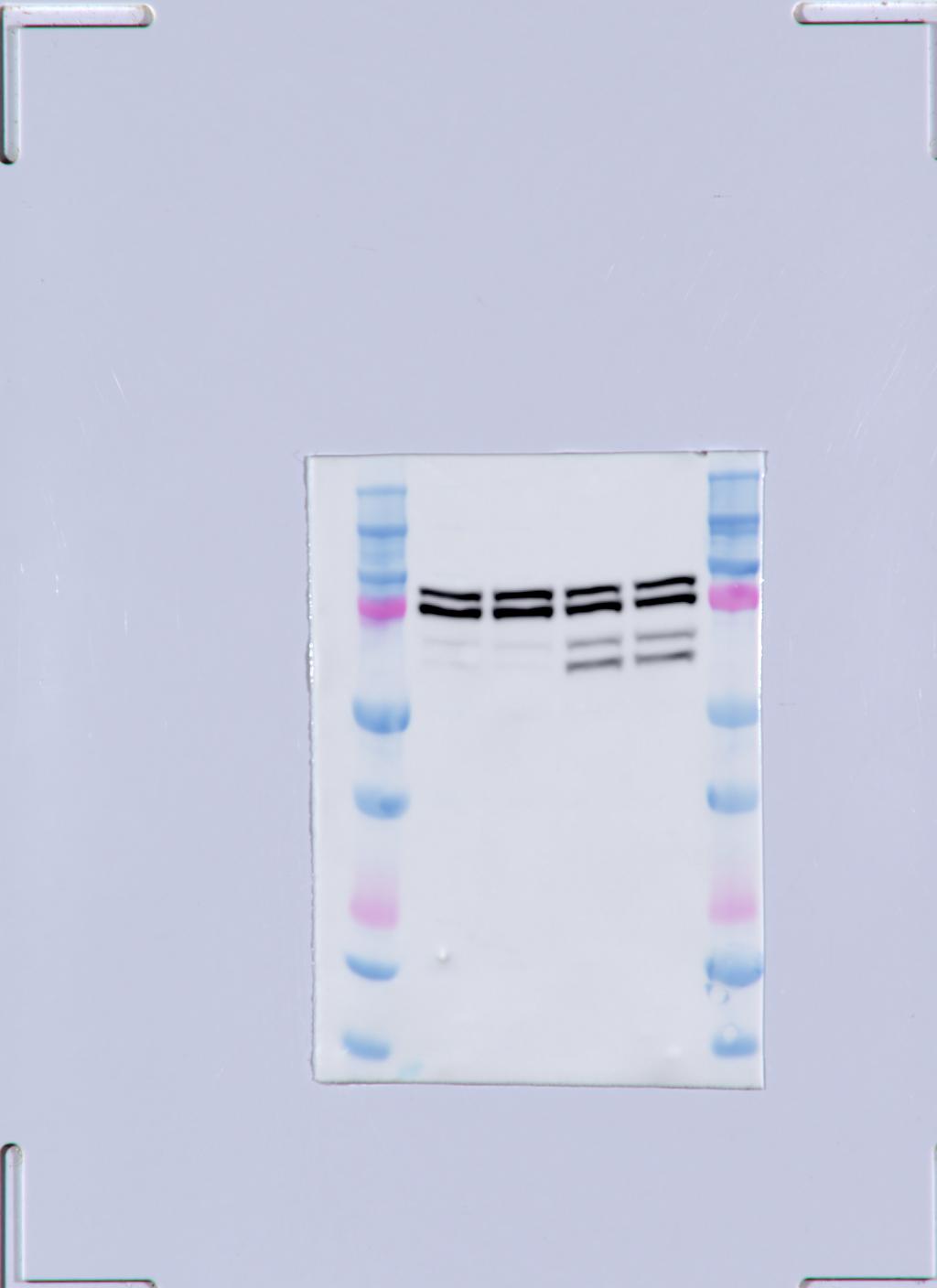

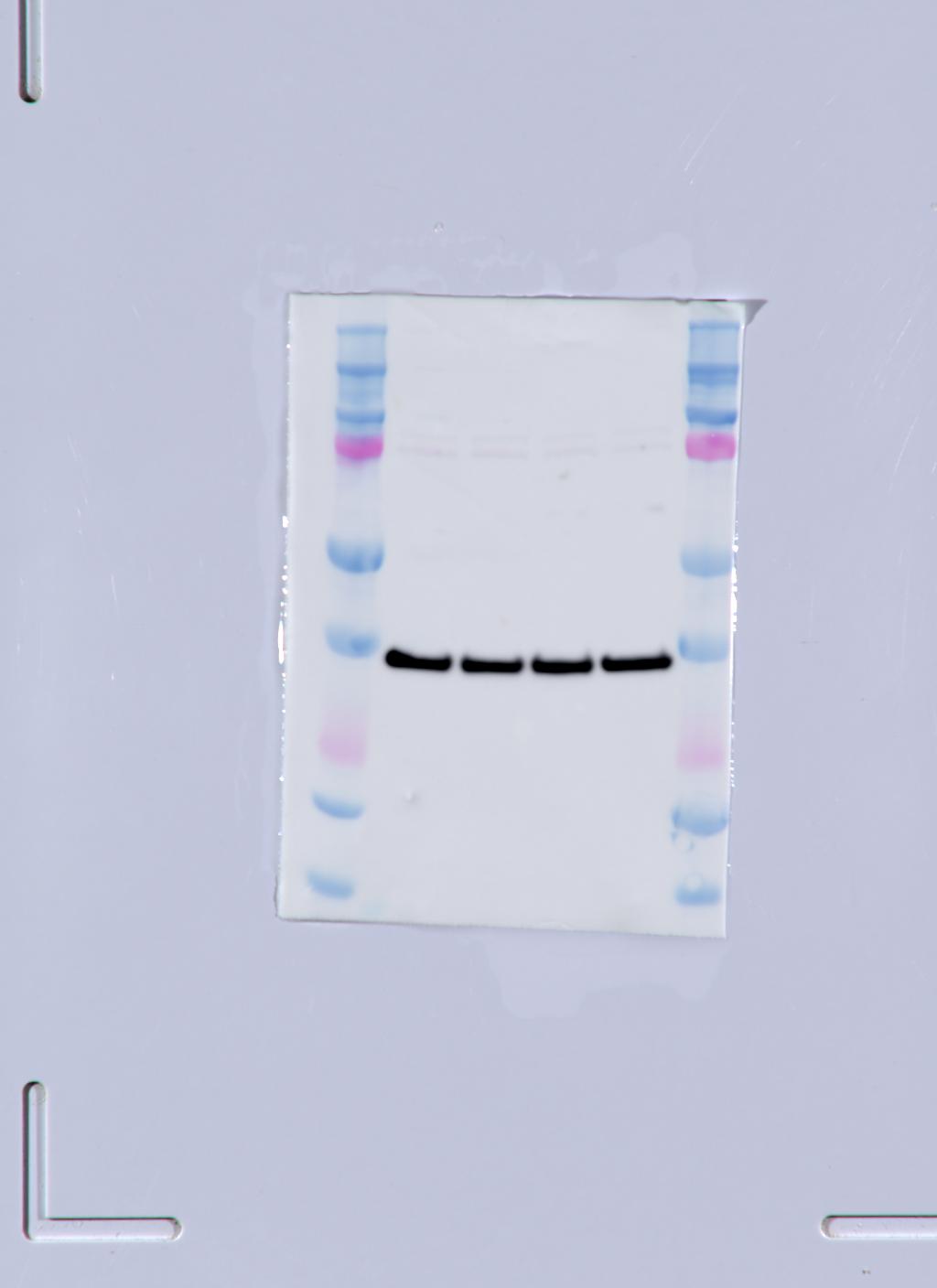
**

**Fig. S5p（STK11;PDE4B;GAPDH）**

**
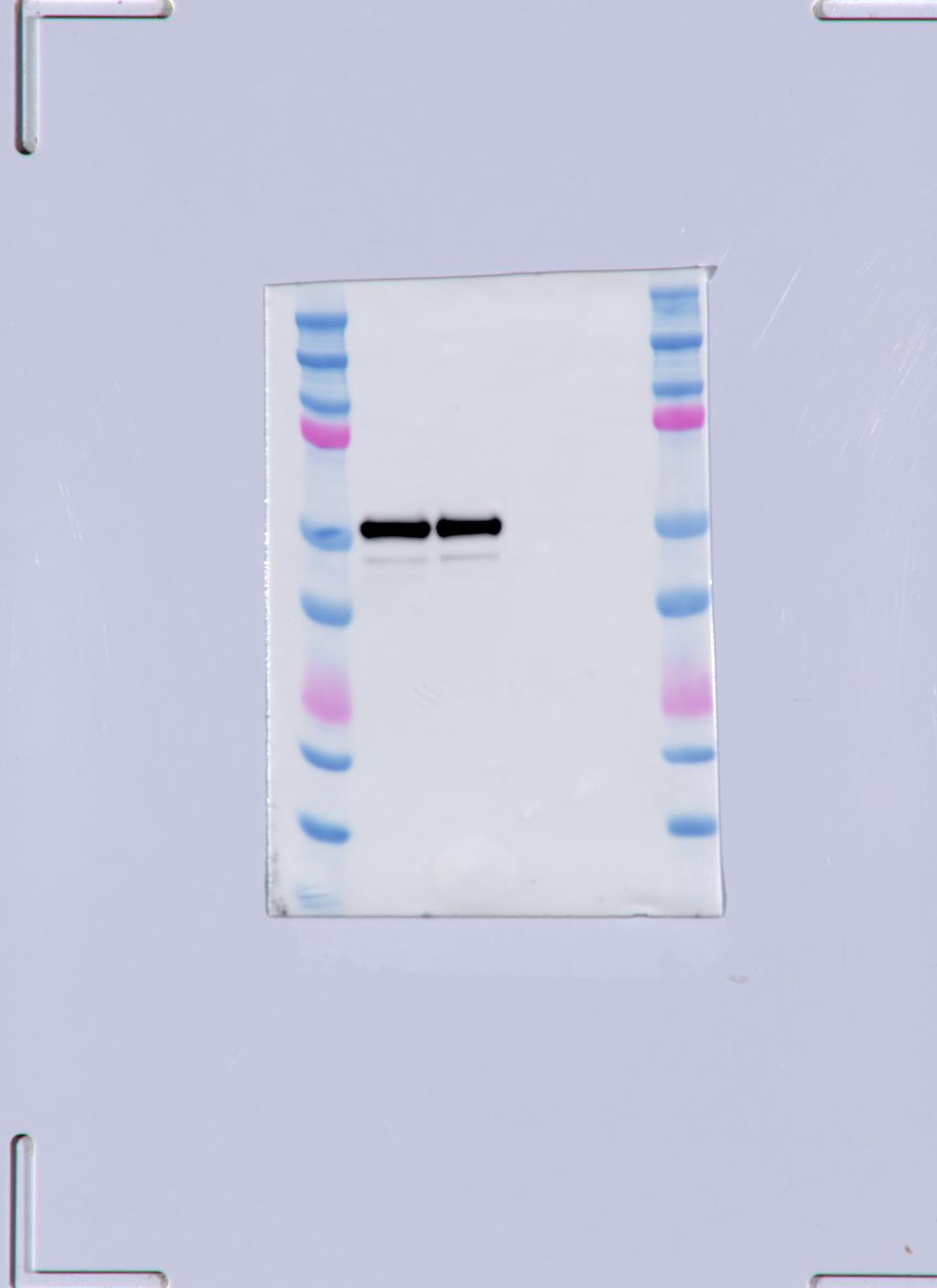

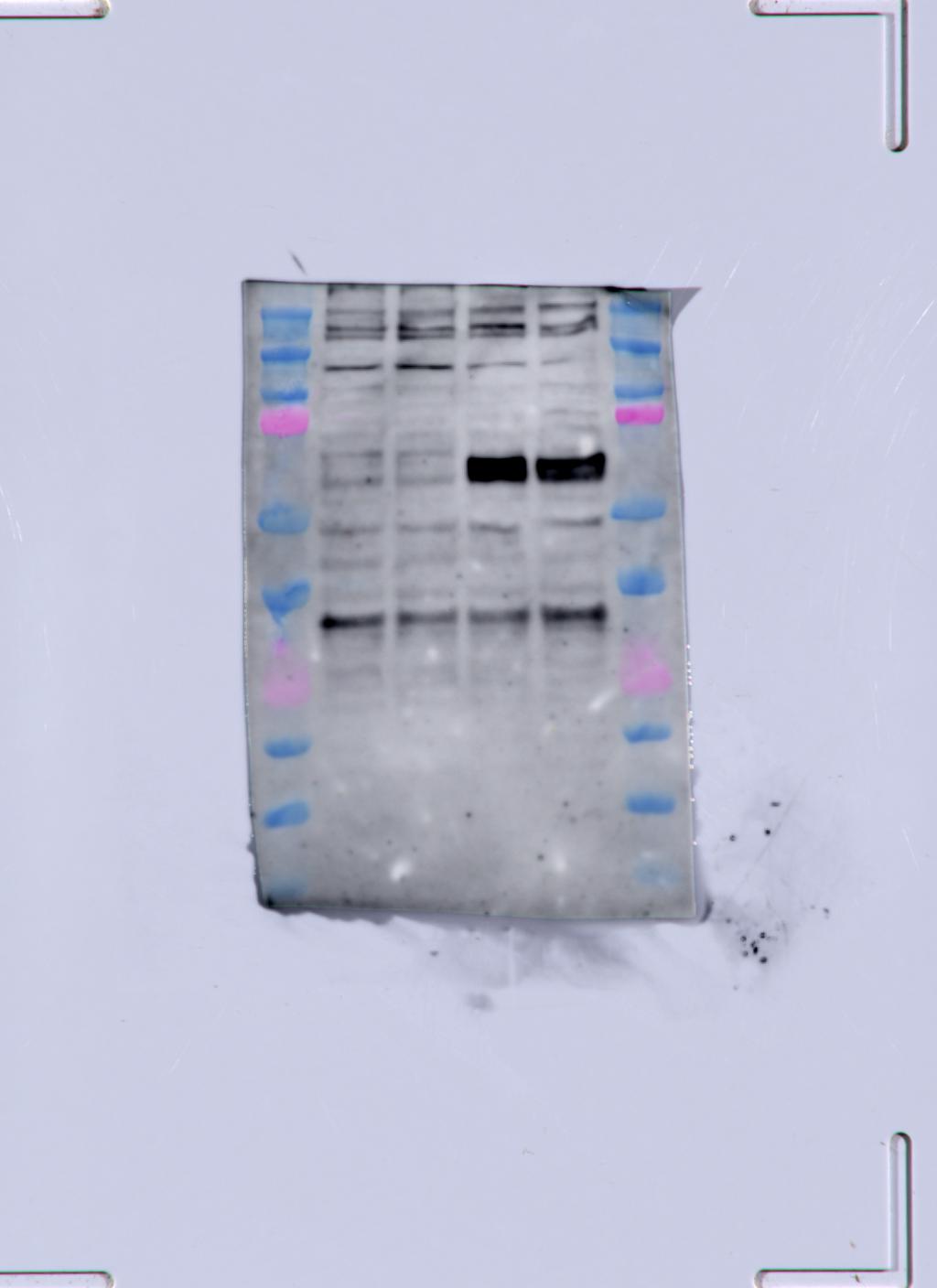

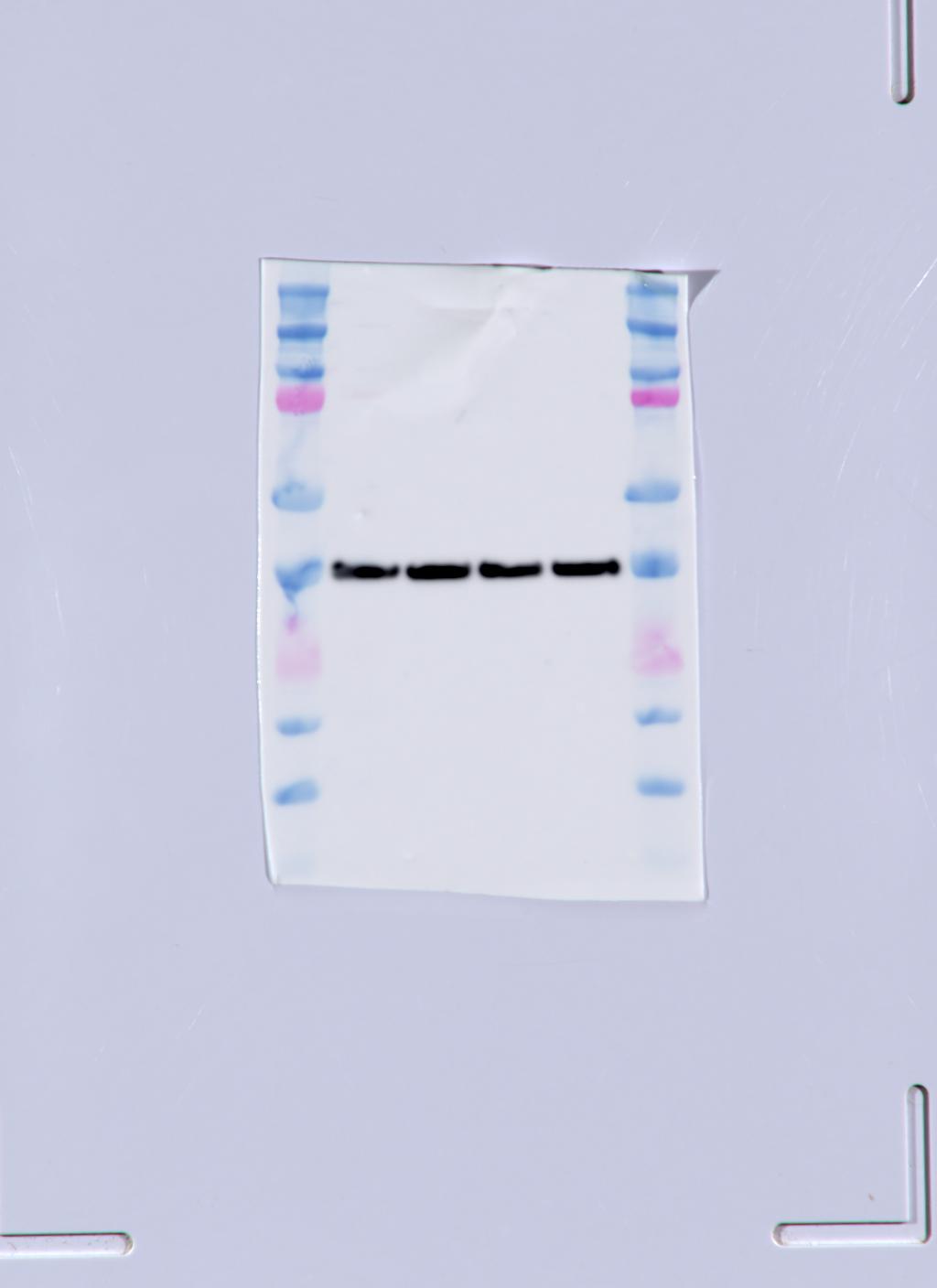
**

**Fig. S5q（STK11;PDE4D;PDE4B）**

**
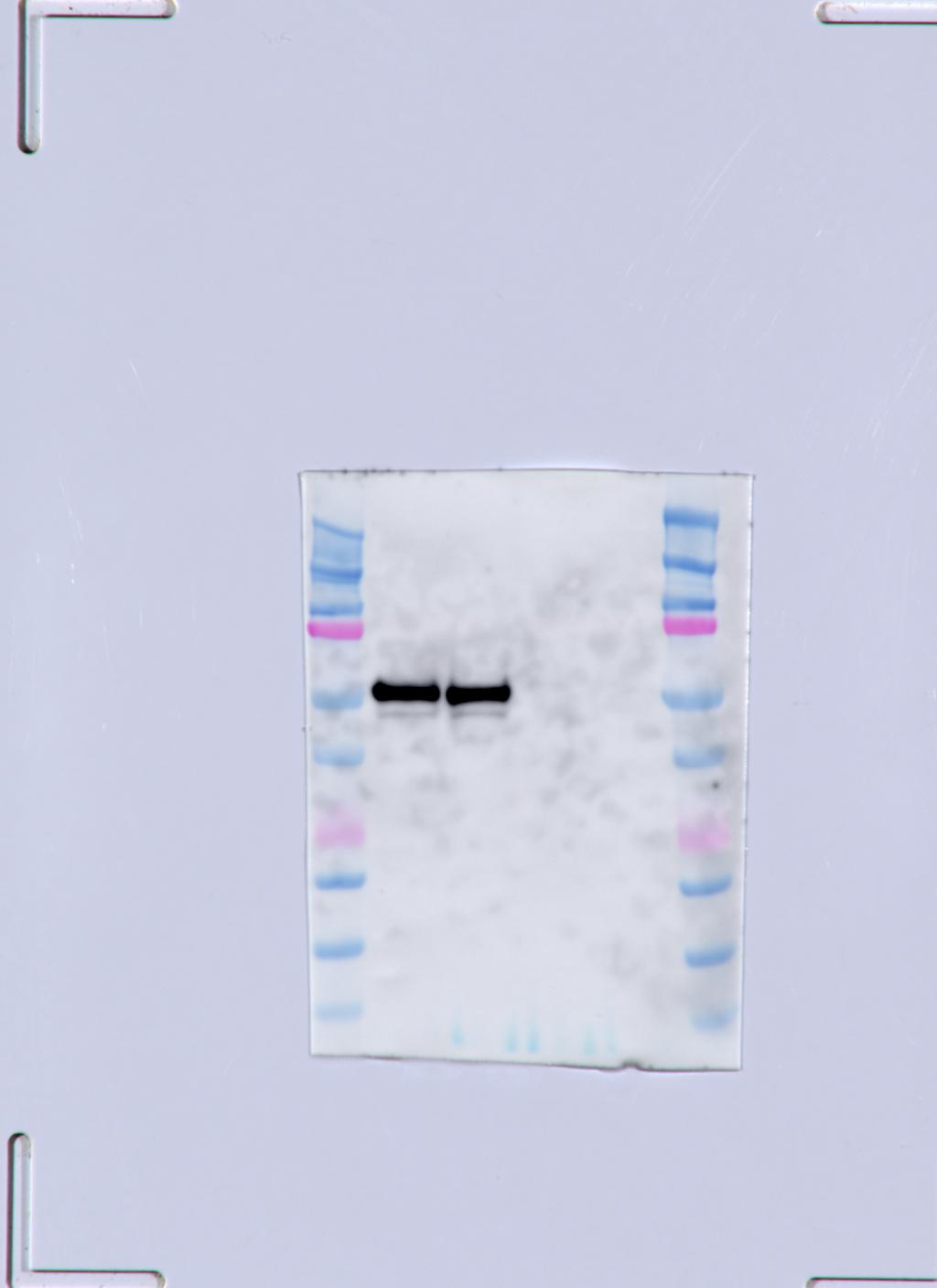

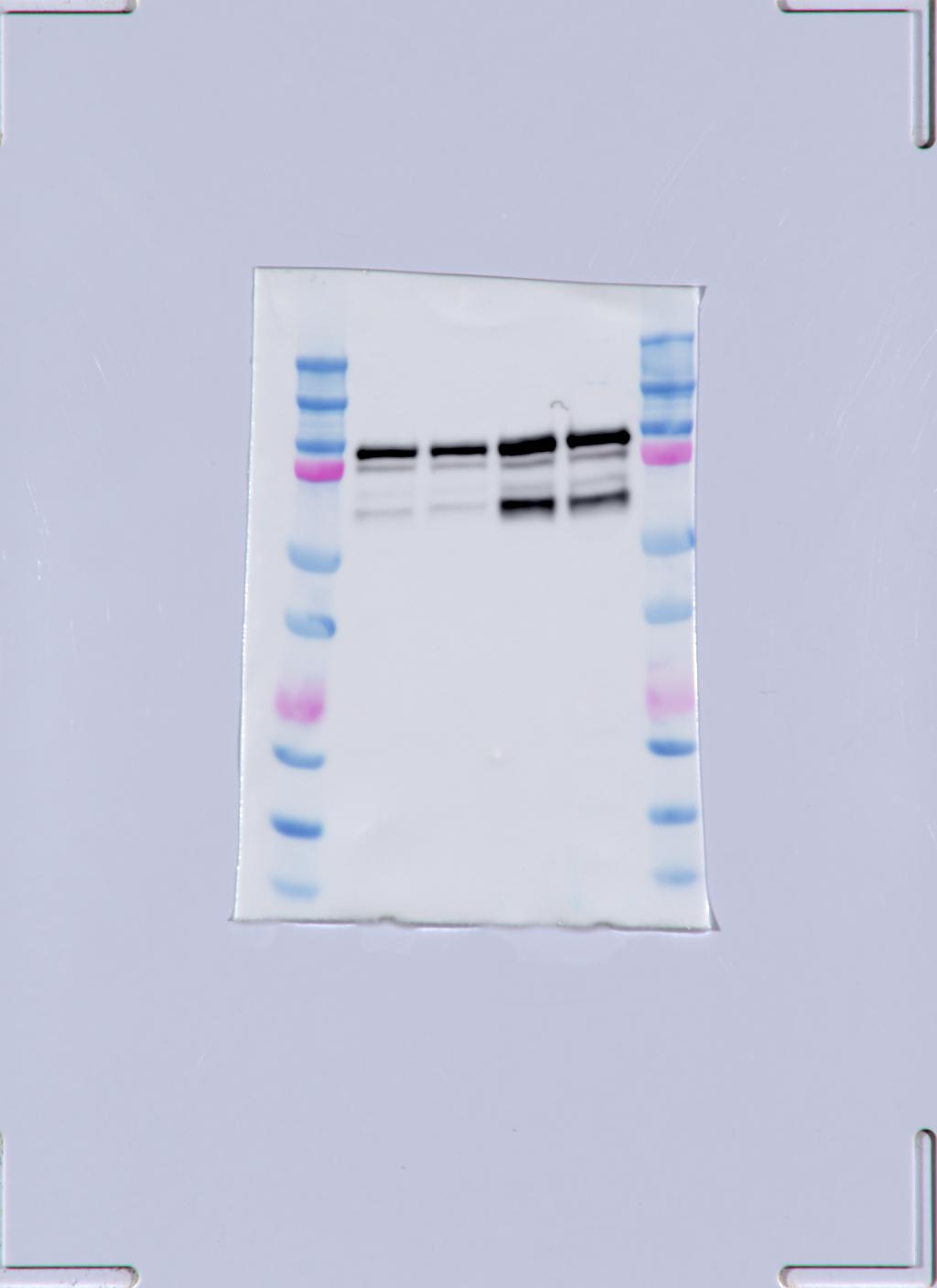

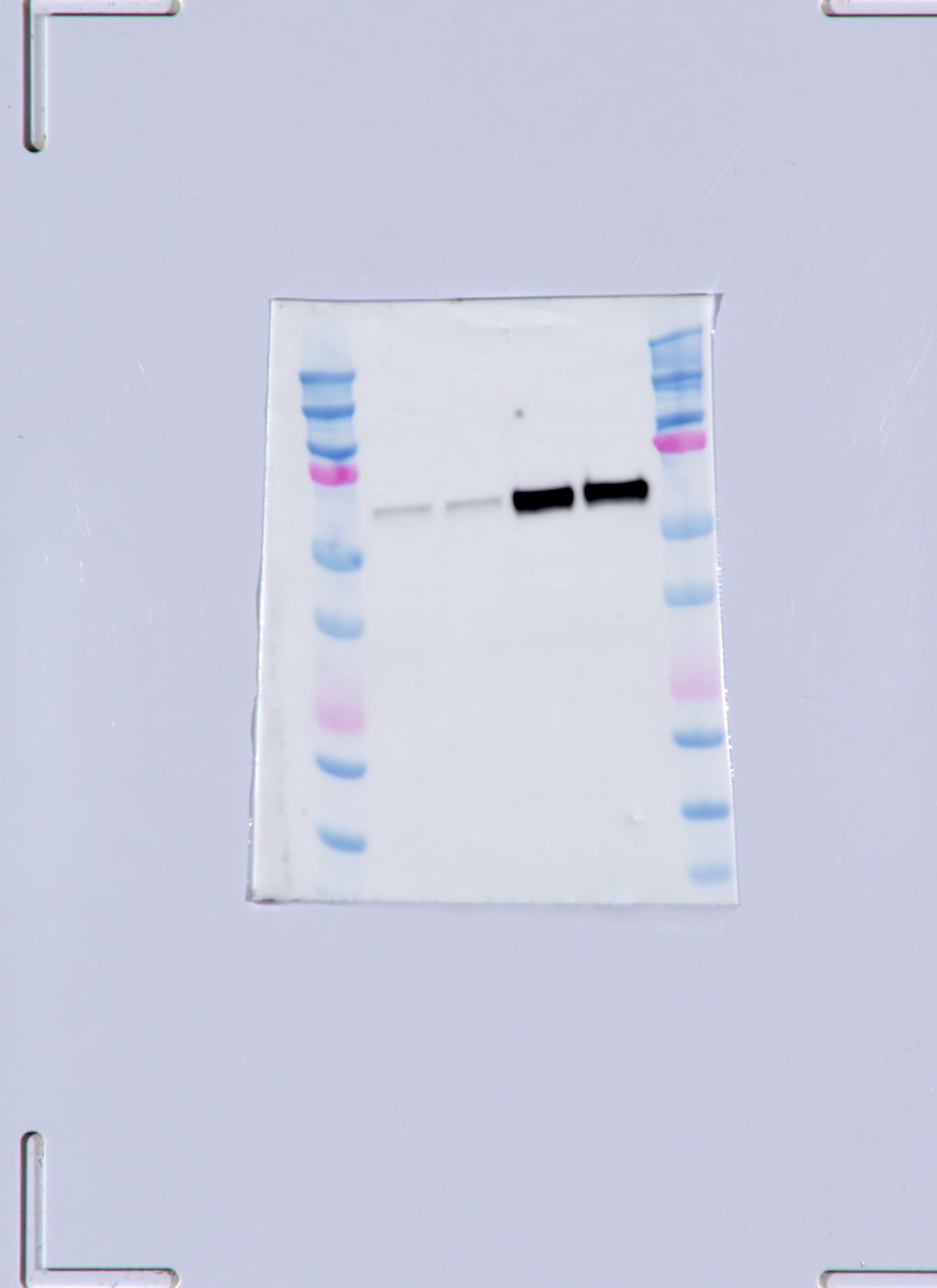
**

**(PDE10A;GAPDH)**

**
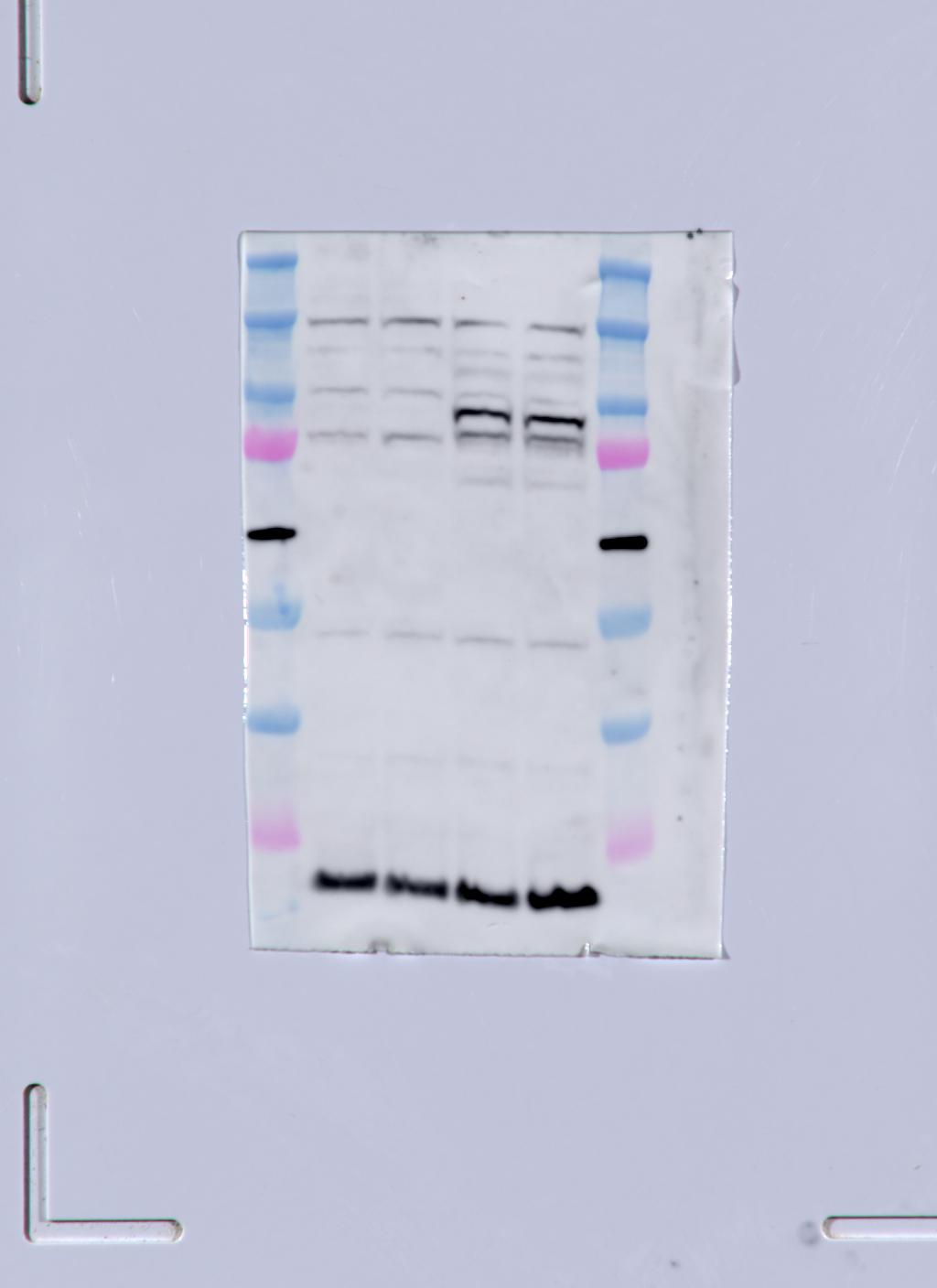

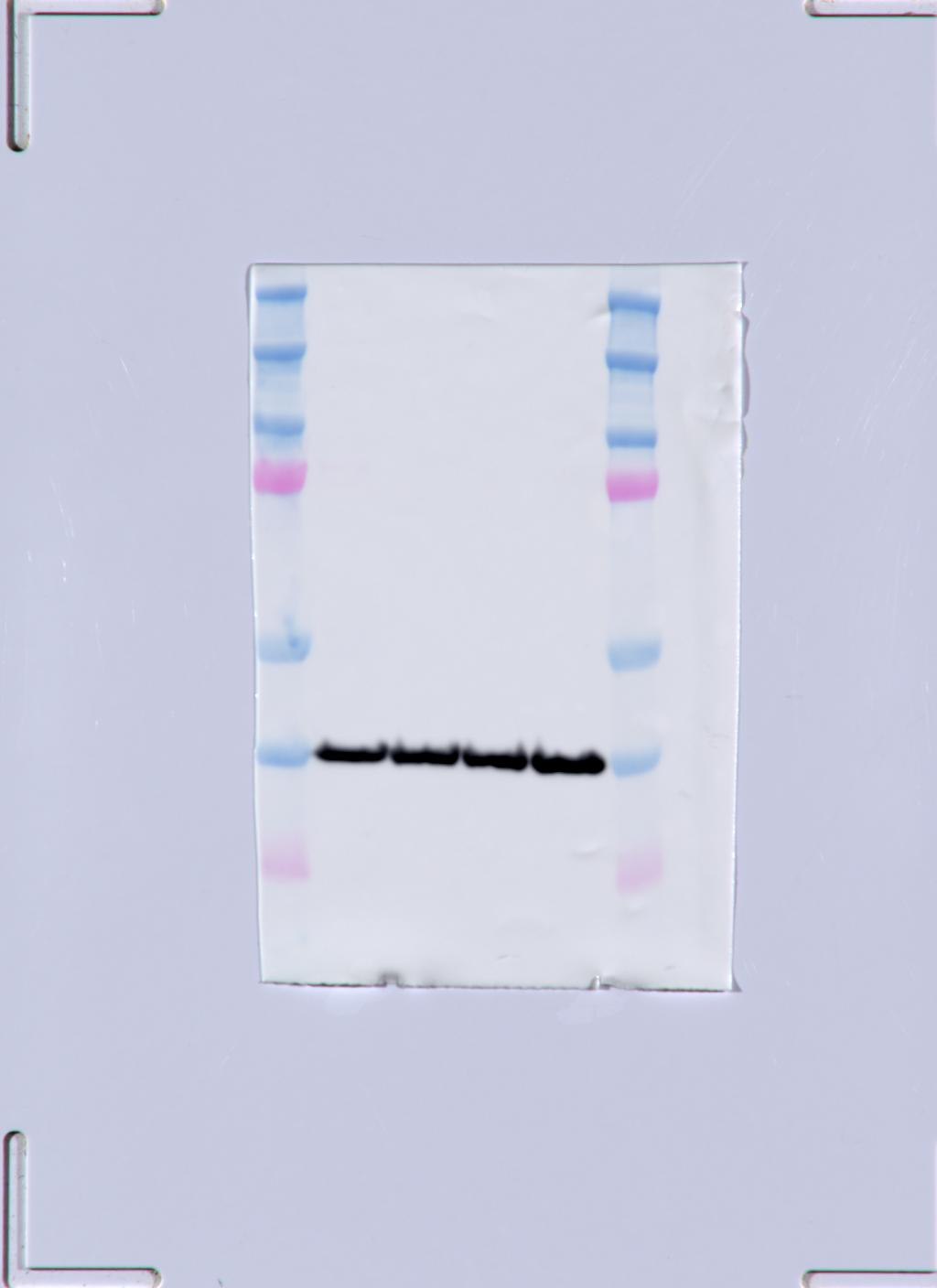
**
